# Non‐Amphiphilic Antimicrobial Polymers

**DOI:** 10.1002/anie.202507564

**Published:** 2025-07-07

**Authors:** Alain M. Bapolisi, Anne‐Catherine Lehnen, Martin Wolff, Jana Kramer, Sergio Kogikoski, René Steinbrecher, Nicole Michler, Andreas Kiesow, Ilko Bald, Martina Obry, Sebastian Kersting, Till Stensitzki, Henrike M. Müller‐Werkmeister, Meike N. Leiske, Salvatore Chiantia, Matthias Hartlieb

**Affiliations:** ^1^ Institute of Chemistry University of Potsdam Karl‐Liebknecht‐Straße 24–25 14476 Potsdam Germany; ^2^ Fraunhofer Institute for Applied Polymer Research (IAP) Geiselbergstraße 69 14476 Potsdam Germany; ^3^ Institute of Physical Biochemistry University of Potsdam Karl‐Liebknecht‐Straße 24–25 14476 Potsdam Germany; ^4^ Fraunhofer‐Institut für Mikrostruktur von Werkstoffen und Systemen IMWS Walter‐Hülse‐Str. 1 06120 Halle (Saale) Germany; ^5^ Fraunhofer Institute for Cell Therapy and Immunology Branch Bioanalytics and Bioprocesses (IZI‐BB) Am Mühlenberg 13 14476 Potsdam Germany; ^6^ Faculty of Biology Chemistry & Earth Sciences University of Bayreuth Universitätsstraße 30 95447 Bayreuth Germany; ^7^ Bavarian Polymer Institute Universitätsstraße 30 95447 Bayreuth Germany

**Keywords:** Amphiphilicity, Antimicrobial polymers, Hydrogen bonding, Membrane interaction, RAFT polymerization

## Abstract

Antimicrobial resistance (AMR) is a severe threat to modern healthcare and must be addressed to prevent millions of deaths in the coming decades. Antimicrobial polymers (APs) do not provoke resistance and are promising alternatives to conventional antibiotics. Classic APs possess an amphiphilic structure (cationic and hydrophobic). Herein, we question the necessity of amphiphilicity in APs and find that hydrophobicity is not an essential quality in these polymers. Combining cationic monomers with hydrophilic subunits containing hydrogen bond donors results in excellent antibacterial activity and concurrently low unspecific toxicity. Non‐amphiphilic APs have the unique ability to cluster in isolated membrane regions, creating a supramolecular multivalence that enhances their membrane activity and aggregates bacterial cells. This effect, which only unfolds in the absence of hydrophobicity, opens new possibilities in the design of antimicrobial materials.

## Graphical abstract

Completely non‐amphiphilic antimicrobial polymers (APs) that possess significant bioactivity and excellent selectivity are presented. The importance of hydrogen‐bonding interactions is highlighted by segregation of APs on bacterial membrane models and bacteria, a behavior absent in amphiphilic polymers. A supramolecular multivalence is proposed that leads to increased membrane activity without the necessity for hydrophobicity.

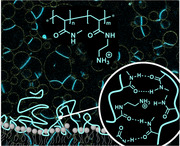

## Introduction

With well over 1 million deaths in 2019 directly related to antimicrobial resistance (AMR),^[^ ^1^ ^]^ humanity is at the onset of a post‐antibiotic era.^[^ ^2^ ^]^ When antibiotics fail, a plethora of medical procedures become increasingly risky or even impossible, and casualties are estimated to rise drastically.^[^ ^3^ ^]^ The reasons for this development are manifold, including misuse of antibiotics in the food industry or overprescription.^[^ ^4^ ^]^ Moreover, any newly developed antibiotic is immediately declared a last‐resort drug, to be used only in dire cases, creating a disincentive for investment.^[^ ^5^ ^]^ The root cause for these problems is the specificity of antibiotics, making it easy for microorganisms to deploy countermeasures by, e.g., alteration in the target structure or by inactivating the drug.^[^ ^6^ ^]^ The development of new antimicrobials represents an important escape route. And while promising candidates are in development,^[^ ^7^, ^8^ ^]^ their target‐specific mode of action is likely to cause resistance eventually.^[^ ^9^ ^]^

Antimicrobial polymers (APs),^[^ ^10^, ^11^ ^]^ which were initially designed as mimics of host defense peptides (HDPs), have excellent prospects in this regard, as they are not susceptible toward resistance development.^[^ ^12^, ^13^ ^]^ They permeabilize the bacterial membrane as a result of their physicochemical properties (cationic charge + hydrophobicity).^[^ ^10^, ^11^, ^14^, ^15^ ^]^ However, selectivity between bacterial and mammalian cells is still a severe issue restricting clinical use. A major contribution to unwanted toxicity of APs can usually be traced back to the hydrophobic component: A surplus of hydrophobicity leads to pronounced hemolysis and other adverse effects, while purely cationic polymers usually lack antimicrobial activity.^[^ ^16^ ^]^

To an extent, compensation with hydrophilic building blocks is possible, as shown by Tew,^[^ ^17^ ^]^ Gillies,^[^ ^18^ ^]^ Kuroda,^[^ ^19^ ^]^ and Boyer.^[^ ^20^ ^]^ However, in these cases the hydrophilic component was a third ingredient within an amphiphilic structure, thus only modulating the amphiphilic balance without additional charge. Hence, the initial dilemma of a trade‐off between activity and host toxicity remains.

In an interesting report from 2014, Yang et al. question if hydrophobicity is strictly necessary and come to the conclusion that also predominantly hydrophilic polymers possess antimicrobial activity.^[^ ^21^ ^]^ However, it is not entirely clear if the used methacrylic polymer backbone still acted as hydrophobic subunit, as this property was not probed. In studies from Chan‐Park and coworkers, antimicrobial properties without a designated hydrophobic building block are described.^[^ ^22^, ^23^, ^24^, ^25^ ^]^ The combination of lysine and glyco subunits is able to kill bacteria without detectable hemotoxicity. Similarly, poly(oxazoline)s, described by Runhui and coworkers, are efficient without strong hydrophobic contribution.^[^ ^26^, ^27^ ^]^ However, a clear correlation with amphiphilicity remains unclear as this property was not probed. In our own contributions *N*‐isopropylacrylamide (NIPAM) was used as building block,^[^ ^28^, ^29^, ^30^ ^]^ whose hydrophobic qualities in such contexts are at least debatable. And while there have been vivid discussions about this topic,^[^ ^31^ ^]^ the general necessity of amphiphilicity was rarely challenged to date.

A common denominator among examples with reduced amphiphilicity was the presence of groups that are able to form hydrogen bonds (sugars, alcohols, and amides). Indeed, it has been reported that the presence of amide groups in amphiphilic polymers improves their bioactivity,^[^ ^32^, ^33^ ^]^ or prearranges APs in solution before membrane interaction.^[^ ^23^ ^]^ However, since the presence of such groups influences the polarity of polymers, isolating the impact of H‐bonds and hydrophobicity from each other is a challenging task.^[^ ^23^, ^32^, ^33^ ^]^ The present study aims to elucidate the impact and necessity of amphiphilicity in the context of biological activity, and also to link this property to hydrogen bonding interactions. We will demonstrate that purely non‐amphiphilic cationic copolymers, lacking nonpolar building blocks are efficient and selective antimicrobials. We will also show that this is the result of supramolecular multivalency—the membrane‐induced clustering of polymers on isolated areas on the membrane induced by hydrogen bonding.

## Results and Discussion

**Figure 1:**
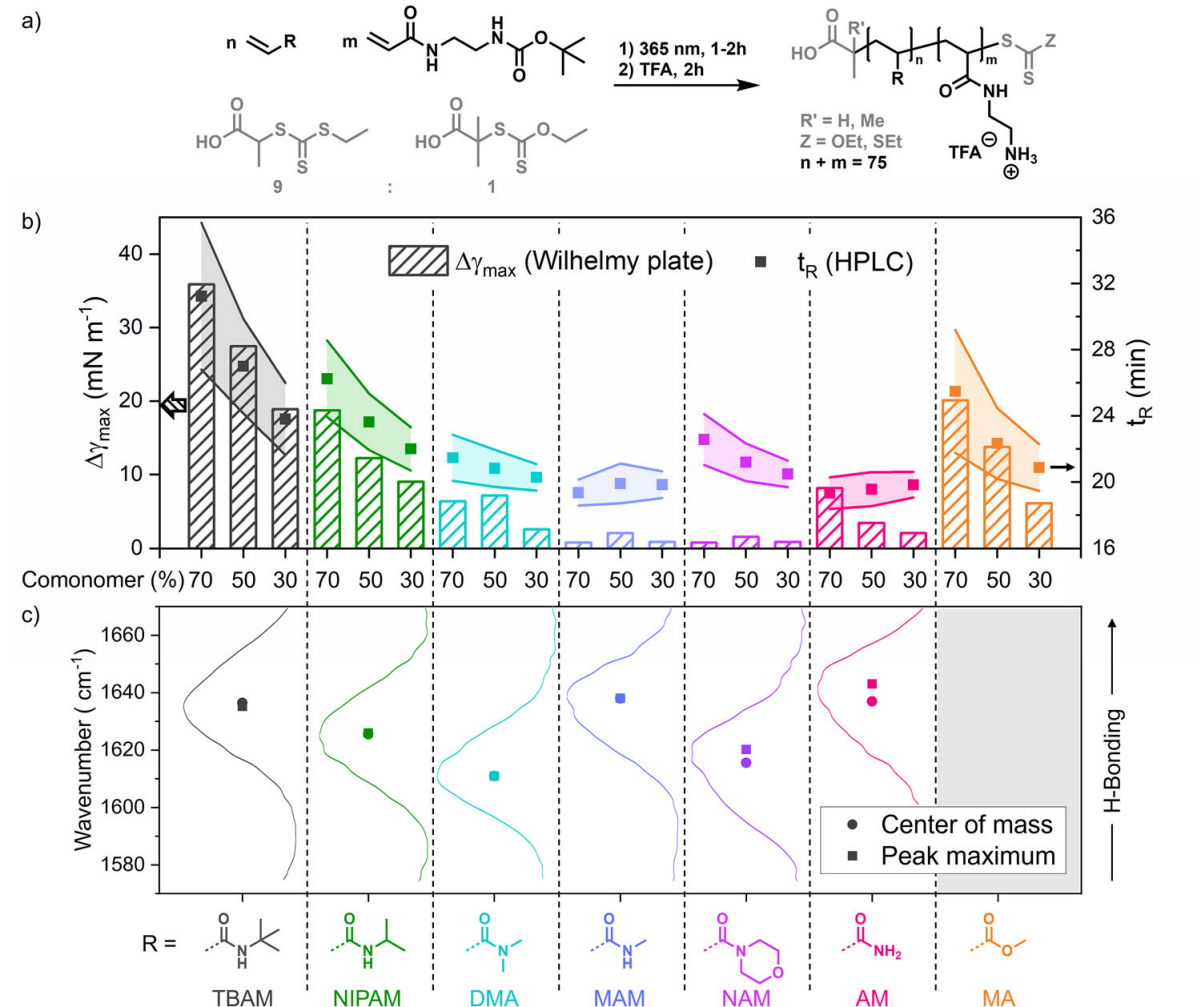
a) Schematic overview of synthesized polymers with varying comonomer composition (30%, 50%, and 70% of noncationic comonomer). A mixture of CTAs (grey) was used for PI‐RAFT polymerization. b) Determination of hydrophobicity and amphiphilicity of polymer via HPLC (scatter) and surface tension (bars) measurements, respectively. HPLC was performed using a water/acetonitrile gradient and retention time (peak maximum) is plotted as scatter plot with error range representing the peak width at half height. Surface tension was determined via a Wilhelmy‐plate and bars represent maximum change in water surface tension upon polymer addition. c) Determination of H‐bonding capacity via FTIR spectroscopy monitoring the C═O stretch vibration (spectra are overlayed with extracted values of peak maximum and center of mass). The method was not applicable to MA copolymers as here the C═O is part of an ester function that shows an intrinsically different wavenumber and thus cannot be compared to amides.

To fathom the impact of polarity and hydrogen bonding on antimicrobial activity, we chose a systematic approach using a copolymer library (Figure 1a). The cationic building block (aminoethyl acrylamide (AEAM)) was maintained in all polymers, whereas the used comonomers were varied, changing apparent polarity and hydrogen bond capacity. While *tert*‐butyl acrylamide (TBAM) and methyl acrylate (MA) form water‐insoluble homopolymers, the other five comonomers were chosen to be more polar. Acrylamides were used preferentially because of their fast polymerization^[^ ^34^ ^]^ and hydrogen bonding qualities. Acrylamide (AM), methyl acrylamide (MAM), NIPAM, and TBAM, all provide hydrogen bond donor units. On the other hand, *N*‐acryloyl morpholine (NAM) and dimethyl acrylamide (DMA) were used, as here the amide is fully substituted. For each combination, the overall charge density was varied, aiming for 70%, 50%, and 30% of comonomer, respectively. A DP of 75 was targeted to aim for molar masses comparable to those of HDPs or their polymeric mimics.

To enable rapid production of materials, xanthate‐supported photo‐iniferter (XPI) reversible addition‐fragmentation chain‐transfer (RAFT) polymerization was used.^[^ ^35^ ^]^ Polymerization was completed in under 2 h (Figures S1–S7) and led to well‐defined polymers (Figures S8 and S9; Table S2), which were deprotected by trifluoroacetic acid (TFA). Molar masses of the final polymers were all in the same size range with polymers with higher AEAM content appearing larger in this SEC setup.

Initially, polymers were probed regarding their hydrophobic/amphiphilic qualities (Figure 1b). HPLC measurements were used to determine the global polarity. The elution follows the expectation based on monomer structure and composition (Figure S10). For most combinations, the retention time (*t*_R_) decreases with increasing charge density. TBAM‐based polymers show high values and NIPAM and MA result in intermediate *t*_R_, whereas the other compositions were more polar. For MAM and AM, the value is unchanged within each series suggesting that used acrylamides are similar in polarity to AEAM. This trend was confirmed by measuring the change in water surface tension as a function of polymer concentration (Figure S11) and plotting the maximum difference in Figure 1b. Obtained values correlate with findings from HPLC measurements (Figure S12), with more polar polymers showing lower impact on interfacial tension. This can be explained by a decreased amphiphilicity (and hence surface activity) of polymers with less hydrophobic comonomers. Thus, we have confirmed that our polymer library spans from very amphiphilic (TBAM‐based) to non‐amphiphilic (MAM, AM‐based) polymers.

While the ability to form H‐bonds can be assessed initially by the molecular structure, we also used FTIR spectroscopy in aqueous solution (D_2_O) to confirm the expected trends. Here, the characteristic C═O stretch vibration of the amide bonds was compared between samples. The frequency of the C═O vibration is influenced by H‐bonding and hence shifts of the peak maximum are a sensitive measure for the presence of water molecules.^[^ ^30^ ^]^ Derivative spectra (subtraction of 70% and 30%, Figure S13) were used to remove spectral contribution of the amine‐containing comonomers. The results follow the expected trend with DMA and NAM showing low and AM and MAM displaying the highest wavenumber (correlating with more intense H‐bonding of the molecular ensemble) in Figure 1c. MA could not be compared with the acrylamides, as its C═O stretch vibration stems from its ester function (rather than the amide bonds in all other polymers), resulting in a different frequency.

**Figure 2:**
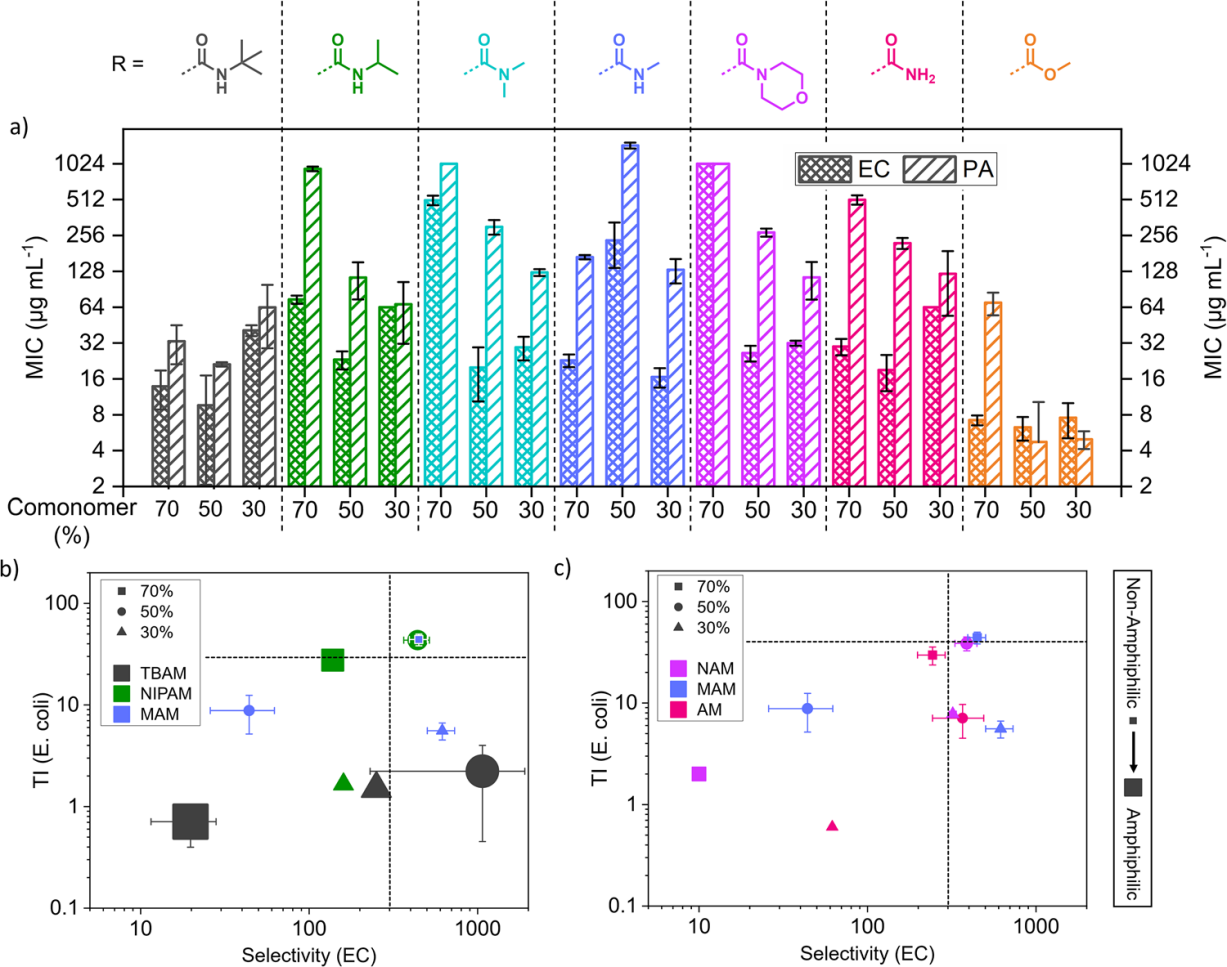
a) MIC_50_ values against EC and PA as function of polymer composition. Performance plots against EC comparing selectivity (based on HC_10_) and TI (based on CC_50_) for polymers with systematically varied amphiphilicity b) or H‐bonding capacity c). The symbol size is linked to the surface activity of polymers with larger sizes indicating higher amphiphilicity of respective macromolecules.

Having established a physicochemical background for the synthesized polymers, we proceeded to apply them in a biological context. Initially, antibacterial properties were probed by determining the minimal inhibitory concentration (MIC) against *Escherichia coli* (EC), *Pseudomonas aeruginosa* (PA) and methicillin‐resistant *Staphylococcus aureus* (MRSA) (Figures 2a and S14–S20). Here it would be expected for polymers with pronounced amphiphilicity and surface activity to be more efficient.

We could not detect activity against gram‐positive MRSA, which could be a result of the thick peptidoglycan layer. However, many of the tested polymers were highly active against gram‐negative bacteria regardless of their polarity/amphiphilicity, resulting in MICs around 16 µg mL^−1^. Less polar macromolecules containing TBAM, NIPAM, and particularly MA performed well, usually with a better performance against EC.

Excitingly, purely hydrophilic polymers containing amide groups capable of H‐bonding (AM and MAM) inhibited the growth of bacteria at concentrations as low as 16 µg mL^−1^. In contrast, hydrophilic polymers with fully substituted amides (NAM, DMA) showed noteworthy activity at higher charge densities. This hints toward a profound importance of hydrogen bond donor moieties. Interestingly, one of the most hydrophilic polymers in the series (MAM70), which shows virtually no surface activity, has strong antimicrobial activity. This finding proves that hydrophobicity apparently is not an essential property in antimicrobial polymers. While noteworthy on its own, this also has important consequences, as the presence of non‐polar units is associated with unspecific toxicity, e.g., toward mammalian cells.^[^ ^16^ ^]^

We proceeded to test compatibility of polymers with red blood cells (hemolytic concentration, HC_10_) and L929 mouse fibroblasts (cytotoxic concentration, CC_50_). Only the most hydrophobic polymer in the library (PTBAM70) showed noteworthy hemolysis, while all others had an HC_10_ above 2000 µg mL^−1^ (Figure S21). Cytotoxicity mainly correlated with charge density, with highly cationic polymers being more toxic (Figure S22). TBAM‐based copolymers were the only exception, where pronounced hydrophobicity and surface activity seem to be more relevant regarding cytotoxicity. Comparing MIC values with HC_10_ and CC_50_, respectively, the selectivity (=HC_10_/MIC) and therapeutic index (TI = CC_50_/MIC) can be determined, with high values representing promising materials.

As shown in the performance plot (Figure 2b), where both values are brought into relation for selected series, amphiphilicity (represented by symbol size) is not a good predictor for bioactivity (Figures S23–S27). If there is a connection, then that strongly amphiphilic polymers (e.g., TBAM‐based) are poorly performing in terms of cytotoxicity and hence TI. For EC, MAM70, as one of the least amphiphilic structures in the study, is among the best performing, with a selectivity above 400 and a TI above 40.

APs with pronounced H‐bonding (based on MAM and AM) are generally more auspicious as they can reach reasonable antimicrobial activity at low content of positive charges. If hydrogen bonding donor sites are blocked (DMA or NAM), higher charge densities are necessary for good antibacterial activity, which is often associated with increased cytotoxicity (Figure 2c).

In addition to experiments on planktonic bacteria, the antimicrobial efficacy of non‐amphiphilic polymer MAM70 was investigated on mature biofilms. Only MRSA‐based biofilm models were available but we still wanted to assess the activity of MAM70 against this model. As biofilms typically exhibit greater resistance to antimicrobial agents,^[^ ^36^ ^]^ and activity against MRSA was poor in MIC tests, a high concentration of 1 mg mL^−1^ was used. Surprisingly, no turbidity was observed in the supernatant of biofilms treated with polymers, indicating successful eradication of planktonic microorganism proliferation (Figure S45). Fluorescence microscopy (Figures S46–S48) revealed a higher proportion of nonviable bacterial cells in the polymer‐treated biofilms relative to negative controls. However, full eradication of biofilms could not be achieved under the used conditions. In addition, an enhanced adhesion and mechanical stability of polymer‐treated biofilms were observed during washing protocols. This could be linked to increased production of extracellular polymeric substances under stress^[^ ^37^, ^38^, ^39^ ^]^ or direct interaction with the polymers.

**Figure 3:**
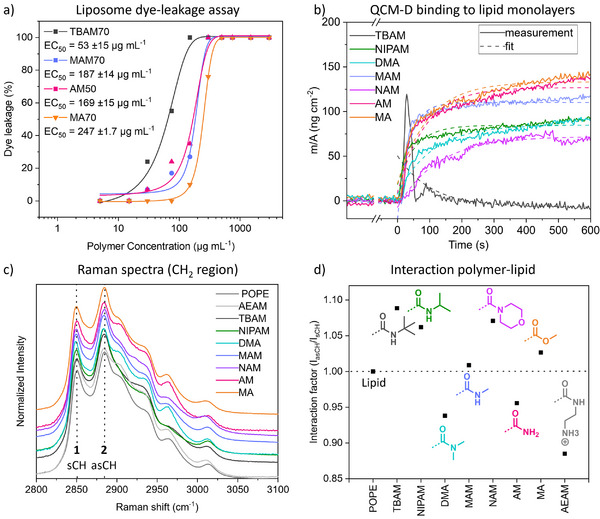
a) Dye leakage study using liposomes from 2‐oleoyl‐1‐palmitoyl‐*sn*‐glycero‐3‐phosphoethanolamine (POPE) and 2‐oleoyl‐1‐palmitoyl‐*sn*‐glycero‐3‐phospho‐rac‐(1‐glycerol) (POPG) (8:2); EC_50_ values are based on Hill1‐fit using Origin software. b) QCM‐D measurements (mass increase) of polymers (all 70% comonomer) on silicon sensors carrying a supported bilayer of 1,2‐dioleoyl‐*sn*‐glycero‐3‐phosphocholine (DOPC) and 1,2‐dioleoyl‐*sn*‐glycero‐3‐phospho‐l‐serine (DOPS) (9:1). Polymers were added at *t* = 0. c) Raman spectra of mixtures of polymers with POPE in a 1:2 mixture. Peaks 1 and 2 are associated with the aliphatic region of the lipid. d) Interaction factor based on the ratio of Raman peaks 2/1 as a function of the respective comonomer.

As we could show that non‐amphiphilic APs perform exceptionally well, we were interested in elucidating their mechanism of action. Classically, successful membrane disruption is connected with a pronounced interaction with the nonpolar fraction of the membrane, which is unlikely for non‐amphiphilic polymers. We probed the underlying mechanism of action using liposomes as membrane mimics. Dye‐leakage assays were used, where membrane disruption can be traced directly via a fluorescence readout.^[^ ^40^ ^]^ We chose to investigate TBAM70, MA70, MAM70, and AM50 due to their good performance (Figure S29). While the former two are expected to lyse lipid membranes via a combination of electrostatic and hydrophobic interaction, the latter two have shown to possess little to no amphiphilicity. However, Figure 3a shows that all polymers are able to permeabilize liposomal membranes in a similar concentration range. While higher than MIC values, the trend of EC_50_ values based on comonomer ratios follows the bioactivity for the acrylamides.

We tested the rate of membrane attachment using a quartz crystal microbalance with dissipation monitoring (QCM‐D) on supported lipid bilayers mimicking biological membranes (Figure 3b). While TBAM70 was apparently sufficiently surface‐active to detach the bilayer, all other samples performed in a similar manner regarding rate and total deposited mass. In particular, the highly similar curves of MA (amphiphilic, no H‐bond donor) and AM (non‐amphiphilic, strong H‐bond donor) illustrate that membrane attachment is mainly dependent on charged density.

Raman scattering measurements of lipids in presence or absence of polymers were performed to evaluate the organization of the bilayer (Figure 3c). The peaks at 2850 cm^−1^ (symmetric CH_2_ stretching) and 2880 cm^−1^ (asymmetric CH_2_ stretching) are sensitive to lipid interaction.^[^ ^41^, ^42^, ^43^ ^]^ A ratio of both signals (*I*_asCH_/*I*_sCH_, after deconvolution, Figures 3d and S34–S38) indicates increased order for values above 1, while a ratio below 1 is characteristic of disordered bilayers. A homopolymer of AEAM was used as a purely cationic control. Interestingly, amphiphilic copolymers, featuring hydrophobic monomers (TBAM, MA, and NIPAM), seem to stabilize lipid structures while non‐amphiphilic APs decrease their order (AM, AEAM, and DMA) or do not influence the peak structure (MAM). While non‐amphiphilic systems presumably only interact with lipid head groups, their impact on the overall membrane structure seems to be disorganizing, which could lead to increased occurrence of defect formation and would explain their antibacterial activity.

**Figure 4:**
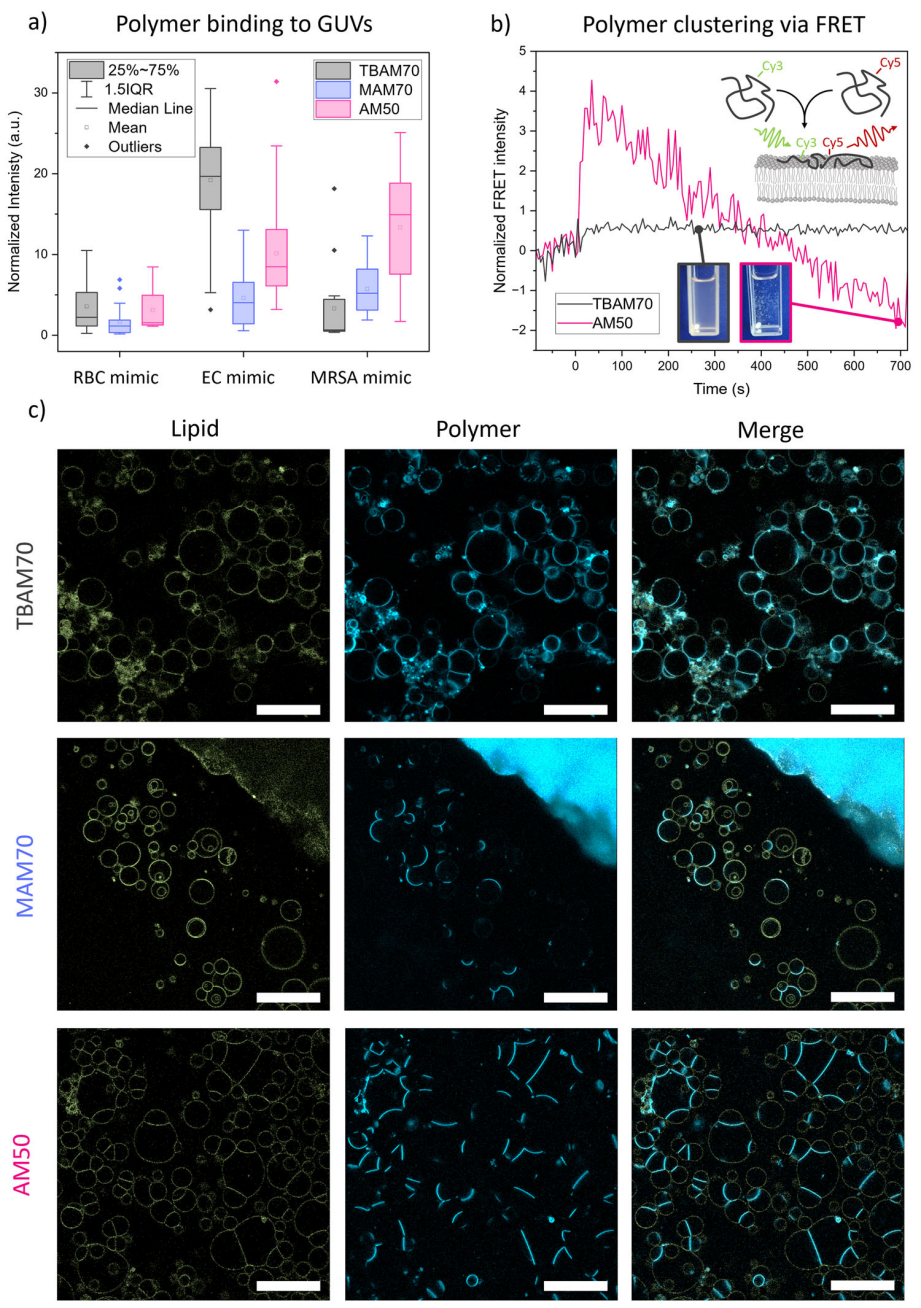
a) Polymer binding to GUVs as quantified by evaluating the total fluorescence intensity emitted by polymer molecules colocalized with lipid membranes in each image, normalized by the amount of pixels belonging to GUVs (see Methods for details, total data amount of points: 157), b) FRET intensity of polymers in contact with bi‐lipid membrane (EC mimic) in the form of liposomes as a function of time, c) microscopic images of labeled GUVs mimicking EC membranes and labeled polymers. Images were contrast enhanced to increase visibility; scale bars are 50 µm.

To gain deeper insight into the polymer–membrane interaction, giant unilamellar vesicles (GUVs) were monitored in the presence of APs via fluorescence microscopy. Polymers and membranes were fluorescently labeled, and polymer binding was quantified by colocalization of both signals within microscopic images depending on the membrane composition (Figures 4a, S42, and S43). Red blood cell (RBC) mimics show low binding for all polymers, while bacteria‐mimicking membranes show a stronger interaction. Interestingly, TBAM70 binding to EC mimics is much stronger than to MRSA mimics, which coincides with the biological activity. MAM70 and AM50 show binding on both bacterial mimics with AM50 being more pronounced, which could be a result of the increased charge density of this polymer compared to the other samples.

In addition to binding, a difference in the spatial distribution of bound polymers was observed. For amphiphilic TBAM70, the polymer evenly binds to the available interface. In contrast, non‐amphiphilic polymers cluster in certain areas (Figure 4c). This effect can also be observed in binding kinetics of MAM (Video S1). Moreover, presence of non‐amphiphilic APs seems to promote vesicle fusion, as apparent from the reduced curvature between GUVs. In Video S1, the transition from isolated to partially fused vesicles can even be directly observed during polymer addition. While leaky fusion can be a false indicator of membrane activity, as it can be prevented in bacteria via, e.g., the cell wall,^[^ ^44^ ^]^ in the present case it seems to be part of a productive mechanism regarding antimicrobial action. Still, this aspect could explain low activity against MRSA, as in gram‐positive bacteria, the protective peptidoglycan layer is thicker, and no accessible outer membrane is present.

This unexpected difference in the mode of interaction between polymer and membrane could be caused by the combination of hydrogen bonding ability and non‐amphiphilicity. While amphiphilic polymers are firmly lodged in the membrane by their non‐polar side groups, non‐amphiphilic polymers lacking these interactions are likely more mobile. Consequently, attractive interaction between polymers via H‐bonding can then lead to clustering at certain regions of the membrane. Interestingly, neither amphiphilic nor non‐amphiphilic polymers self‐assemble in PBS buffer solution (Figure S39), which is likely associated to charge repulsion of the poly(cations). Only in contact with a negatively charged membrane, when charges are screened by the lipid headgroups, clustering is possible. To support this hypothesis, evaluations on liposomes were conducted via Förster resonance energy transfer (FRET). Polymers were labeled with the FRET donor dye cyanine 3 (Cy3) or with the FRET acceptor dye cyanine 5 (Cy5). Proximity of the dyes can be probed by exciting the donor and detecting the emission of the acceptor dye. The respective FRET ratio gives an indication of dye proximity as energy transfer is limited to a distance below 10 nm.^[^ ^45^ ^]^ When polymers are mixed in aqueous solution only low FRET ratios can be detected (Figures S40 and S41). However, once liposomes are added, the ratio increases (Figure 4b). This increase (relative to the amount of dye present) is much more drastic for AM when compared to TBAM, supporting a clustering effect of polymers on the interface that only proceeds in the absence of hydrophobic interactions. Shortly after, the FRET intensity decreases again for non‐amphiphilic AM while it remains constant for TBAM. This is associated with a precipitation of liposomes in the former case as here polymer clustering leads to the formation of larger aggregates. For TBAM70, the mixture remains colloidally stable. It should be noted that the fluorescence of the lipids in GUV experiments remains homogeneously distributed. As such, a phase separation and domain formation of different lipids, as observed for amphiphilic APs^[^ ^46^ ^]^ can be excluded as cause for these findings.

**Figure 5:**
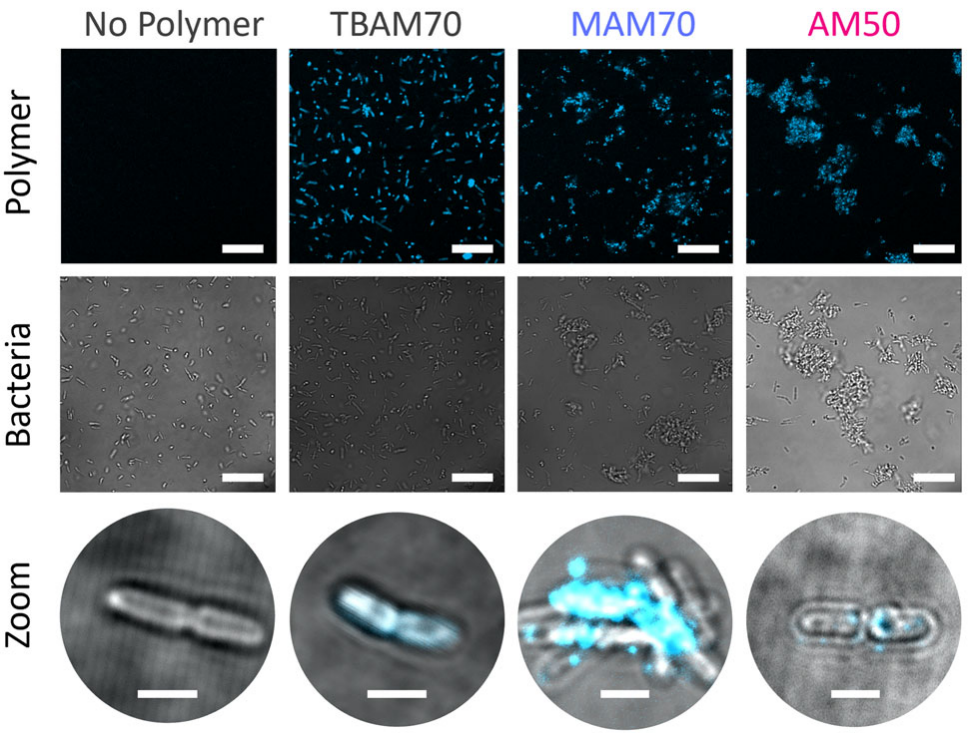
Confocal microscopy images of bacteria treated with dye‐labeled copolymers. Bacteria were incubated with either PBS (“No Polymer”) or a 20 nM solution of Alexa405‐labeled polymer for 1 h, shortly centrifuged and resuspended in Luria/Bertani medium before imaging. Zoomed images (bottom row) were acquired in samples additionally containing 0.5% agarose to minimize the lateral movements of the bacteria. All images were acquired at room temperature (23 ± 1 °C). Scale bars are 20 µm (2 µm for zoomed pictures).

Based on these results, we were interested in visualizing the interaction of polymers with living bacteria. Using labeled polymers, we proceeded to treat EC, monitoring the presence of copolymers on the bacterial cell envelope (Figure 5).

Indeed, after treatment with amphiphilic polymer TBAM70, individual cells remain isolated. The presence of non‐amphiphilic AP MAM70 induces aggregation, which is even more pronounced for the AM‐based polymer. Moreover, images with a higher magnification reveal that non‐amphiphilic polymers cluster on the bacterial membrane in a similar way that was found using GUVs as models.

**Scheme 1:**
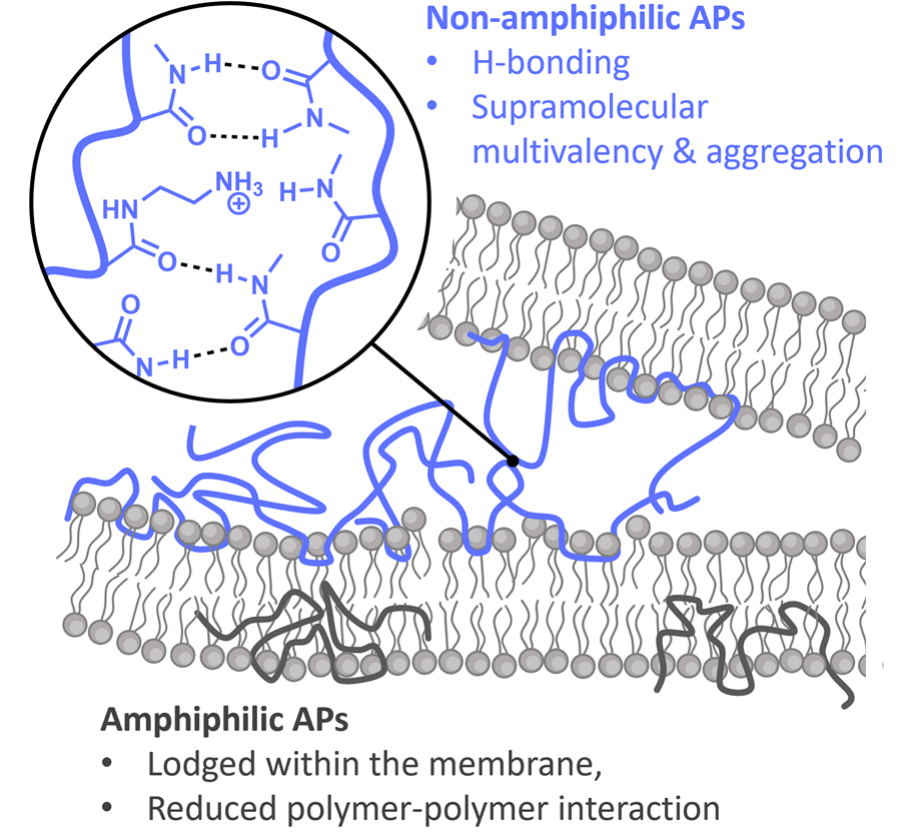
Sketch of differences in membrane interaction between amphiphilic and non‐amphiphilic APs.

Based on these findings, we hypothesize that the mechanism of action of non‐amphiphilic APs is based on a combination of membrane interaction and polymer–polymer interaction that leads to disorder in the bilayer and membrane permeabilization, as well as aggregation and potentially fusion of bacteria. Moreover, the recruitment of polymers from solution, as facilitated by immobilized polymers leads to an enrichment of APs at certain parts of the bacterial cell envelope. We have demonstrated in previous studies that multivalent presentation of antimicrobial subunits enhances their activity drastically.^[^ ^28^, ^29^, ^30^, ^47^ ^]^ In the present case, we expect a supramolecular multivalency based on H‐bonding interactions between surface‐bound polymers, thus enhancing local membrane activity (Scheme 1). If this hypothesis is true, a combination of all three components (polar, nonpolar, and cationic) should not lead to a significant improvement due to interference between competing physical processes: the presence of hydrophobicity locks the polymers in the membrane and prevents clustering, while polar units reduce interaction with the hydrophobic membrane domain. Indeed, a copolymer of MA, MAM, and AEAM (in equal molar ratios) does not show increased antimicrobial activity (MIC_50_: EC = 50 µg mL^−1^; PA > 1024 µg mL^−1^) indicating that a combination of both effects is not productive (Figure S42).

## Conclusion

In summary, we clearly demonstrate that amphiphilicity is not an essential quality in APs. A combination of cationic building blocks with hydrogen‐bonding motives enables pronounced antibacterial activity while the absence of amphiphilicity leads to strongly reduced unspecific toxicity. While still membrane permeabilizing, their mode of interaction differs from conventional APs. Most strikingly, non‐amphiphilic polymers agglomerate at isolated regions of the membrane. This supramolecular multivalency is not observed for classical APs and is only possible in the absence of hydrophobicity. These findings open new possibilities for the design of antimicrobial substances to solve the crisis of antimicrobial resistance.

## Supporting Information

The authors have cited additional references within the Supporting Information.^[^ ^13^, ^48^, ^49^, ^50^ ^]^

## Conflict of Interests

The authors declare no conflict of interest.

## Supporting information



Supporting Information

Supporting Information

## Data Availability

The data that support the findings of this study are available from the corresponding author upon reasonable request.
