# Supplementary material for: Non‐Amphiphilic Antimicrobial Polymers
Source: Angew Chem Int Ed Engl. 2025 Jul 7;64(33):e202507564. doi: 10.1002/anie.202507564 (PMC12338418; doi:10.1002/anie.202507564)
Supplement: Supplementary file 1 — Supporting Information [file ANIE-64-e202507564-s001.pdf]

SUPPORTING INFORMATION

Non-Amphiphilic Antimicrobial Polymers

Alain M. Bapolisi,[a,±] Anne-Catherine Lehnen,[a,b,±] Martin Wolff,[c] Jana Kramer,[c] Sergio Kogikoski Jr.,[a] René Steinbrecher,[a] Nicole Michler,[d] Andreas Kiesow,[d] Ilko Bald,[a] Martina Obry,[e] Sebastian Kersting,[e] Till Stensitzki,[a] Henrike M. Müller-Werkmeister,[a] Meike N. Leiske,[f,g] Salvatore Chiantia,[c] Matthias Hartlieb[a,b]*

Affiliations

a) Institute of Chemistry, University of Potsdam, Karl-Liebknecht-Straße 24-25, 14476, Potsdam, Germany
b) Fraunhofer Institute for Applied Polymer Research (IAP), Geiselbergstraße 69, 14476, Potsdam, Germany
c) Institute of Physical Biochemistry, University of Potsdam, Karl-Liebknecht-Straße 24-25, 14476, Potsdam, Germany
d) Fraunhofer-Institut für Mikrostruktur von Werkstoffen und Systemen IMWS, Walter-Hülse-Str. 1, 06120 Halle (Saale)
e) Fraunhofer Institute for Cell Therapy and Immunology, Branch Bioanalytics and Bioprocesses (IZI-BB), Am Mühlenberg 13, 14476 Potsdam
f) Faculty of Biology Chemistry & Earth Sciences, University of Bayreuth, Universitätsstraße 30, 95447 Bayreuth, Germany
g) Bavarian Polymer Institute, Universitätsstraße 30, 95447 Bayreuth, Germany

± Authors have contributed equally to the performed work.

* Correspondence to mhartlieb@uni-potsdam.de

Materials and Methods

The chemicals used in this study were purchased from Sigma-Aldrich, Alfa Aesar, TCI (Tokio Chemical Industry), Acros Organics, Merck, Carl Roth, Lumiprobe and Fisher Chemicals. Inhibitor from monomers were removed via a short aluminum oxide column (*N*-acryloyl morpholine (NAM), dimethyl acrylamide (DMA), methyl acrylate (MA)) or via recrystallization (*N*-isopropyl acrylamide (NIPAM)) prior to polymerization. Propionic acid ethyl trithio-carbonate (PAETC),[1] and 2-(Ethoxycarbonothioylthio)-2-methylpropanoic acid,[2] as well as *N*-tertbutoxycarbonyl-amino ethyl acrylamide (BocAEAM)[3] were synthesized according to literature procedures. Acryloyl chloride (Merck Millipore, > 96.0 %, stabilised with phenothiazine) was distilled to remove the inhibitor.

Nuclear magnetic resonance (NMR) spectroscopy

^{1}H-NMR spectra were recorded on a Bruker AVANCE NEO 400 MHz spectrometer in DMSO-D_6 or D_2O. Chemical shift values are reported in ppm, and the residual proton signal of the solvent was used as the internal standard.

Size exclusion chromatography (SEC)

SEC with simultaneous UV and RI detection was performed with THF as the eluent at a flow rate of 0.5 mL min^{-1} at room temperature; the stationary phase was a 300 × 8 mm^2 PSS SDV linear M column. Solutions containing polymer samples were filtered through 0.45 µm polytetrafluoroethylene filters; the injected volume was 100 µL. Polystyrene (PS) standards (PSS, Mainz, Germany) were used for calibration. Aqueous SEC measurements with simultaneous UV and RI detection were performed with water containing 0.1 M NaCl and 0.3 V% formic acid as eluent at a flow rate of 1 mL min^{-1} at 40 °C; stationary phase was 300 × 8 mm^2 PSS NOVEMA Max column and using a poly(ethylene glycol) calibration (PSS, Mainz, Germany).

Dynamic light scattering (DLS)

Dynamic light scattering (DLS) was measured on a Zetasizer Ultra (Malvern Panalytical, United Kingdom) at a measurement angle of 173° at 25°C. 1 mL of polymer solution in phosphate buffered saline (PBS 1X, pH 7.4) at a concentration of 1 mg mL^{-1} was placed in a disposable 4 mL polystyrene cuvette.

Photoreactor

For photo-polymerizations a PhotoCube from ThalesNano was used. Reactions were cooled via cooling water maintaining a temperature of 20 °C. In this work only λ = 365 nm light was used at an intensity

of 100% (setting high). The LED intensity was measured at the sample position with a commercial S170C power meter (Thorlabs). It should be noted that the flat sensor only fits into the sample chamber vertically while the reaction chamber is illuminated from all four sides. Hence, ¼ of the LEDs are behind the sensor. This was adjusted by setting the measured intensity as ¾ of the total intensity which is 75 mW cm^{-2}.

High performance liquid chromatography (HPLC) measurements

HPLC was performed on a Shimadzu instrument (SCL-10Avp) equipped with a degasser (DGU-14A), mixer (FCV-10ALvp) and pump (LC-10ATvp). The flow rate was set to 1 ml min^{-1} and samples with a concentration of 5 g L^{-1} polymer were injected using an autosampler (SIL-20ACht) with an injection volume of 25 µl. For investigation of the polymer hydrophobicity the instrument was fitted with a Pursuit XRs 5 C18 250 × 4 mm column (Agilent). As mobile phase A water with 0.04% trifluoroacetic acid and as mobile Phase B acetonitrile with 0.04% trifluoroacetic acid was used. All mobile phase solvents were HPLC grade. HPLC was performed at room temperature starting with 1% mobile phase B for 10 minutes followed by a gradient up to 95% mobile phase B over 35 minutes. The retention of the polymer was monitored at λ = 220 and 309 nm simultaneously using a UV detector (SPD-10Avp).

Surface tension measurements

A Kibron equipment (Helsinki, Finland) was used to measure the surface tension. The surface activity of polymers was monitored by recording the surface tension while increasing the concentration of polymers. A Kibron Wilhelmy plate connected to the sensor was cleaned successively with ethanol, acetone and water and then flamed before each experiment. The surface tension of the cleaned plate sensor was calibrated first in air and then at the air-water interface in a clean Dynecup containing 3.2 mL of phosphate buffered saline (PBS 1X) used as subphase. Polymer stock solutions in PBS were gradually added to the subphase and left to equilibrate for about 100 seconds before the next polymer injection and the surface tension was recorded and plotted against the respective final polymer concentrations (from 0.001 µg mL^{-1} to 100 µg mL^{-1}) in the Dynecup using Origin Hill fitting tools.

Fourier-transform infrared spectroscopy (FTIR)

All FTIR spectra were measured on a Bruker Tensor II spectrometer. For each spectrum, we took 128 scans with a spectral resolution of 1 cm^{-1} directly against a solvent background. The sample (10 mg ml^{-1} in D_2O) was placed between two CaF_2 windows separated by a 50 µm telfon spacer. To extract the

spectra of each copolymer, we measured polymers with different ratios (30%, 50%, 75%) of the two building blocks. The process is visualized in Figure S13: After subtraction of a linear baseline, we normalized all spectra to the peak at 1674 cm^{-1}, which we previously assigned to C=O vibration of the common NIPAM building block. We then subtracted the spectra of highest ratio with the lowest ratio to extract the spectrum of the other copolymer. While the interaction between the the two building blocks may affected the spectra as well, we observe that the subtraction from the spectra of the middle ratio from lowest yields very similar spectra, showing us that the influence of such interactions must be comparatively small at the given ratios.

Raman spectroscopy

Polymers solutions and 2-Oleoyl-1-palmitoyl-sn-glycero-3-phosphoethanolamine (POPE) lipid aqueous dispersion were mixed in a (mass) concentration ratio of 1:2 and incubated for at least 10 min at room temperature and then spotted on a clean silicon (Si) wafer. The solvent was air-dried and samples measured with Raman spectroscopy.

The Raman spectra were collected using a LabRam HR Evolution from HORIBA SAS, France. The samples were measured using the microscope option with a 561 nm laser beam focused using a 50X long-distance objective (Olympus). The spectra were collected over the course of 1 h using 2 s of integration time; the spectra obtained were the sum of all the collected signals over the 1 h. The laser power was kept constant at about 9 mW. The obtained spectra were analyzed using the software LabSpec 6.0 from HORIBA Scientific and Origin 2023. The signal from the Si substrate was subtracted from the collected spectra to remove the possible overlap between the Si vibrations from the obtained spectra. Nevertheless, the areas of the spectra analyzed are located in regions where Si does not possess strong vibrational modes.

The samples were prepared freshly for the Raman measurement. To emulate the interaction of the antimicrobial polymer with the cell membrane, a mixture of 1:1 (m:m) of POPE and polymer was used. In this concentration, we could detect signal differences in the Raman spectra of POPE related to the presence of polymers. The samples were prepared in solution and then drop-casted of a clean silicon chip. The mixture was allowed to dry at RT. The polymer AM70 was also measured to check if there would be an overlap of bands on the lipid polymer mixture, which could hinder the evidence of intermolecular interactions between the species.

Some regions of the obtained Raman spectra were deconvoluted using the Peak Analyzer option in Origin 2023 software. Each vibrational mode was deconvoluted using Lorentzian functions. The number

of applied functions was chosen depending on the spectra and using the literature to reference the expected number of possible vibrations in the selected area.

Experimental Procedures

Synthesis of methyl acrylamide (MAM)

In a 1 L Erlenmeyer flask 10.0 g (148.11 mmol, 1.00 eq.) methyl ammonium chloride is dissolved in 40 mL distilled water and mixed with 370 mL DCM and stirred vigorously. Following 12.74 g (318.44 mmol, 2.15 eq.) NaOH dissolved in 80 mL distilled water is added and the mixture is cooled down with an ice bath. Acryloyl chloride (13.81 g, 152.56 mmol, 1.03 eq.), dissolved in 130 mL DCM, is added dropwise over 30 min. The reaction mixture is warmed to room temperature (RT) and stirred overnight. The phases are separated, the aqueous phase extracted with DCM (4 times with 40 mL). The united organic phase is dried over $MgSO_4$ and the solvent removed under reduced pressure. MAM is received as a colourless liquid (9.85 g, 78 %).^{1}H NMR (400 MHz, $CDCl_3$, 298 K): δ = 6.69 (1H, br, NH), 6.25 - 6.02 (2H, m, =CH (*E*-position*)* + =CH-), 5.59 (1H, m, =CH (*Z*-position)), 2.85 (3H, d, CH_3, J_3= 4.80 Hz) ppm. ^{13}C NMR (400 MHz, $CDCl_3$, 298 K): δ = 166.65, 130.92, 125.84, 26.26 ppm.

General polymerization procedure

Polymerization reactions were performed in 5 mL pyrex vials without stirring. A mixture of PAETC and Xan (9:1) was dissolved in a mixture of dioxane and water (8:2) and monomers were added to yield a total monomer concentration of 1 mol L^{-1} with a monomer/CTA ratio of 75. The solution was capped using a rubber septum and oxygen was removed by purging with nitrogen gas via a needle for 10 min prior to polymerization. Subsequently, samples were irradiated in a photo reactor (PhotoCube from ThalesNano) using a wavelength of 365 nm at 100% intensity (setting high).

Polymerization kinetics

Kinetics of copolymerization reactions were performed using a comonomer ratio of 1:1 using above mentioned conditions. Samples were drawn at predefined reaction times (0, 1, 2.5, 5, 10, 20, 30, 60 min). For this, irradiation was ceased and continued after the samples were withdrawn. Conversion of each monomer was probed via ^{1}H-NMR spectroscopy and the molar mass distribution was investigated via SEC in THF.

Polymerization reactions

Copolymers were produced using above mentioned conditions, a total reaction volume of 3.5 mL and an irradiation time of 1 h, using different pairs of monomers. BocAEAM was mixed with the respective comonomers in different ratios yielding polymer with 30%, 50% and 70% of comonomer. As conversions were very high, solvent was removed from the reaction mixture via freeze drying to obtain the protected polymer as a off-white powder.

Deprotection

To remove the Boc protection group the polymer was dissolved in 2 mL of trifluoro acetic acid (TFA) in a 50 mL round bottom flask and stirred for 3 h at 40 °C. The polymer was precipitated in cold diethyl ether and separated via centrifugation. After that the polymer was dissolved in EtOH and precipitated again in diethyl ether. After drying polymers were obtained as off-white powders.

Table S1: Composition of reaction mixtures for each copolymer. For each polymerization 8.5 mg (0.042 mmol) of PAETC and 1 mg (0.0047 mmol) MeXan were used.

Sample	(Boc)AEAM		Comonomer		Dioxane/water (8:2)	Conversion[a]
	m (mg)	n (mmol)	m (mg)	n (mmol)	(µL)	%
TBAM 70	225	1.05	312	2.45	2964	98.5
TBAM 50	375	1.75	223	1.75	2903	98.2
TBAM 30	524	2.45	134	1.05	2842	98.7
NIPAM 70	225	1.05	277	2.45	2998	97.3
NIPAM 50	375	1.75	198	1.75	2927	97.1
NIPAM 30	524	2.45	119	1.05	2857	98.1
DMAM 70	225	1.05	252	2.45	3023	95.0
DMAM 50	375	1.75	180	1.75	2946	97.5
DMAM 30	524	2.45	104	1.05	2868	98.7
MAM 70	225	1.05	209	2.45	3064	96.3
MAM 50	375	1.75	149	1.75	2975	97.3
MAM 30	524	2.45	89	1.05	2885	97.0
NAM 70	225	1.05	346	2.45	2967	98.7
NAM 50	375	1.75	247	1.75	2905	98.3
NAM 30	524	2.45	148	1.05	2844	98.4
AM 70	225	1.05	174	2.45	3101	98.1
AM 50	375	1.75	124	1.75	3001	82.1
AM 30	524	2.45	75	1.05	2901	86.0
MA 70	225	1.05	181	2.45	2674	90.0
MA 50	375	1.75	151	1.75	2967	95.5
MA 30	524	2.45	90	1.05	2881	97.0

a) Determined by ^{1}H-NMR spectroscopy (400 MHz, DMSO-D_6)

General procedure for polymer labeling

Free amino groups of deprotected polymers of interest were labelled with either Alexa Fluor™ 405 NHS Eester (succinimidyl ester) (Alexa405), Cyanine3 NHS ester (cy3) or Cyanine5 NHS ester (cy5). Briefly, polymers were dissolved (5 mg mL^{-1}) in a mixture of 20 parts PBS 1X and 1 part of 0.2 M sodium

bicarbonate (adjusted to pH 7.9), necessary to ensure complete dissolution of the polymers. Required volume of dye stock solutions (in DMSO) to achieve targeted degree of labelling of 2 amino group per polymer molecule was added to the polymers' solution and left to incubate for 1 hour under stirring and room temperature conditions. Alexa405-labelled polymers were purified from free dye chromatographically over Cytiva Sephadex G-25 Fine column material (packed in a 10/300 column body) conditioned with 10 mM phosphate buffer with 10 mM sodium chloride. Dialysis bags (MWCO 3500) were used to purify cy3/cy5-labelled polymers by dialysis against PBS buffer. After purification, labelled polymers were stored at 4°C. A quantification of the labelling efficiency was not possible as a direct measure of the polymer concentration in UV Vis was prevented by the dye absorption.

Determination of MIC values

To determine the minimum inhibitory concentration (MIC) the gram-negative bacterium *Escherichia coli* (*E. coli*, ATCC 25922) and *Pseudomonas Aeruginosa* (*P. aeruginosa* (ATCC 10145) and the gram-positive bacterium methicillin-resistant *Staphylococcus aureus* (MRSA; ATCC 43300) were used. To produce a cell culture of respective bacteria strain a single colony of bacteria was inoculated in Mueller-Hinton broth medium (MHB, 5 mL) and incubated over night at 37 °C. By measuring the optical density at 600 nm (OD_{600}) the concentration of cells in solution was assessed and decreased to an OD_{600} of 0.1 via dilution with MHB medium. For application suspension was diluted with medium in a ratio of 1:5000 to achieve the final bacteria concentration. Next to this also the polymeric samples were dissolved in MHB and a serial dilution was performed in a 96-well plate (three determinations of each concentration; with wells containing 100 µL of respective sample solution; range of concentration was 1024 µg mL^{-1} to 2 µg mL^{-1}). 100 µL of bacterial suspension was added to each well and then incubated for 22 h at 37 °C. The growth of bacteria in vitro was evaluated via measurements of OD_{600} and normalized using positive and negative control. As negative control wells containing MHB medium were used and wells with medium (100 µL) and bacteria suspension (100 µL) served as positive control. The MIC_{50} was calculated using a dose response function of Origin software.

Evaluation of hemotoxicity

To determine hemocompatibility red blood cells (RBCs) from defibrinated sheep blood was used. RBCs were isolated by centrifugation (4500 rpm for 1 min) and washed two times via centrifugation with PBS which served as medium. The washed PBS-RBC-suspension was diluted with PBS in a ratio of 1: 15. This suspension was used for the determination of hemolytic concentration. Separately APs were dissolved in medium and a serial dilution was performed in a 96-well plate (three determinations for each

concentration; each well contains 100 µL of polymer solution; range of concentration was 10240 µg mL $^{-1}$ to 40 µg mL $^{-1}$). Accordingly, 100 µL of cell suspension was added to each well and incubated at 37 °C for 1 h. To separate erythrocytes from suspension, the well plate was centrifugated for 5 min at 1000 g. 100 µL of supernatant of each well was transferred to another 96-well plate and the amount of lysed cells was analyzed by measuring absorption at 544 nm. Results were normalized using negative and positive control. Wells containing RBCs and PBS served as negative control and as positive control wells containing RBCs and Triton X solution (1 % in PBS) were used. Hemolysis concentration (HC_{10}) was calculated using a dose response function or a Hill1 fit of Origin software.

Evaluation of cytotoxicity

L929 cells were grown in Dulbecco's Modified Eagle Medium (DMEM, VWR) supplemented with 10% (v/v) fetal bovine serum (FBS, VWR), 100 U mL $^{-1}$ penicillin (VWR), and 100 µg mL $^{-1}$ streptomycin (VWR). Cells were maintained at 37 °C in a fully humidified atmosphere containing 5% CO_2.

To determine cell viability, cells (10^4 per well) were seeded in 96-well plates and allowed to adhere overnight. The medium was subsequently removed and replaced by fresh, polymer-containing medium. Then, the cells were incubated at 37 °C for an additional 24 h. After that, the medium was removed, and the cells were washed with 100 µL Dulbecco's phosphate buffered saline (DPBS, VWR). Next, fresh media containing the thiazolyl blue tetrazolium bromide (MTT) (concentration: 1 mg mL $^{-1}$) was added (100 µL per well). Note: MTT (50 mg) was dissolved in 10 mL of sterile DPBS filtrated (membrane, 0.22 µm), and 1 to 5 diluted in culture medium prior to use in this assay. After incubation at 37 °C for 3 h, 100 µL of DMSO was added to each well and the plates were gently shaken in the dark for 15 min to dissolve the formazan crystals. Quantification was done by measuring the absorbance at λ = 580 nm using a microplate reader. Untreated cells on the same plate served as negative control (100% viability), cells treated with 20% DMSO as positive control (0% viability), and wells without cells as background. Experiments were performed in triplicates on three different plates. The relative cell viability was determined by the following equation.

$$\%Cell\ viability = \frac{Abs.sample - Abs.background}{Abs.negative\ control - Abs.background} \times 100$$

Preparation of large unilamellar liposomes

Large unilamellar vesicles (LUVs) mimicking RBC membranes were prepared from a mixture of 1,2-dioleoyl-sn-glycero-3-phosphocholine [DOPC] (9 mg, 12.3 µmol) and 1,2-dioleoyl-sn-glycero-3-phospho-L-serine (sodium salt) [DOPS] (1 mg, 1.23 µmol). For LUVs mimicking *E. coli* the lipid mixture was composed of 2-Oleoyl-1-palmitoyl-sn-glycero-3-phosphoethanolamine [POPE] (8mg, 11.2 µmol) and 2-Oleoyl-1-palmitoyl-sn-glycero-3-phospho-rac-(1-glycerol) sodium salt [POPG] (2 mg, 2.6 µmol). For each batch formulation, phospholipids mixtures were dissolved in $CHCl_3$ (1 mL) in a 25 mL round bottom flask. A thin lipid film was formed by drying the organic solvent using a Rotavapor under vacuum. The dried thin film was then hydrated with PBS (1ml) by continuous stirring for 1 hour at room temperature to form liposomes. An extruder was used to control the size of the formed LUVs by passing them through a 400 nm polycarbonate filter membrane (15 times). For QCM-D measurements, a subsequent extrusion through a 100 nm filter was performed on DOPC:DOPS LUVs. Liposomes suspensions were stored at 4 °C and used within one week.

For dye leakage experiments, LUVs mimicking membranes of *E. coli* were also prepared by thin film hydration method as described above. The formed thin film was particularly hydrated with dye-calcein solution (1 mL). The calcein solution (40 mM) was prepared by dispersing calcein powder (249 mg, 0.3 mmol) in 8 mL of Na_2HPO_4 buffer (10 mM) at pH 7.4, and a solution of NaOH (1 M) was added dropwise to completely dissolve calcein and then the pH of calcein solution was re-adjusted to 7.4 using HCl and finally the volume was topped up to 10 mL. To improve loading of calcein in the vesicles, the suspension was subjected to 5 freeze-thawing cycles in a round bottom flask after hydration; freezing for 5 min in liquid nitrogen followed by thawing for 15 min in a water bath at room temperature. To control the size of the liposomes, the vesicles were extruded 15 times through 400 nm polycarbonate membrane. To purify the calcein-loaded liposomes from the non-encapsulated dye, the liposomes suspensions were diluted with PBS buffer then concentrated by centrifugation at 4000 rpm using an Amicon Ultra - 15, PLTK Ultracel-PL Membran, 30 kDa overnight at 4 °C. The purification process was repeated at least 2 times. The concentrated POPE:POPG liposomes loaded with calcein were stored at 4 °C and used within one week.

Dye-leakage experiments

A Fluoromax 4 spectrofluorometer (Horiba, USA) was used for kinetics acquisition measurements of polymer-induced dye leakage from the calcein loaded liposomes made of POPE:POPG (8:2) lipids. The excitation wavelength was set at 490 nm (slit: 1.0 nm Bandpass) and the fluorescence intensity was monitored over time (800 seconds) at the emission wavelength of 525 nm (slit: 1.0 nm Bandpass). In a quartz cuvette, 2 mL of LUVs loaded with calcein (diluted 40 times in PBS) was placed for fluorescence

monitoring under stirring at room temperature. A baseline of calcein fluorescence before polymer addition was normalized for each sample (0% dye leakage). Then 20 µL of polymer solutions (in PBS 1X) of various concentrations were added to the cuvette 100 seconds after the start of the run and the increment of fluorescence induced by polymers was recorded. After 700 seconds, which were necessary to achieve complete activity of the polymer on the liposomes, 20 µL of Triton X (20%) were added as positive control to completely disrupt the liposomes and therefore to determine the fluorescence intensity corresponding to 100% dye leakage.

The measured fluorescence intensity was normalized to percentage leakage activity, *Y,* using the following equation:

$$Y = \frac{I_t - I_0}{I_\infty - I_0} \times 100$$

I_o represent the fluorescence intensity I_t before the addition of the polymer samples, and I_∞ is the maximum fluorescence I_t after the addition of Triton-X. To determine the 50% polymer-induced dye leakage, EC_{50}, the final polymer induced leakage percentage just before the addition of Triton-X was plotted versus the final polymer concentration by Hill1-fit using Origin 2023 software.

Förster resonance energy transfer (FRET) experiments

A Fluoromax 4 (Horiba, USA) equipment was used for FRET assays by fluorescence time-based acquisition performed at the maximum emission wavelength of the acceptor cyanine dye cy5 (Em_{max} = 657 nm) upon maximum excitation a of the donor cyanine dye cy3 (Exc_{max} = 555 nm). Briefly, previously labelled polymers, cy3-labbeled polymers and cy5-labelled polymers, were respectively used as donors and acceptors, and LUVs mimicking *E. coli* membrane composed of POPE:POPG (8:2) were used as liposome models. Full emission spectra of labelled polymers (cy3 and cy5- labelled polymers) individually and their mixtures without LUVs were recorded in PBS. For FRET experiments, the change in fluorescence emission was recorded from a 2 mL liposomes suspension (0.25 mg mL^{-1}) placed in a quartz cuvette to have a baseline emission of LUVs. After 100 seconds, 40 µL of freshly premixed cy3-labelled polymer and cy5-labelled polymer at equivalent concentration (5 mg mL^{-1}) was added to LUVs. Polymer addition was considered as time zero and changes in the emission signal were monitored for at least 1000 seconds. In similar settings, time-based FRET acquisition of LUVs incubated with each individual polymer (i.e. donor and acceptor separately) and of individual and mixed polymers without LUVs were also recorded as controls.

The normalized FRET intensity (*y*) was obtained by using the following equation:

$$y = \frac{(P_{(Cy3\text{-}Cy5)} + LUVs) - P_{(Cy3\text{-}Cy5)}}{P_{(Cy5)}}$$

Where $P_{(Cy3\text{-}Cy5)}$+*LUVs* represents the FRET signal of liposomes incubated with premixed donor (cy3) and acceptor (cy5) labelled polymers , $P_{(Cy3\text{-}Cy5)}$ is the FRET signal of the mixed labelled polymers alone (without LUVs) and $P_{(Cy5)}$ is the FRET emission intensity of the acceptor dye without vesicles.

Quartz Crystal Microbalance with Dissipation monitoring (QCM-D)

QCM-D measurements were performed on a four-chamber Q-Sense E4 system (Biolin Scientific) using silicon oxide sensor crystals (QSX 303) cleaned with acetone methanol, milliQ water, then air-dried, and finally, plasma cleaned. After placing the crystals in the QCM chambers, resonance frequency (Δf) and dissipation (ΔD) shifts were collected from 3rd, 5th, 7th, and 9th overtones throughout all the experiment. Δf and ΔD were initially calibrated with a flow of degassed phosphate buffered saline (PBS 1X) at a constant flow rate of 100 µL min^{-1} using an IPC peristaltic pump. A liposomes suspension (0.5 mg mL^{-1}) composed of DOPC:DOPS (9:1) was then injected to form a typical solid lipid bilayer (SLB) on the surface of SiO_2 crystals. The SLB was rinsed with PBS before injection of polymers solutions (150 µg mL^{-1}). The flow of polymer was maintained until stable signals of Δf and ΔD were obtained. Subsequent rinses, first with PBS, then with MilliQ water and finally with the surfactant sodium dodecyl sulfate (2%) were applied to clean the surface. To prevent the formation of air bubbles in the tubing system, the flow rate was interrupted shortly each time the solutions were changed. Recorded values from the 5th overtone were used to determine the mass of polymers adsorbed on the supported lipid layers using the modified Sauerbrey equation:

$$\frac{m}{\mathrm{A}} = -\frac{C}{n}\left(\Delta f + \frac{f_c.\Delta D}{2}\right)$$

where *m/A* is the adsorbed mass normalized against the apparent area, f_c represents the resonance frequency of the crystal (f_c = 4.95 MHz), *C* is the crystal constant (*C*= 17.7 ng cm^{-2}), and *n* is the overtone number (*n* = 5).

Polymer binding to Giant Unilamellar Vesicles (GUVs)

GUVs were produced using the electroformation method on indium tin oxide coated (ITO) glass surfaces.[4] The used Teflon spacer created a volume of approximately 200 µl inside the chamber. The following three lipid mixtures have been used: 1) RBC mimic with 90.3 mol% 1,2-dioleoyl-sn-glycero-3-phosphocholine (DOPC) : 9.7 mol% 1,2-dioleoyl-sn-glycero-3-phospho-L-serine (DOPS) : 0.001 mol% 1-palmitoyl-2-(dipyrrometheneboron difluoride)undecanoyl-sn-glycero-3-phosphocholine (TopFluor-PC), 2) *E. coli* mimic with 71.1 mol% 1-palmitoyl-2-oleoyl-sn-glycero-3-phosphoethanolamine (POPE) : 10 mol% 1-palmitoyl-2-oleoyl-glycero-3-phosphocholine (POPC) : 18.9 mol% 1-palmitoyl-2-oleoyl-sn-

glycero-3-phospho-(1'-rac-glycerol) (POPG) : 0.002 mol% TopFluor-PC and 3) *S. aureus* mimic with 74.5 mol% POPG : 25.5 mol% 1',3'-bis[1,2-dioleoyl-sn-glycero-3-phospho]-glycerol (18:1 Cardiolipin) : 0.002 mol% TopFluor-PC. All lipids were purchased from Avanti Polar Lipids, Inc., Alabaster, AL, USA.

Briefly, 1 µl of a 2.5 mg mL^{-1} lipid mixture in chloroform was homogeneously spread and dried on the ITO glass slides. Then, the chamber was assembled and filled with a 14 mg mL^{-1} sucrose solution in deionized water. The GUVs were formed by applying a sinusoidal electric field with an amplitude (peak-to-peak) of 1.2 V and a frequency of 10 Hz for 1 to 1.5 hours using a voltage generator (Voltcraft FG-2502). Afterwards the chambers were manually slowly flushed with solutions in the following order: 800 µl of 10 mM phosphate buffer with 10 mM sodium chloride, 200 µl of approximately 20 nM Alexa405-labelled polymer, incubated for 10 minutes at room temperature and again flushed with 800 µl 10 mM phosphate buffer with 10 mM sodium chloride.

To follow the solution exchange from buffer to polymer solution via fluorescence microscopy time series measurements, a syringe pump (WPI AL-1010) with a speed set to 150 µl min^{-1} was used for this step.

Fluorescence images were acquired on a Zeiss LSM780 system (Carl Zeiss Microscopy GmbH, Oberkochen, Germany). Briefly, the samples were excited with a 488 nm Argon or a 405 nm diode laser through a Plan-Apochromat 40 ×/1.2 Korr DIC M27 water immersion objective. The fluorescence signal was collected between 499 nm and 552 nm or between 410 nm and 480 nm, respectively. Fluorescence light was detected after passing through a 488/561 nm dichroic mirror using a 32-channel GaAsP detector array. The pinhole size was restricted to one airy unit relative to the 488 nm wavelength to minimize out-of-focus signal. All images were acquired in integration mode with a dimension of 512 × 512 pixel and a pixel dwell time of 25.2 µs. All measurements were performed at 23 ± 1 °C.

Images were analyzed using code custom-written in Matlab (The Mathworks, Inc., USA). Briefly, the images were imported using the Bio-Formats toolbox (docs.openmicroscopy.org) and split to display lipid (488 nm excitation) and polymer (405 nm excitation) channels individually. Next, image regions containing fluorescent debris and non-unilamellar vesicles objects were manually excluded. Background intensity was subtracted from each channel individually by calculating the mean intensity of a manually selected image region without any lipid or polymer fluorescence. The lipid channel intensity (I_{lip}) and pixel number (N_{lip}) were calculated by identifying the pixels in the lipid channel with a value above zero (i.e., above the background values) and calculating their average value and their amount, respectively. The polymer channel intensity (I_{poly}) and pixel number (N_{poly}) were calculated by identifying the pixels in the polymer channel with a value above zero (i.e., above the background values) in both polymer and lipid channels and calculating their average value and their amount,

respectively. In other words, N_{lip} indicates the number of pixels occupied by lipid membranes and N_{poly} indicates the number of pixels occupied by lipid-bound polymer. Finally, the polymer binding to the GUVs was calculated as $I_{poly} \cdot N_{poly} / N_{lip}$, i.e. the total signal originating from lipid-bound polymer, normalized to the amount of lipid membranes in each image.

Polymer binding on bacteria

E. coli DH5alpha bacteria were grown in Luria/Bertani medium (Carl Roth) at 37 °C up to an optical density of 0.4 measured in cuvettes of 1 cm path length. The bacteria suspension was mixed 1:1 with either PBS (Thermo Scientific) (to prepare live and dead controls) or solutions of ca. 40 nM Alexa405-labelled polymer. After incubation for 10 minutes in the presence of ethanol or 1 hour in the presence of polymer at room temperature, bacterial suspensions were centrifuged at 6200 g for 6 minutes. Pellets were resuspended in Luria/Bertani medium and used for fluorescence microscopy imaging. For imaging of polymer distribution over single bacteria, the samples were mixed 3 to 1 with 2% agarose solution to suppress bacterial movement. Fluorescence images were taken as described in previous section.

Investigations of antibacterial activity on biofilms

To investigate the antimicrobial efficacy of polymer MAM70 on mature biofilms, short-term batch experiments were conducted. A dilution streak of *Staphylococcus aureus* (ATCC 6538/ DSM 799) was performed on Müller-Hinton agar plates (Carl Roth), followed by incubation for 24 hours at 80 rpm shaking speed (37 °C, >98% r.H.). A single colony was then picked, resuspended in tryptic soy broth (TSB, Carl Roth) and incubated under the same conditions for 5 hours. The optical density (OD_{600}) was adjusted to 0.15 using a NanoDrop One Microvolume UV-Vis spectrophotometer (Thermo Scientific), corresponding to a viable cell count of 6.7 × 10^7 CFU/mL, as determined by plate counting.

For biofilm formation, the cell suspension was added to 12-well polystyrene plates (2 mL per well) and incubated for 12 hours at 80 rpm shaking speed (37 °C, >98% r.H.). After removing the supernatant, fresh TSB was added to each well, and the plates were incubated for an additional 24 hours under the same conditions.

Following incubation, the supernatant was removed, and 1.7 mL of fresh TSB along with 0.3 mL of test solution were added to each well. The test solutions included:

- Positive control: 70% (v/v) 2-propanol (Carl Roth) in water.

- Negative control: Double-distilled water (ddH_2O).
- Test sample: Aqueous solution of polymer MAM70 at a final concentration of 1024 µg/mL

The plates were then incubated for another 24 hours at 80 rpm shaking speed (37°C, >98% r.H.). Post incubation, the supernatant was removed, and the wells were rinsed three times with ddH_2O, with each rinsing step lasting 3 minutes.

The biofilms were sequentially stained using Syto-9 and propidium iodide. Each sample was incubated for 5 minutes with 50 µL of Syto-9 (12.5 µM in physiological saline) followed by 50 µL of propidium iodide (0.07 mg mL^{-1} in PBS). Images were captured using a fluorescence phase-contrast microscope (BZ-X800E, Keyence) in Z-stack mode.

Supplementary Figures

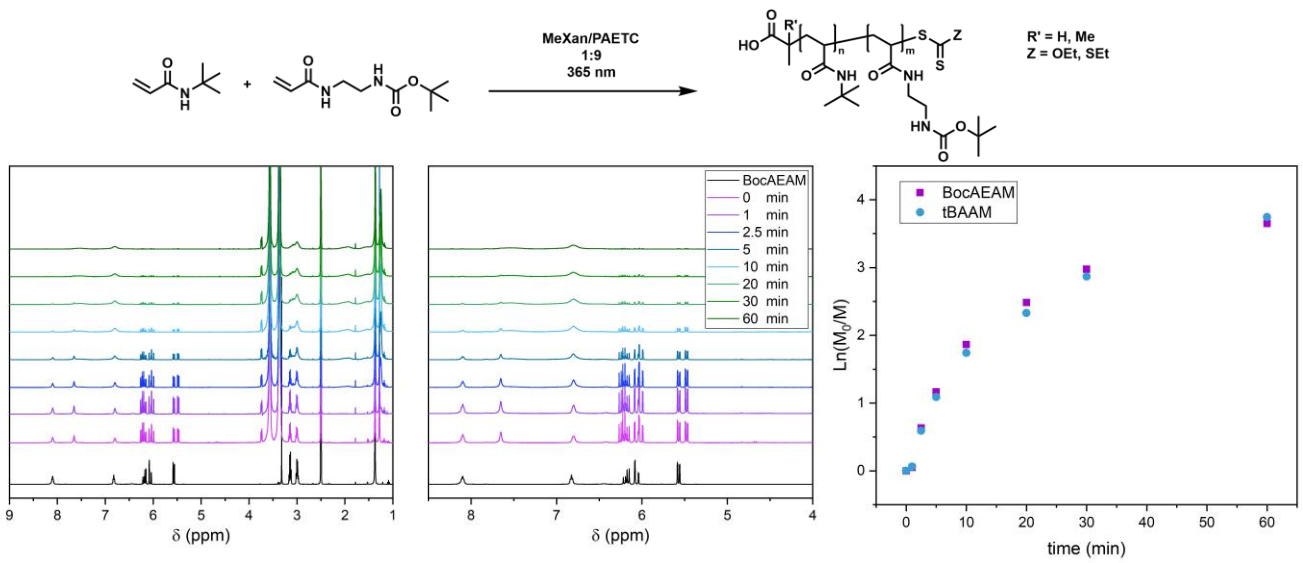

Figure S1: Kinetic investigation of the copolymerization of BocAEAM with TBAM (1:1) using XPI-RAFT polymerization. ^{1}H-NMR spectra (400 MHz) were recorded in DMSO-D_6 and acrylic as well as amide peaks were used to determine the conversion of the respective comonomer.

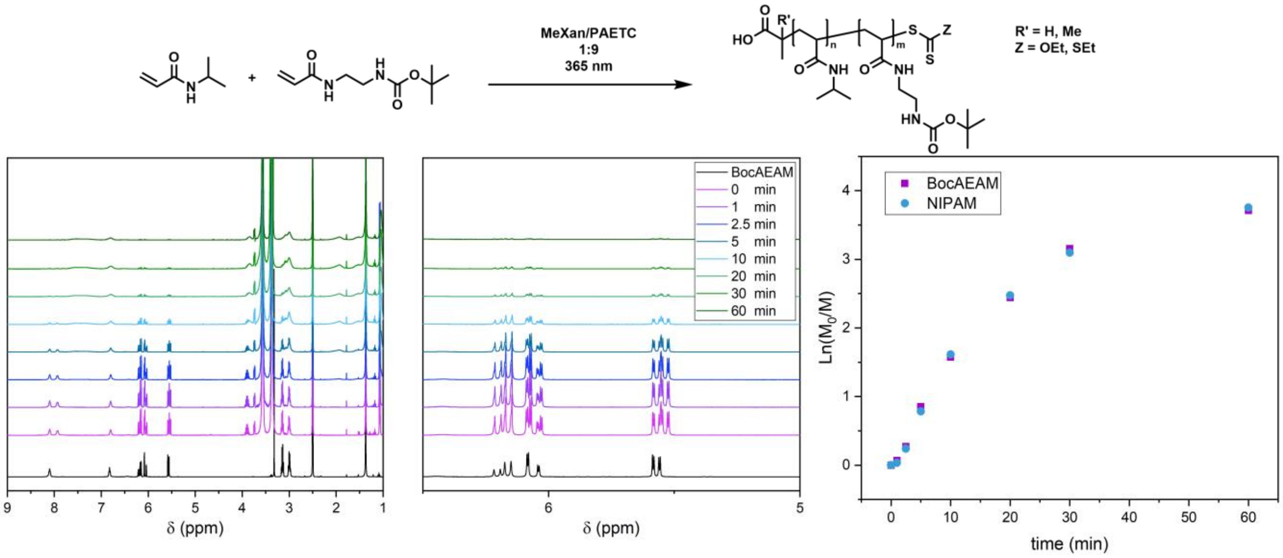

Figure S2: Kinetic investigation of the copolymerization of BocAEAM with NIPAM (1:1) using XPI-RAFT polymerization. ^{1}H-NMR spectra (400 MHz) were recorded in DMSO-D_6 and acrylic as well as amide peaks were used to determine the conversion of the respective comonomer.

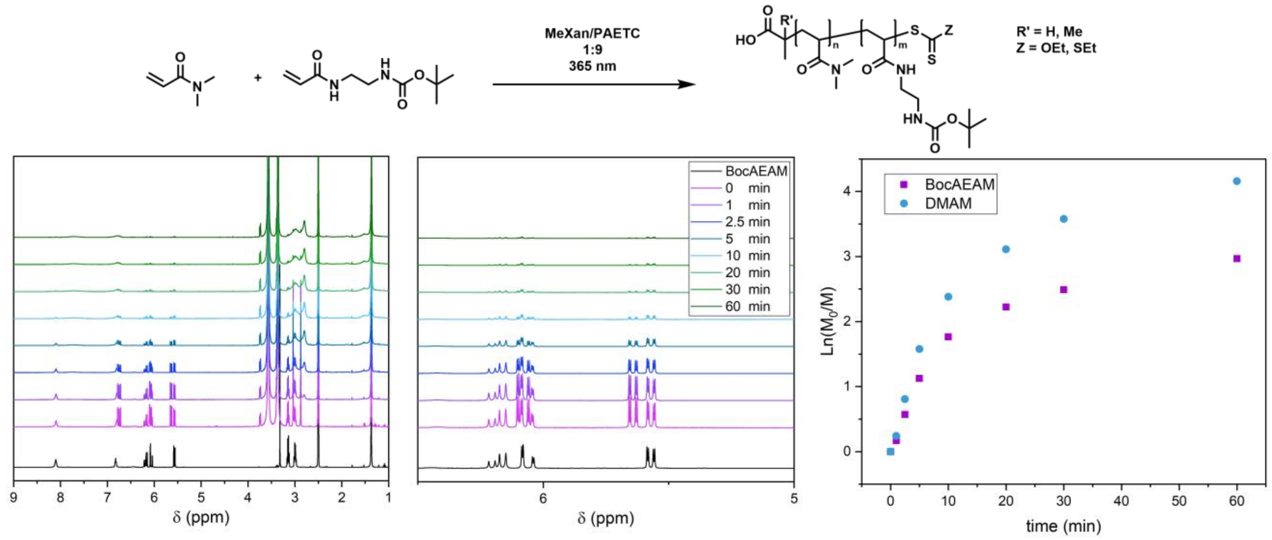

Figure S3: Kinetic investigation of the copolymerization of BocAEAM with DMA (1:1) using XPI-RAFT polymerization. ^{1}H-NMR spectra (400 MHz) were recorded in DMSO-D_6 and acrylic as well as amide peaks were used to determine the conversion of the respective comonomer.

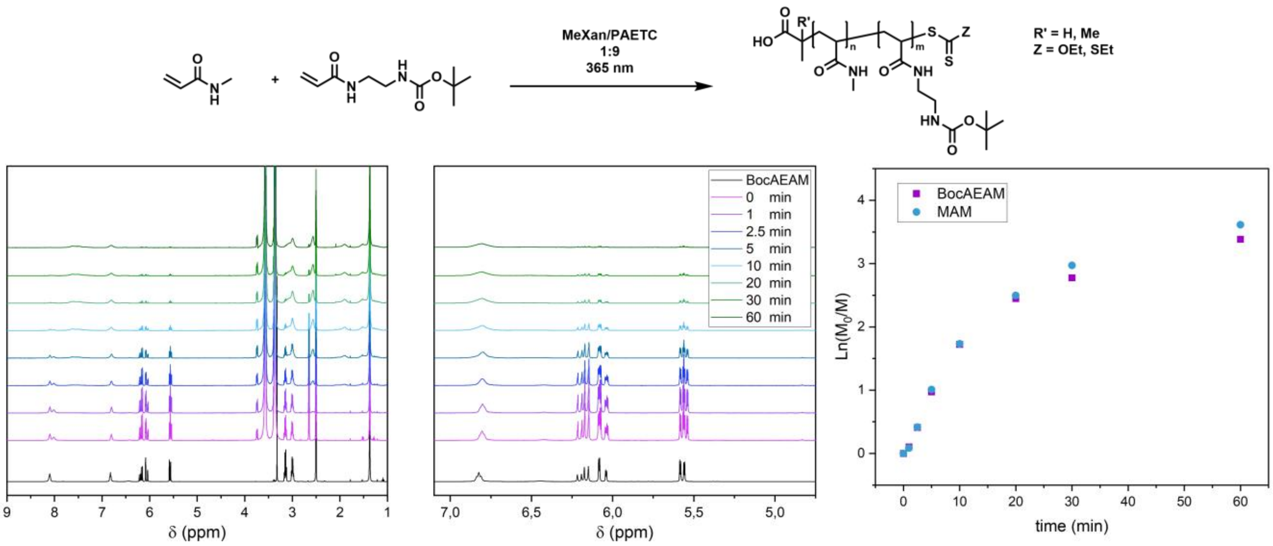

Figure S4: Kinetic investigation of the copolymerization of BocAEAM with MAM (1:1) using XPI-RAFT polymerization. ^{1}H-NMR spectra (400 MHz) were recorded in DMSO-D_6 and acrylic as well as amide peaks were used to determine the conversion of the respective comonomer.

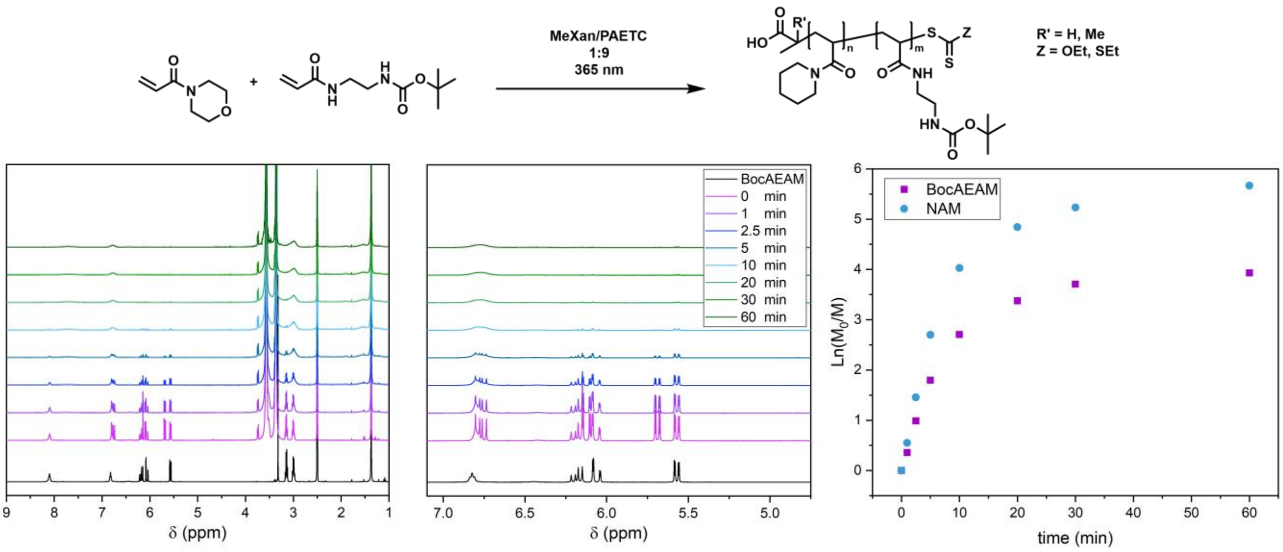

Figure S5: Kinetic investigation of the copolymerization of BocAEAM with NAM (1:1) using XPI-RAFT polymerization. ^{1}H-NMR spectra (400 MHz) were recorded in DMSO-D_6 and acrylic as well as amide peaks were used to determine the conversion of the respective comonomer.

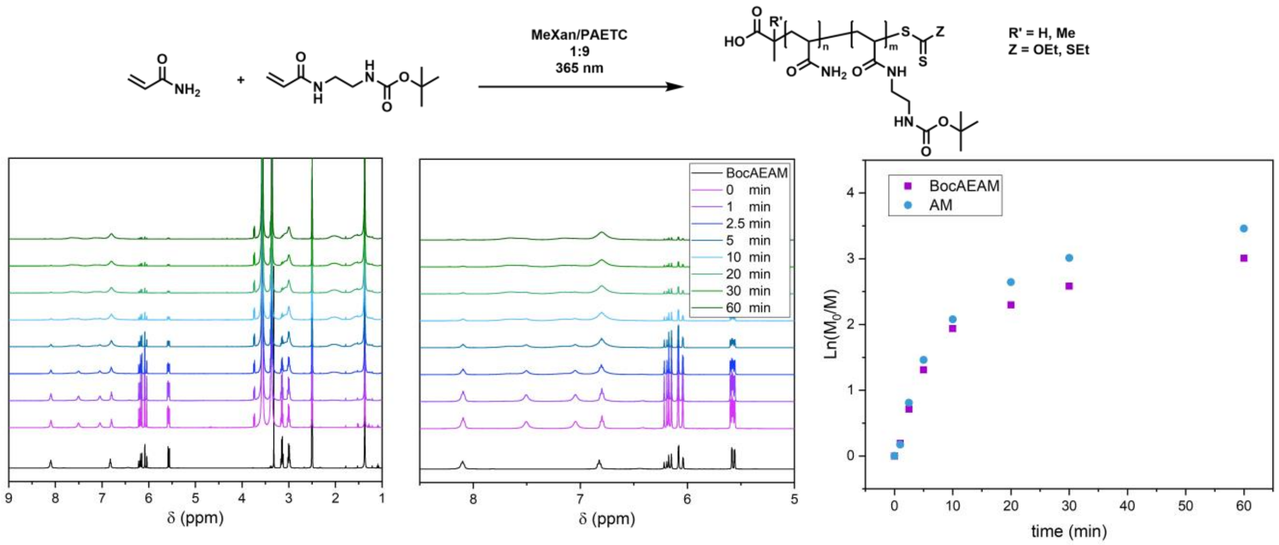

Figure S6: Kinetic investigation of the copolymerization of BocAEAM with AM (1:1) using XPI-RAFT polymerization. ^{1}H-NMR spectra (400 MHz) were recorded in DMSO-D_6 and acrylic as well as amide peaks were used to determine the conversion of the respective comonomer.

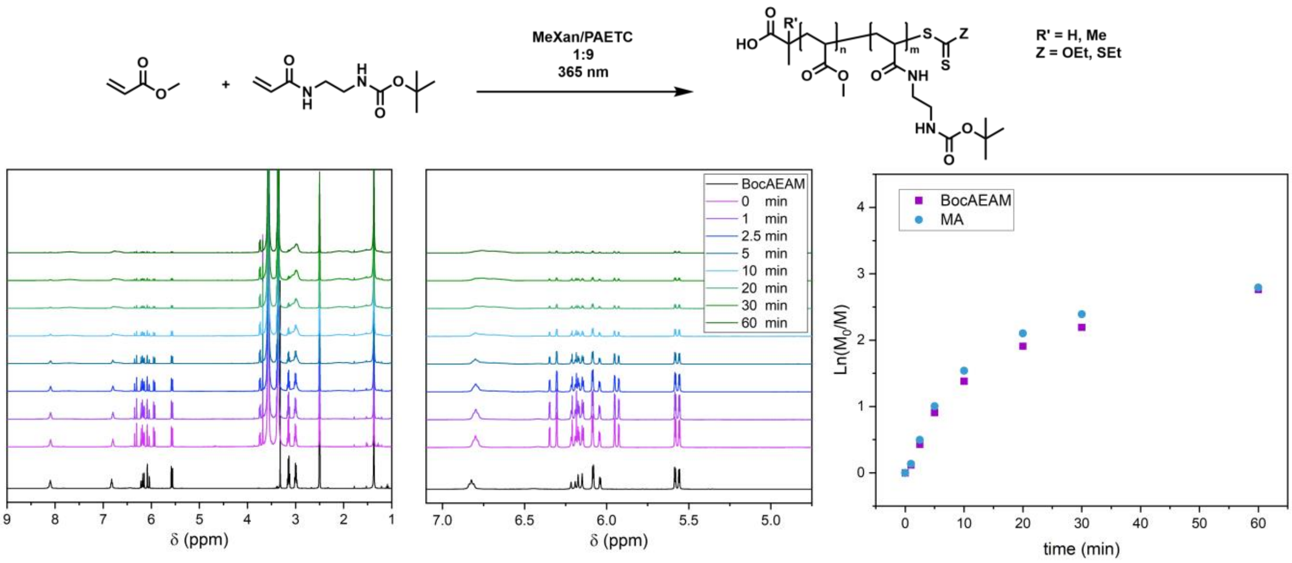

Figure S7: Kinetic investigation of the copolymerization of BocAEAM with MA (1:1) using XPI-RAFT polymerization. ^{1}H-NMR spectra (400 MHz) were recorded in DMSO-D_6 and acrylic as well as amide peaks were used to determine the conversion of the respective comonomer.

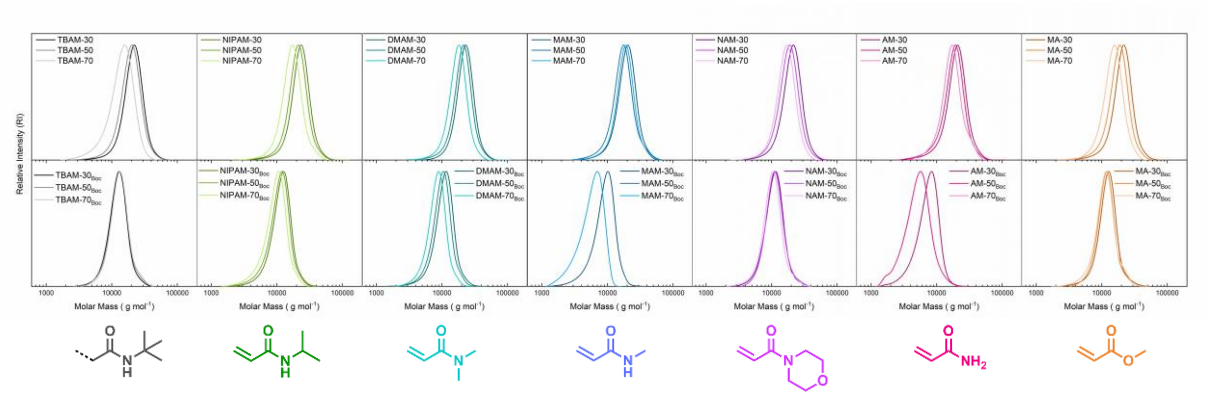

Figure S8: SEC traces of all copolymers before (bottom row, THF, PS calibration) and after (top row, Water (0.3% formic acid, 0.1 M NaCl), PEG calibration) deprotection. 2 Samples (MAM50 and AM70) were not measured before deprotection as they were insoluble in the eluent.

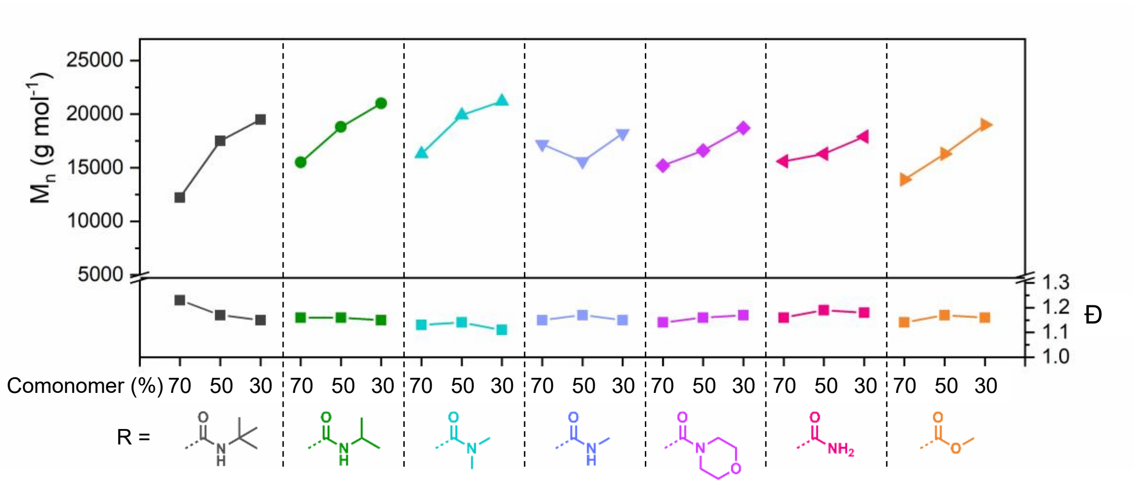

Figure S9: Compiled SEC data for deprotected polymers as a function of type of comonomer and comonomer ratio.

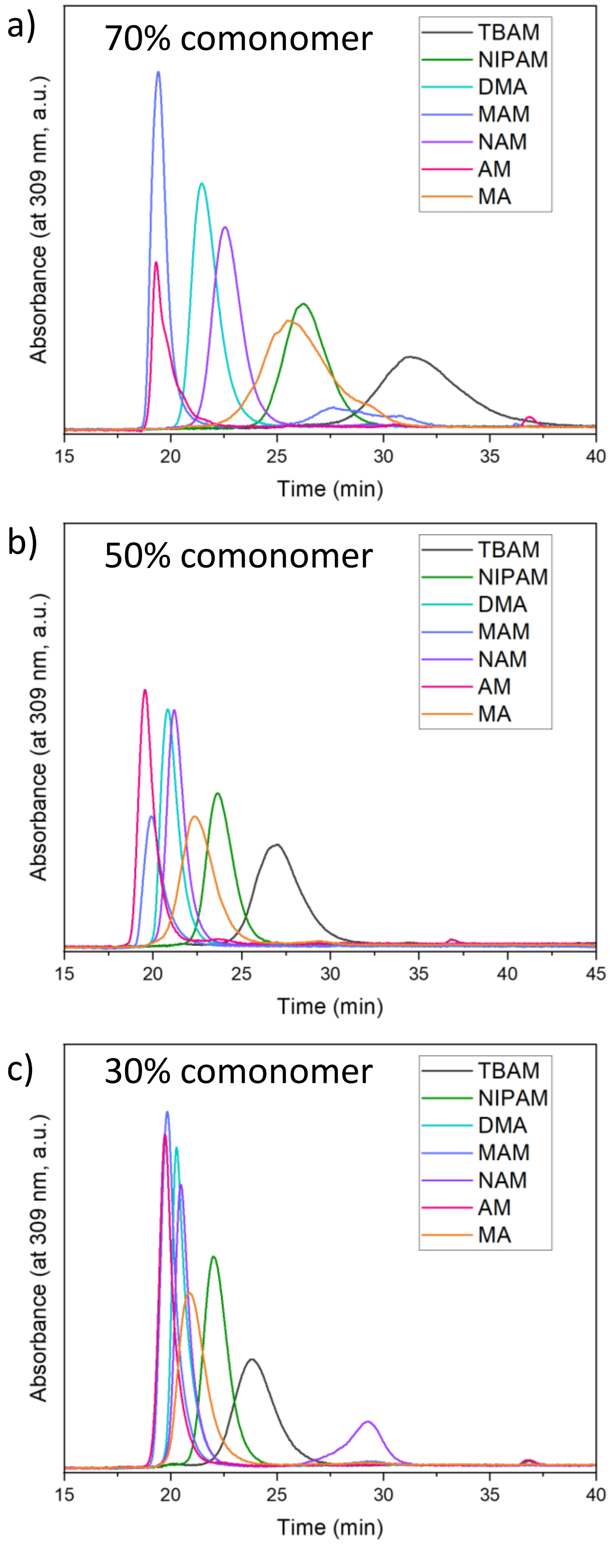

Figure S10: HPLC chromatograms of copolymers with ratio 70%, 50%, and 30% of non-charged comonomer respectively. Polymer samples (5 mg mL^{-1}) were injected in a Pursuit XRs 5 C18 250x4.6 mm column (Agilent) and elution performed at room temperature starting with 1% acetonitrile (99% water) for 10 minutes followed by a gradient up to 95% acetonitrile over 35 minutes.

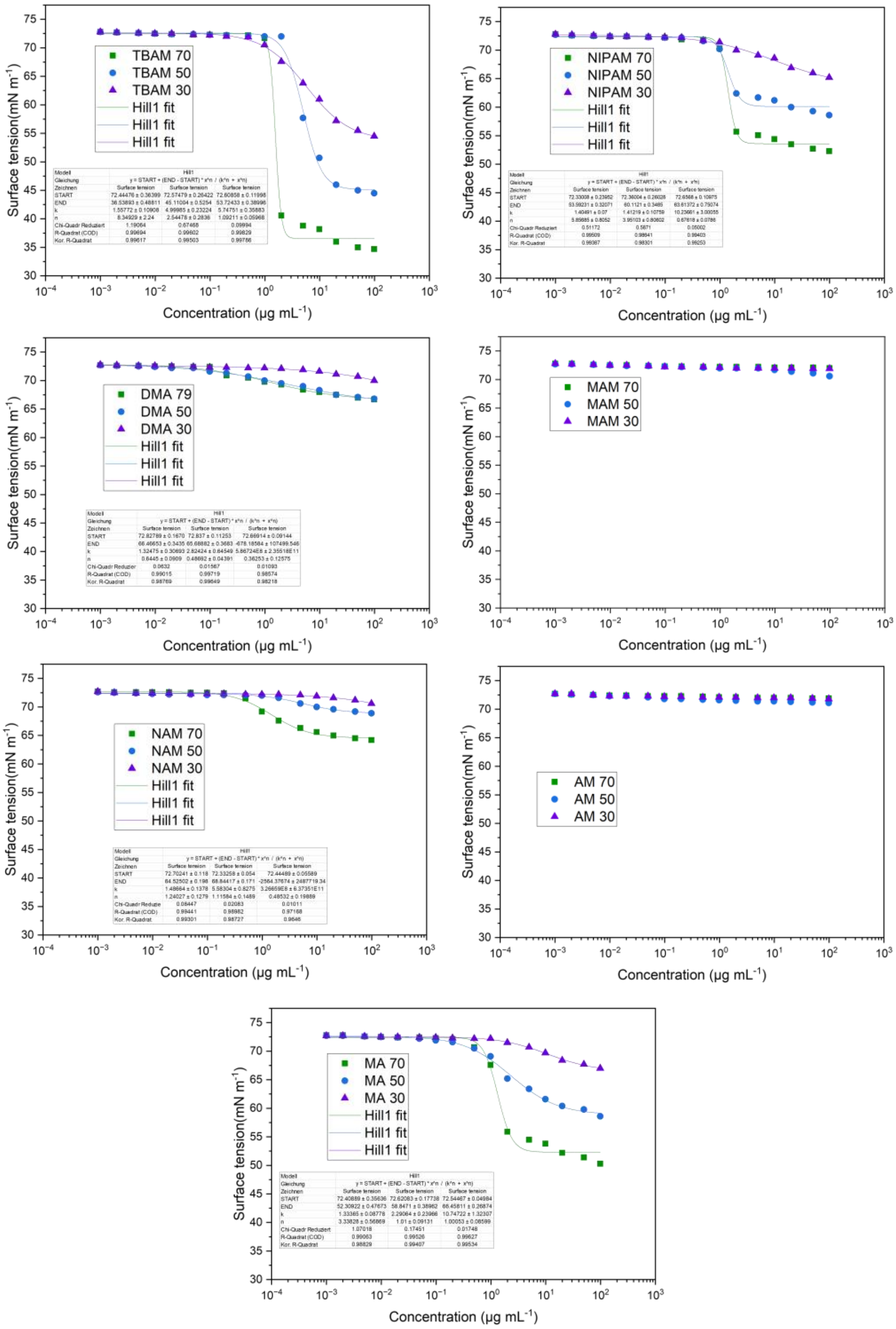

Figure S11: Measurement of the surface activity of copolymers by change of surface tension as a function of polymer concentration determined using a Kibron Wilhelmy plate. Polymer stock solutions were gradually added to the PBS subphase (from 0.001 µg mL^{-1} to 100 µg mL^{-1}) and left to equilibrate for about 100 seconds before the next polymer injection and the decrease in surface tension was plotted against the respective final polymer concentrations using Origin Hill1 fit.

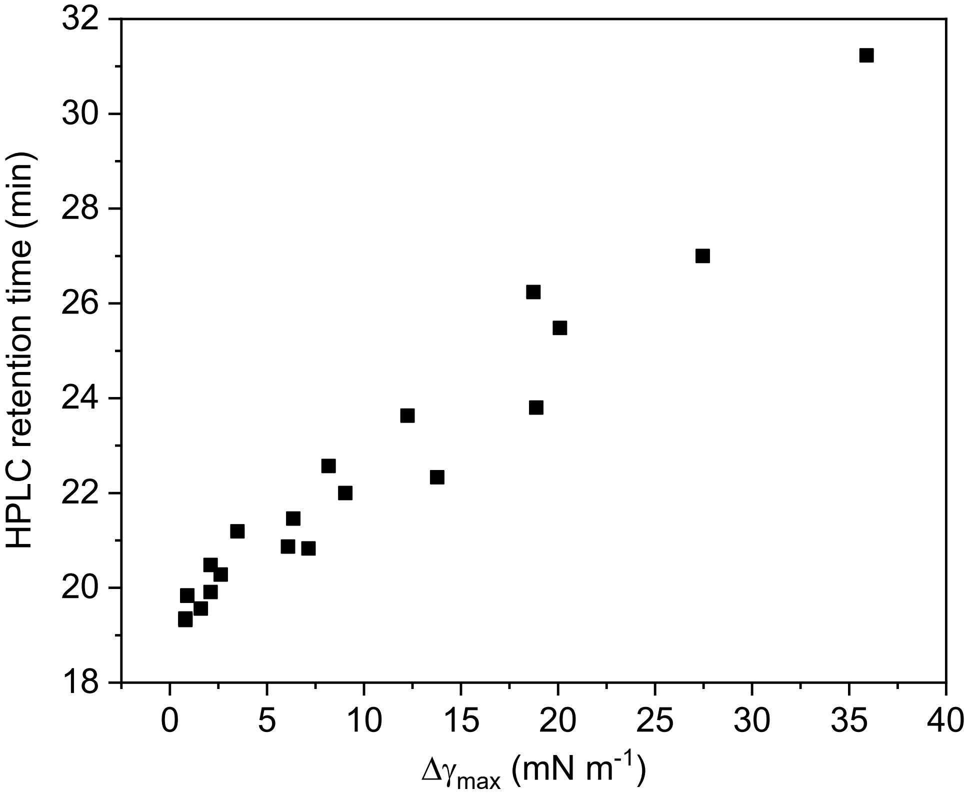

Figure S12: Correlation between HPLC retention time and change in surface tension of copolymers.

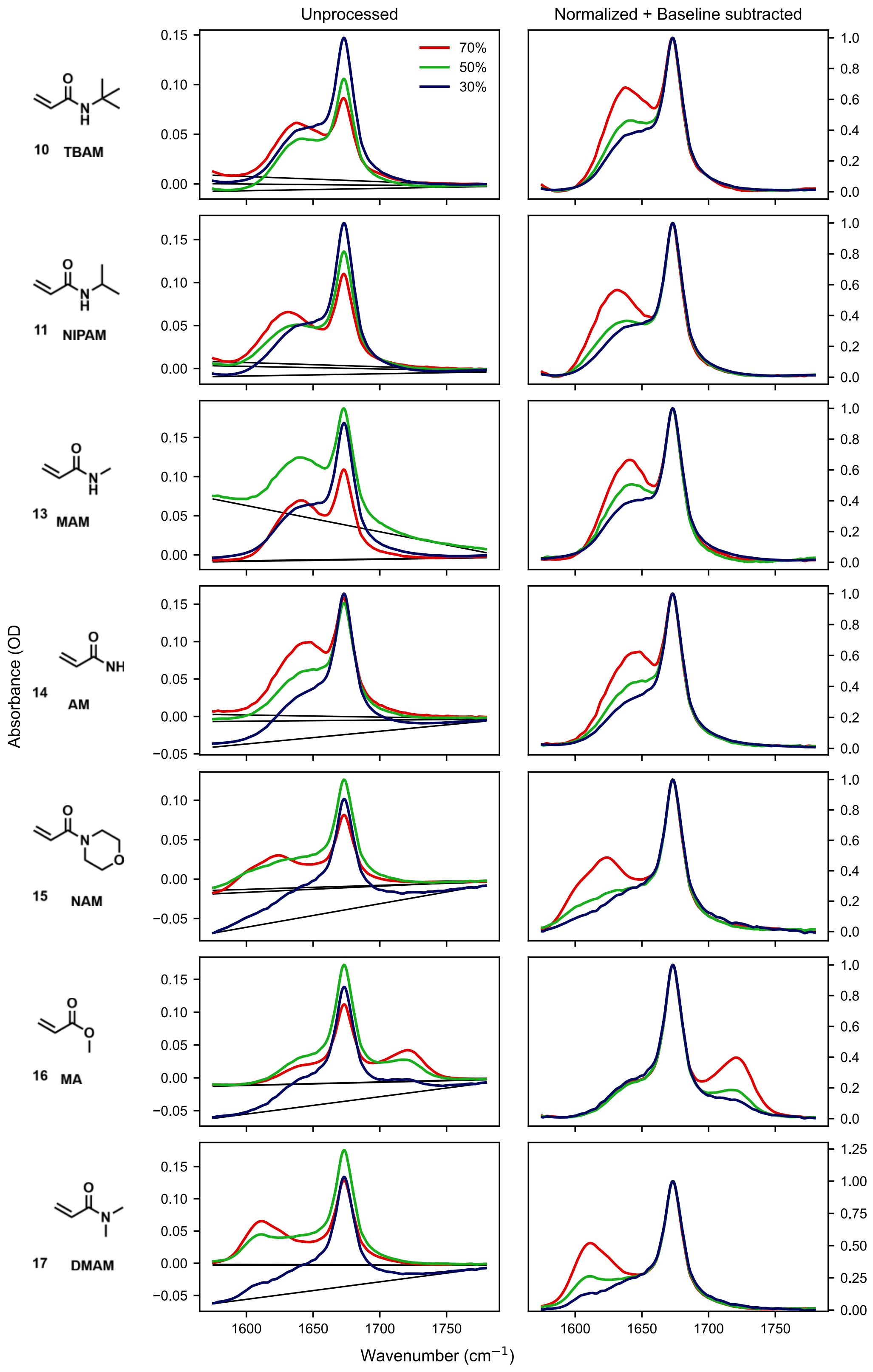

Figure S13: FTIR spectra of different polymers measured at a concentration of 10 mg ml^{-1} in D_2O. The left column shows the raw spectra of the three polymer ratios and the estimated baseline (black). The right column shows the spectra after subtraction of the baseline and normalization to the 1674 cm^{-1}

peak. The spectra in the main text are calculated by subtraction of the normalized 30% spectra from the normalized 70% spectra.

Model	DoseResp		
Equation	y = A1 + (A2-A1)/(1 + 10^((LOGx0-x)*p))		
Plot	tBAM (70%)	tBAM (50%)	tBAM (30%)
A1	3.47542 ± 0	2.05453 ± 0	20.13862 ± 2.70489
A2	106.91244 ± 0	95.94854 ± 0	79.03494 ± 0
LOGx0	13.82841 ± 4.97492	9.58783 ± 7.52158	40.82661 ± 4.28926
p	-0.07197 ± 0.02941	-0.4391 ± 2.02553	-0.02482 ± 0.00335
Reduced Chi-Sqr	28.26605	45.50861	5.74096
R-Square (COD)	0.87634	0.86252	0.97944
Adj. R-Square	0.86397	0.84877	0.97357

Model	DoseResp		
Equation	y = A1 + (A2-A1)/(1 + 10^((LOGx0-x)*p))		
Plot	tBAM (70%)	tBAM (50%)	tBAM (30%)
A1	-4.72434 ± 0.37705	0.80855 ± 0.81282	8.9835 ± 3.3312
A2	116.33361 ± 15.9624	96.01457 ± 0.99319	95.10627 ± 39.80965
LOGx0	33.13025 ± 11.93129	21.18549 ± 0.90683	63.66261 ± 34.8818
p	-0.01491 ± 0.00149	-0.15219 ± 0.02599	-0.0425 ± 1.06966
Reduced Chi-Sqr	0.61105	3.16449	1207.76035
R-Square (COD)	0.99903	0.99887	0.8343
Adj. R-Square	0.99866	0.99844	0.77217

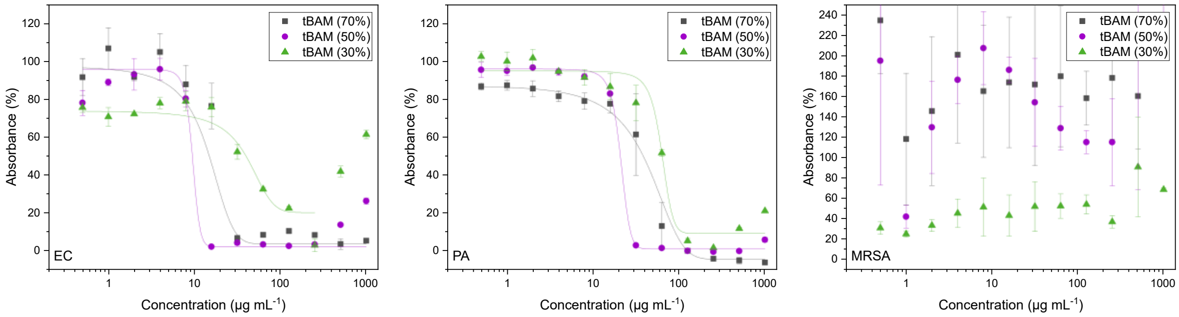

Figure S14: Determination of MIC values of TBAM containing copolymers via growth inhibition assay using either EC , PA, or MRSA. Data were fitted using a dose response function in Origin software. Increased OD at higher polymer concentration are a result aggrgation of buffer components by the polymers.

Model	DoseResp	
Equation	y = A1 + (A2-A1)/(1 + 10^((LOGx0-x)*p))	
Plot	NIPAM (70%)	NIPAM (50%)
A1	-0.24034 ± 0	1.18405 ± 0
A2	85.81993 ± 0	102.03573 ± 0
LOGx0	74.17835 ± 5.96833	23.23075 ± 4.02181
p	-0.01187 ± 0.00126	-0.0519 ± 0.01979
Reduced Chi-Sqr	16.1792	70.61403
R-Square (COD)	0.99177	0.91246
Adj. R-Square	0.99095	0.90371

Model	DoseResp		
Equation	y = A1 + (A2-A1)/(1 + 10^((LOGx0-x)*p))		
Plot	NIPAM (70%)	NIPAM (50%)	NIPAM(30%)
A1	0 ± 0	5 ± 0	13.90047 ± 3.96271
A2	103.92849 ± 0	110.53885 ± 0	126.20932 ± 38.53169
LOGx0	927.66748 ± 41.88522	113.30379 ± 38.92319	67.73955 ± 36.17548
p	-0.00153 ± 1.02271E-4	-0.01035 ± 0.00422	-0.00997 ± 0.00701
Reduced Chi-Sqr	7.74153	42.56948	46.11689
R-Square (COD)	0.94778	0.98695	0.97803
Adj. R-Square	0.94256	0.98565	0.96879

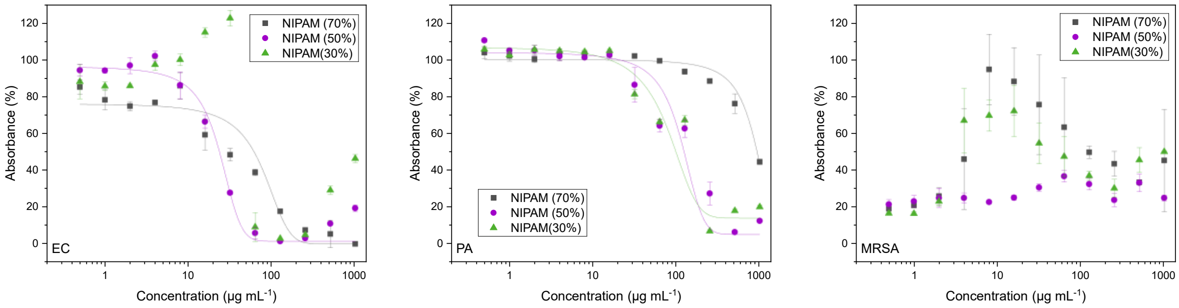

Figure S15: Determination of MIC values of NIPAM containing copolymers via growth inhibition assay using either EC , PA, or MRSA. Data were fitted using a dose response function in Origin software. Increased OD at higher polymer concentration are a result aggrgation of buffer components by the

polymers.

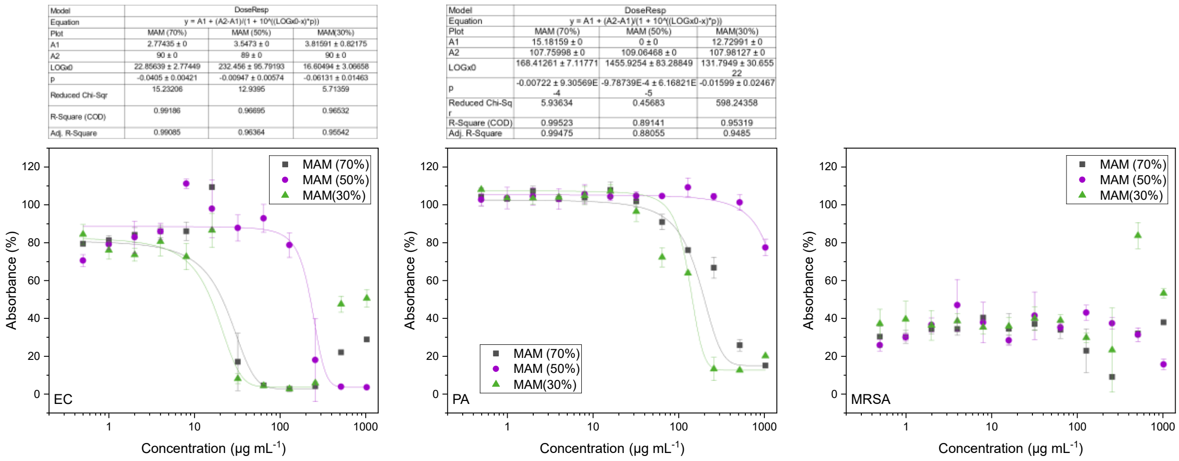

Model	DoseResp		
Equation	y = A1 + (A2-A1)/(1 + 10^((LOGx0-x)*p))		
Plot	MAM (70%)	MAM (50%)	MAM(30%)
A1	2.77435 ± 0	3.5473 ± 0	3.81591 ± 0.82175
A2	90 ± 0	89 ± 0	90 ± 0
LOGx0	22.85639 ± 2.77449	232.456 ± 95.79193	16.60494 ± 3.06658
p	-0.0405 ± 0.00421	-0.00947 ± 0.00574	-0.06131 ± 0.01463
Reduced Chi-Sqr	15.23206	12.9395	5.71359
R-Square (COD)	0.99186	0.96695	0.96532
Adj. R-Square	0.99085	0.96364	0.95542

Model	DoseResp		
Equation	y = A1 + (A2-A1)/(1 + 10^((LOGx0-x)*p))		
Plot	MAM (70%)	MAM (50%)	MAM(30%)
A1	15.18159 ± 0	0 ± 0	12.72991 ± 0
A2	107.75998 ± 0	109.06468 ± 0	107.98127 ± 0
LOGx0	168.41261 ± 7.11771	1455.9254 ± 83.28849	131.7949 ± 30.65522
p	-0.00722 ± 9.30569E-4	-9.78739E-4 ± 6.16821E-5	-0.01599 ± 0.02467
Reduced Chi-Sqr	5.93634	0.45683	598.24358
R-Square (COD)	0.99523	0.89141	0.95319
Adj. R-Square	0.99475	0.88055	0.9485

Figure S16: Determination of MIC values of MAM containing copolymers via growth inhibition assay using either EC , PA, or MRSA. Data were fitted using a dose response function in Origin software. Increased OD at higher polymer concentration are a result aggrgation of buffer components by the polymers.

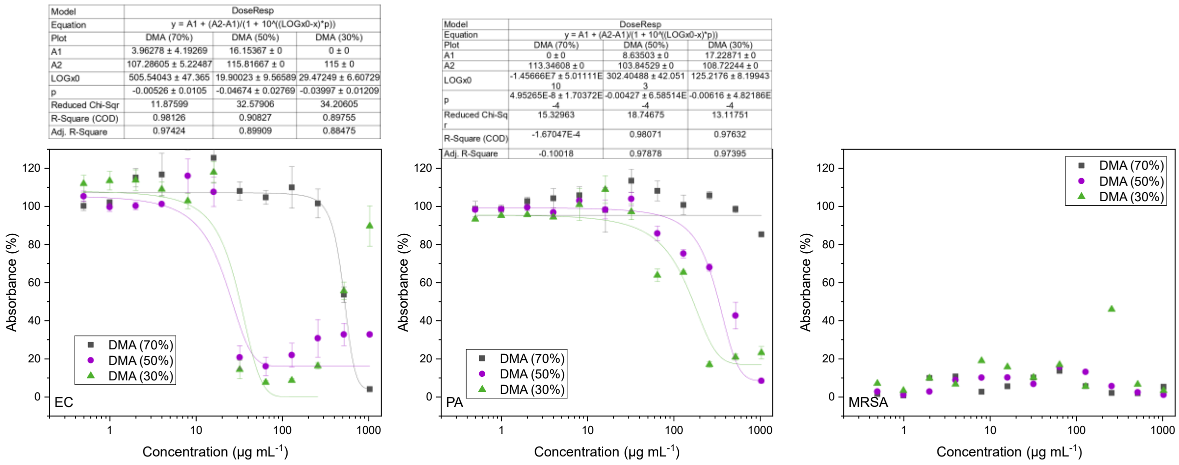

Model	DoseResp		
Equation	y = A1 + (A2-A1)/(1 + 10^((LOGx0-x)*p))		
Plot	DMA (70%)	DMA (50%)	DMA (30%)
A1	3.96278 ± 4.19269	16.15367 ± 0	0 ± 0
A2	107.28605 ± 5.22487	115.81667 ± 0	115 ± 0
LOGx0	505.54043 ± 47.365	19.90023 ± 9.56589	29.47249 ± 6.60729
p	-0.00526 ± 0.0105	-0.04674 ± 0.02769	-0.03997 ± 0.01209
Reduced Chi-Sqr	11.87599	32.57906	34.20605
R-Square (COD)	0.98126	0.90827	0.89755
Adj. R-Square	0.97424	0.89909	0.88475

Model	DoseResp		
Equation	y = A1 + (A2-A1)/(1 + 10^((LOGx0-x)*p))		
Plot	DMA (70%)	DMA (50%)	DMA (30%)
A1	0 ± 0	8.63503 ± 0	17.22871 ± 0
A2	113.34608 ± 0	103.84529 ± 0	108.72244 ± 0
LOGx0	-1.45666E7 ± 5.01111E10	302.40488 ± 42.0513	125.2176 ± 8.19943
p	4.95265E-8 ± 1.70372E-4	-0.00427 ± 6.58514E-4	-0.00616 ± 4.82186E-4
Reduced Chi-Sqr	15.32963	18.74675	13.11751
R-Square (COD)	-1.67047E-4	0.98071	0.97632
Adj. R-Square	-0.10018	0.97878	0.97395

Figure S17: Determination of MIC values of DMA containing copolymers via growth inhibition assay using either EC , PA, or MRSA. Data were fitted using a dose response function in Origin software. Increased OD at higher polymer concentration are a result aggrgation of buffer components by the

polymers.

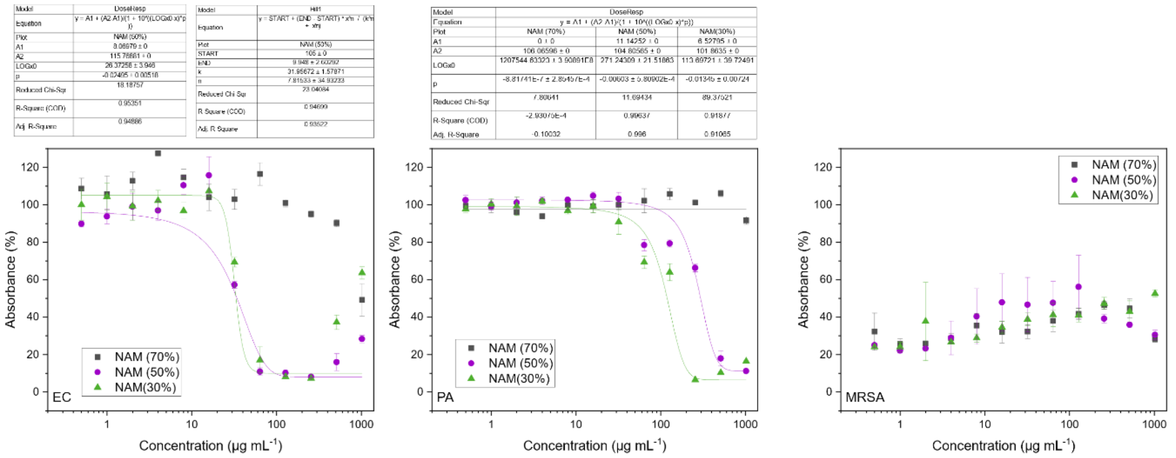

Figure S18: Determination of MIC values of NAM containing copolymers via growth inhibition assay using either EC , PA, or MRSA. Data were fitted using a dose response function in Origin software. Increased OD at higher polymer concentration are a result aggrgation of buffer components by the polymers.

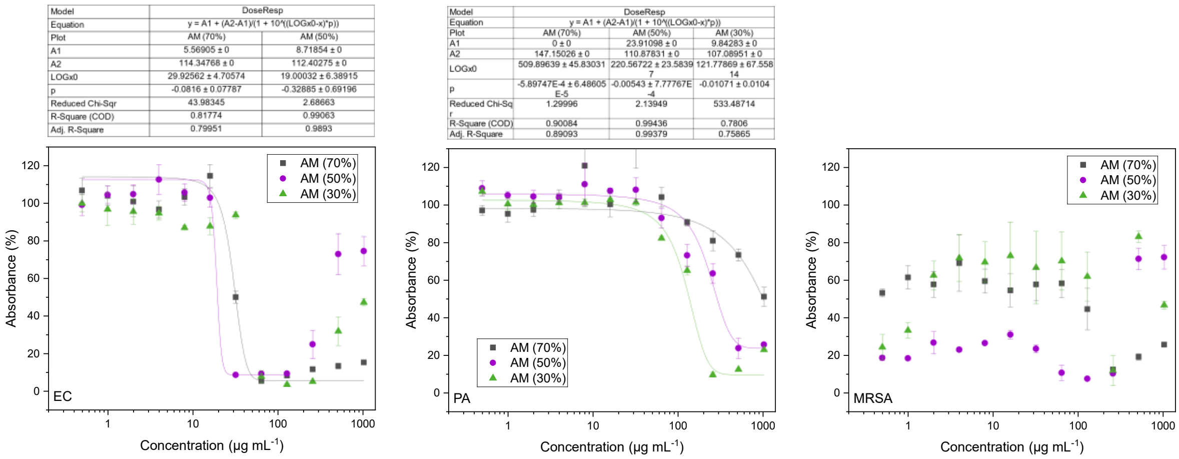

Figure S19: Determination of MIC values of AM containing copolymers via growth inhibition assay using either EC , PA, or MRSA. Data were fitted using a dose response function in Origin software. Increased OD at higher polymer concentration are a result aggrgation of buffer components by the

polymers.

Model	DoseResp	
Equation	y = A1 + (A2-A1)/(1 + 10^((LOGx0-x)*p))	
Plot	MA (70%)	MA (50%)
A1	5.5831 ± 0	3.52564 ± 0
A2	89.93318 ± 0	98.01282 ± 0
LOGx0	10.10591 ± 0.90185	25.65001 ± 5.63045
p	-0.06844 ± 0.01271	-0.05708 ± 0.02758
Reduced Chi-Sqr	1.4104	22.62183
R-Square (COD)	0.98358	0.96092
Adj. R-Square	0.98194	0.95604

Model	DoseResp		
Equation	y = A1 + (A2-A1)/(1 + 10^((LOGx0-x)*p))		
Plot	MA (70%)	MA (50%)	MA (30%)
A1	0.41157 ± 0	1.13191 ± 0	1.78191 ± 0
A2	102.30936 ± 0	102.11812 ± 0	104.57245 ± 0
LOGx0	53.19698 ± 2.13722	66.89809 ± 12.30797	67.65348 ± 7.31506
p	-0.0451 ± 0.00414	-0.02163 ± 0.00903	-0.02371 ± 0.0151
Reduced Chi-Sqr	7.80548	50.67961	205.03866
R-Square (COD)	0.99966	0.97172	0.96027
Adj. R-Square	0.99963	0.96889	0.95629

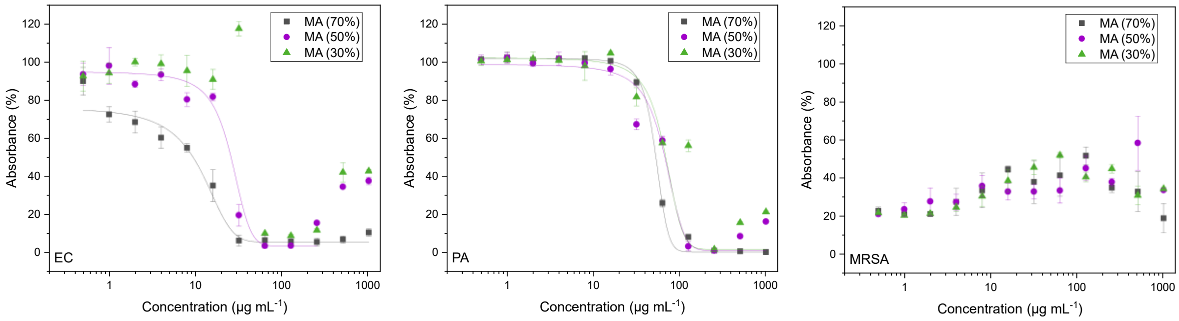

Figure S20: Determination of MIC values of MA containing copolymers via growth inhibition assay using either EC , PA, or MRSA. Data were fitted using a dose response function in Origin software. Increased OD at higher polymer concentration are a result aggrgation of buffer components by the polymers.

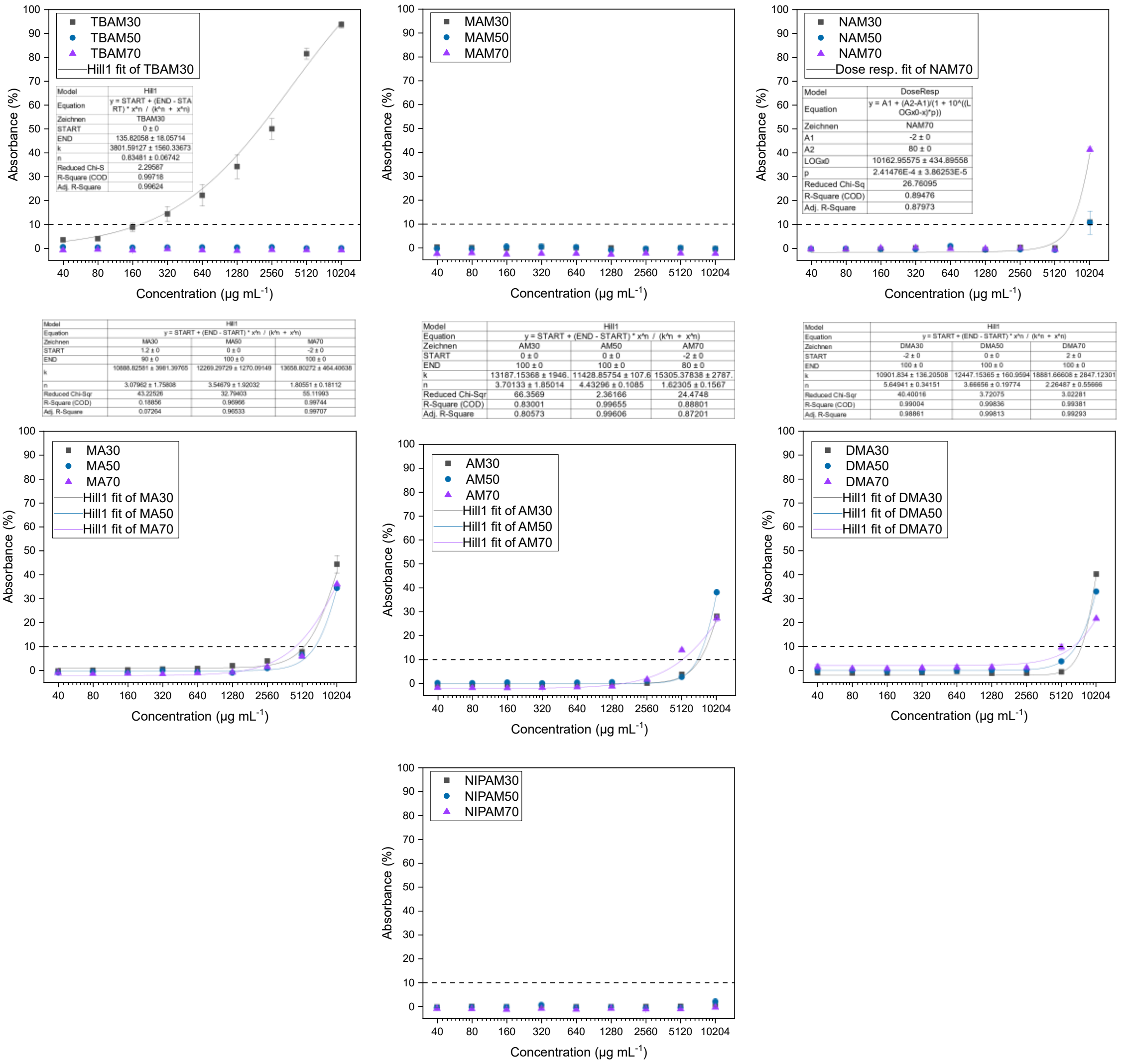

Model	Hill1
Equation	y = START + (END - START) * x^n / (k^n + x^n)
Zeichnen	TBAM30
START	0 ± 0
END	135.82058 ± 18.05714
k	3801.59127 ± 1560.33673
n	0.83481 ± 0.06742
Reduced Chi-S	2.29587
R-Square (COD	0.99718
Adj. R-Square	0.99624

Model	DoseResp
Equation	y = A1 + (A2-A1)/(1 + 10^((LOGx0-x)*p))
Zeichnen	NAM70
A1	-2 ± 0
A2	80 ± 0
LOGx0	10162.95575 ± 434.89558
p	2.41476E-4 ± 3.86253E-5
Reduced Chi-Sq	26.76095
R-Square (COD)	0.89476
Adj. R-Square	0.87973

Model	Hill1		
Equation	y = START + (END - START) * x^n / (k^n + x^n)		
Zeichnen	MA30	MA50	MA70
START	1.2 ± 0	0 ± 0	-2 ± 0
END	90 ± 0	100 ± 0	100 ± 0
k	10888.82581 ± 3981.39765	12269.29729 ± 1270.09149	13658.80272 ± 464.40638
n	3.07962 ± 1.75808	3.54679 ± 1.92032	1.80551 ± 0.18112
Reduced Chi-Sqr	43.22526	32.79403	55.11983
R-Square (COD)	0.18856	0.96966	0.99744
Adj. R-Square	0.07264	0.96533	0.99707

Model	Hill1		
Equation	y = START + (END - START) * x^n / (k^n + x^n)		
Zeichnen	AM30	AM50	AM70
START	0 ± 0	0 ± 0	-2 ± 0
END	100 ± 0	100 ± 0	80 ± 0
k	13187.15368 ± 1946.	11428.85754 ± 107.6	15305.37838 ± 2787.
n	3.70133 ± 1.85014	4.43296 ± 0.1085	1.62305 ± 0.1567
Reduced Chi-Sqr	66.3569	2.36166	24.4748
R-Square (COD)	0.83001	0.99655	0.88801
Adj. R-Square	0.80573	0.99606	0.87201

Model	Hill1		
Equation	y = START + (END - START) * x^n / (k^n + x^n)		
Zeichnen	DMA30	DMA50	DMA70
START	-2 ± 0	0 ± 0	2 ± 0
END	100 ± 0	100 ± 0	100 ± 0
k	10901.834 ± 136.20508	12447.15365 ± 160.9594	18881.66608 ± 2847.12301
n	5.64941 ± 0.34151	3.66656 ± 0.19774	2.26487 ± 0.55666
Reduced Chi-Sqr	40.40016	3.72075	3.02281
R-Square (COD)	0.99004	0.99836	0.99381
Adj. R-Square	0.98861	0.99813	0.99293

Figure S21: Hemolysis assay with different copolymers as determined by absorbance measurements at 544 nm after incubation of erythrocytes with copolymers at 37 °C for 1 h. Data were fitted using a Hill1 fit or a dose response function in Origin software.

Modell	Hill1		
Gleichung	y = START + (END - START) * x^n / (k^n + x^n)		
Zeichnen	NIPAM30	NIPAM50	NIPAM70
START	1.14082 ± 0	1.22651 ± 0	1.20774 ± 0
END	0 ± 0	0.02529 ± 0	0.01646 ± 0
k	537119.22389 ± 1798927.05588	1008.36652 ± 71.50593	106.20905 ± 11.68186
n	0.22903 ± 0.09484	9.28167 ± 9.17729	3.13571 ± 0.3682
Chi-Quadr Reduziert	0.20731	0.67856	0.78976
R-Quadrat (COD)	0.45295	0.9934	0.97422
Kor. R-Quadrat	0.39217	0.99266	0.97136

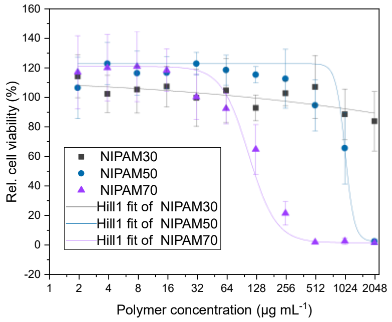

Modell	Hill1		
Gleichung	y = START + (END - START) * x^n / (k^n + x^n)		
Zeichnen	TBAM30	TBAM50	TBAM70
START	1.13904 ± 0	1.09126 ± 0	1.27275 ± 0
END	0.02451 ± 0	0.01484 ± 0	0.01184 ± 0
k	9.81957 ± 2.764	21.33681 ± 2.350	61.32316 ± 7.134
n	3.23644 ± 1.481	3.35278 ± 0.3434	2.88891 ± 0.4872
Chi-Quadr Reduzier	3.03256	0.37835	2.44018
R-Quadrat (COD)	0.70183	0.98775	0.98264
Kor. R-Quadrat	0.6687	0.98639	0.98071

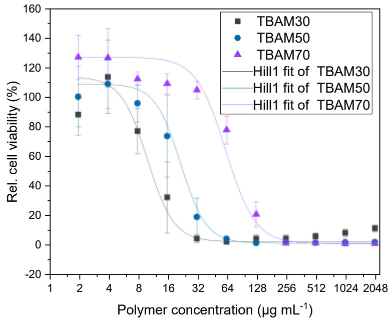

Modell	Hill1		
Gleichung	y = START + (END - START) * x^n / (k^n + x^n)		
Zeichnen	MAM30	MAM50	MAM70
START	1.12795 ± 0	1.14441 ± 0	1.11879 ± 0
END	0.07777 ± 0	0 ± 0	0.02046 ± 0
k	1006.20491 ± 100	79930.16756 ± 128406	92.62372 ± 7.0
n	11.78144 ± 101.1	0.34037 ± 0.09848	2.16828 ± 0.16
Chi-Quadr Redu	4.75856	1.97473	0.35713
R-Quadrat (CO	0.95437	0.61839	0.99569
Kor. R-Quadrat	0.9493	0.57599	0.99521

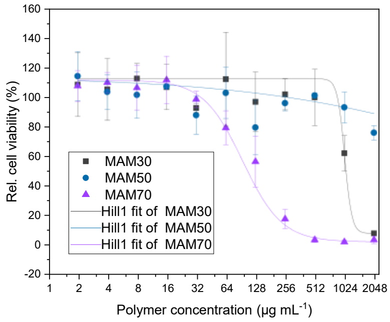

Modell	Hill1		
Gleichung	y = START + (END - START) * x^n / (k^n + x^n)		
Zeichnen	DMA30	DMA50	DMA70
START	0.9 ± 0	1.05667 ± 0	1.14445 ± 0
END	0 ± 0	0.07737 ± 0	0.01285 ± 0
k	2031.98011 ± 119.1	992.35951 ± 67.2	111.46389 ± 12.4
n	1.37547 ± 0.17809	5.99117 ± 4.3397	2.59195 ± 0.2460
Chi-Quadr Redu	0.19152	2.93093	0.81891
R-Quadrat (COD	0.96852	0.90409	0.9741
Kor. R-Quadrat	0.96502	0.89343	0.97122

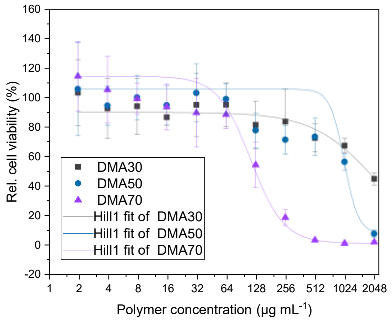

Modell	Hill1		
Gleichung	y = START + (END - START) * x^n / (k^n + x^n)		
Zeichnen	AM30	AM50	AM70
START	1 ± 0	1.19227 ± 0	1.10759 ± 0
END	0.24184 ± 0	0.03494 ± 0	0.02522 ± 0
k	887.05793 ± 185.	134.66521 ± 23.12	38.53388 ± 1.28
n	2.11976 ± 0.7420	2.09866 ± 0.3655	2.14435 ± 0.127
Chi-Quadr Reduzi	1.09522	2.78214	0.55455
R-Quadrat (COD)	0.82619	0.9794	0.99389
Kor. R-Quadrat	0.80688	0.97711	0.99321

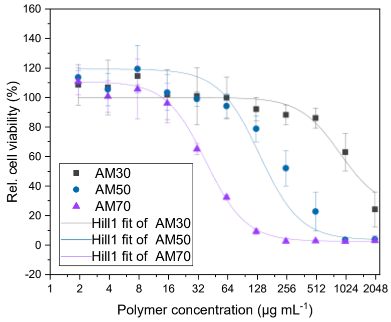

Modell	Hill1		
Gleichung	y = START + (END - START) * x^n / (k^n + x^n)		
Zeichnen	NAM30	NAM50	NAM70
START	1.1007 ± 0	1.15811 ± 0	1.16864 ± 0
END	0 ± 0	0.14423 ± 0	0.00901 ± 0
k	2940.8873 ± 964.0	1020.39072 ± 76.4	248.24468 ± 9.8
n	1.15573 ± 0.28455	5.52475 ± 4.22644	3.61902 ± 0.241
Chi-Quadr Reduz	0.30099	1.67565	0.22206
R-Quadrat (COD	0.69429	0.91287	0.99688
Kor. R-Quadrat	0.66033	0.90319	0.99653

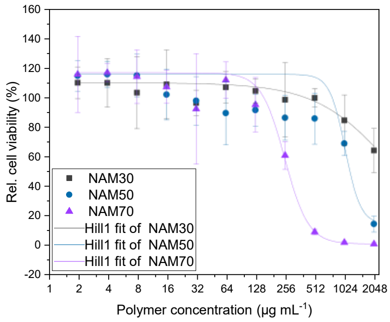

Modell	Hill1		
Gleichung	y = START + (END - START) * x^n / (k^n + x^n)		
Zeichnen	MA30	MA50	MA70
START	1.06095 ± 0	1.05359 ± 0	1.03825 ± 0
END	-0.00237 ± 0	0.00597 ± 0	0.01086 ± 0
k	357.08283 ± 29.64606	160.18536 ± 23.91382	56.66774 ± 6.35128
n	2.30312 ± 0.18243	1.10296 ± 0.14271	2.06071 ± 0.2486
Chi-Quadr Reduziert	0.74266	3.82405	1.43328
R-Quadrat (COD)	0.99736	0.96391	0.94915
Kor. R-Quadrat	0.99706	0.9599	0.9435

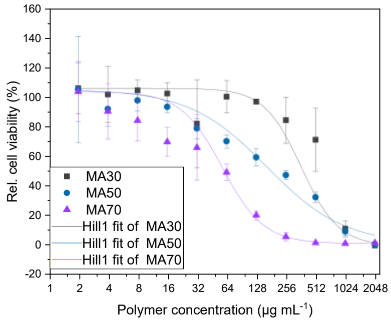

Figure S22: Cytotoxicity of copolymer as determined via an MTT assay using L929 mouse fibroblasts. Cells were incubated with polymers at indicated concentrations at 37 °C for 24 h.

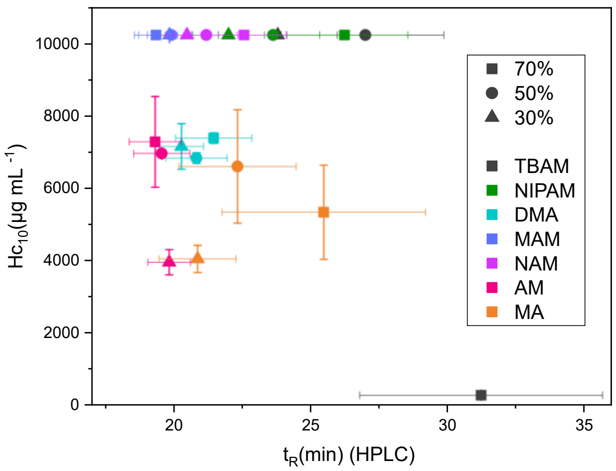

Figure S23: Correlation of amphiphilicity (represented by retention time in HPLC) with hemolysis of copolymers.

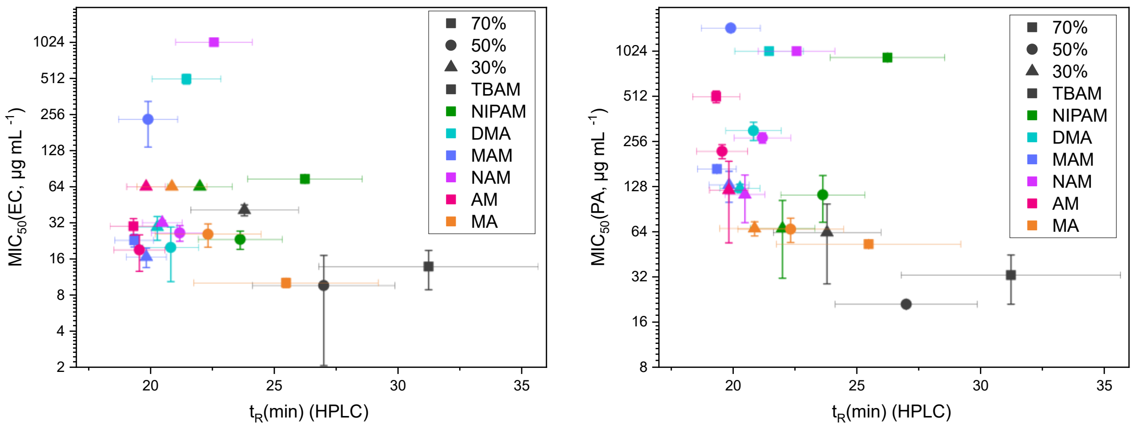

Figure S24: Correlation of amphiphilicity (represented by retention time in HPLC) with MIC_{50} of copolymers.

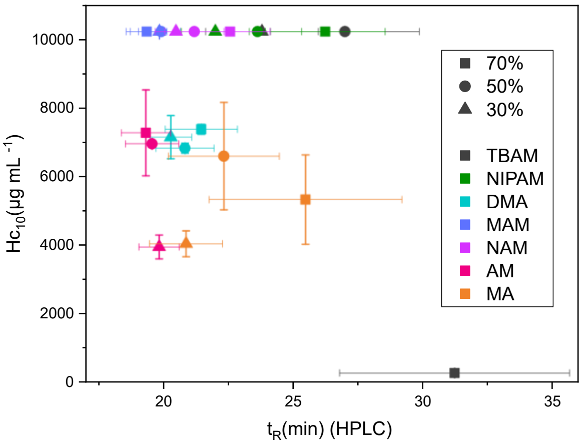

Figure S25: Correlation of amphiphilicity (represented by retention time in HPLC) with CC_{50} of copolymers.

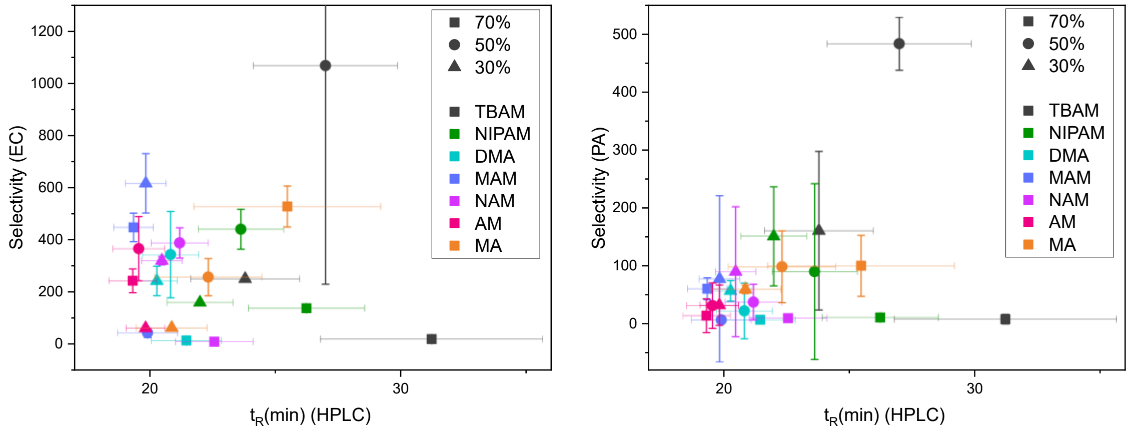

Figure S26: Correlation of amphiphilicity (represented by retention time in HPLC) with selectivity of copolymers.

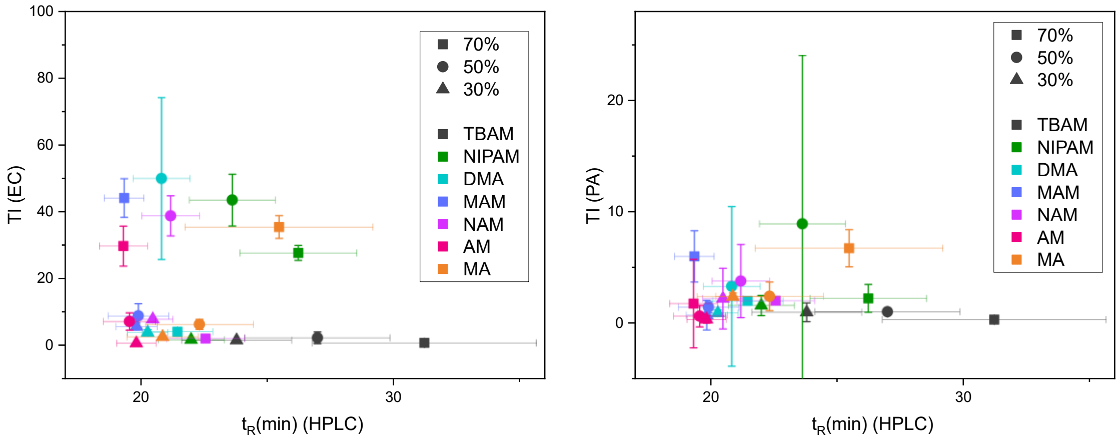

Figure S27: Correlation of amphiphilicity (represented by retention time in HPLC) with TI of copolymers.

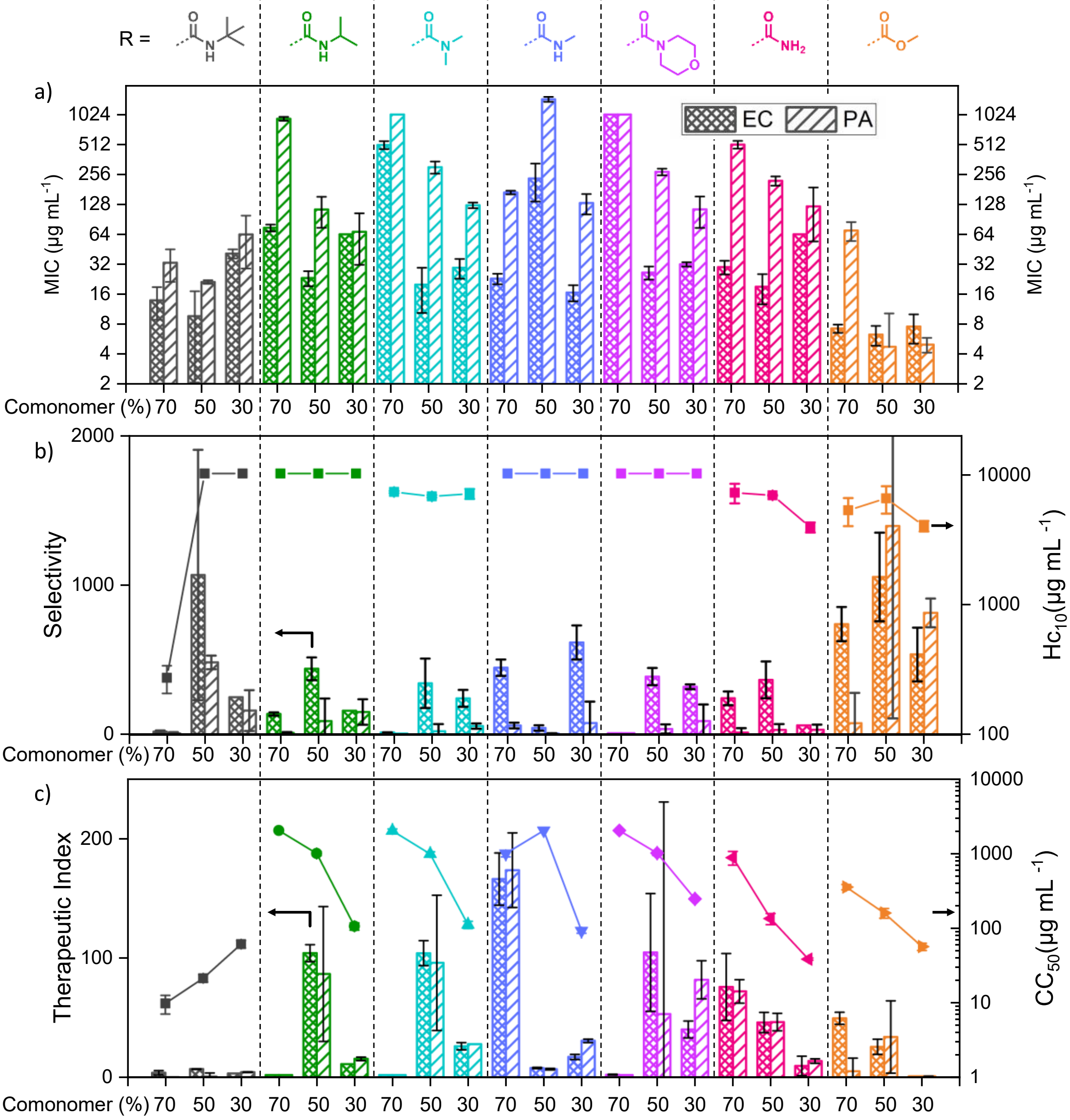

Figure S28: Overview of biological data for copolymers. a) MIC_{50} values against EC and PA respectively, b) HC_{10} and selectivity values, c) CC_{50} and TI values.

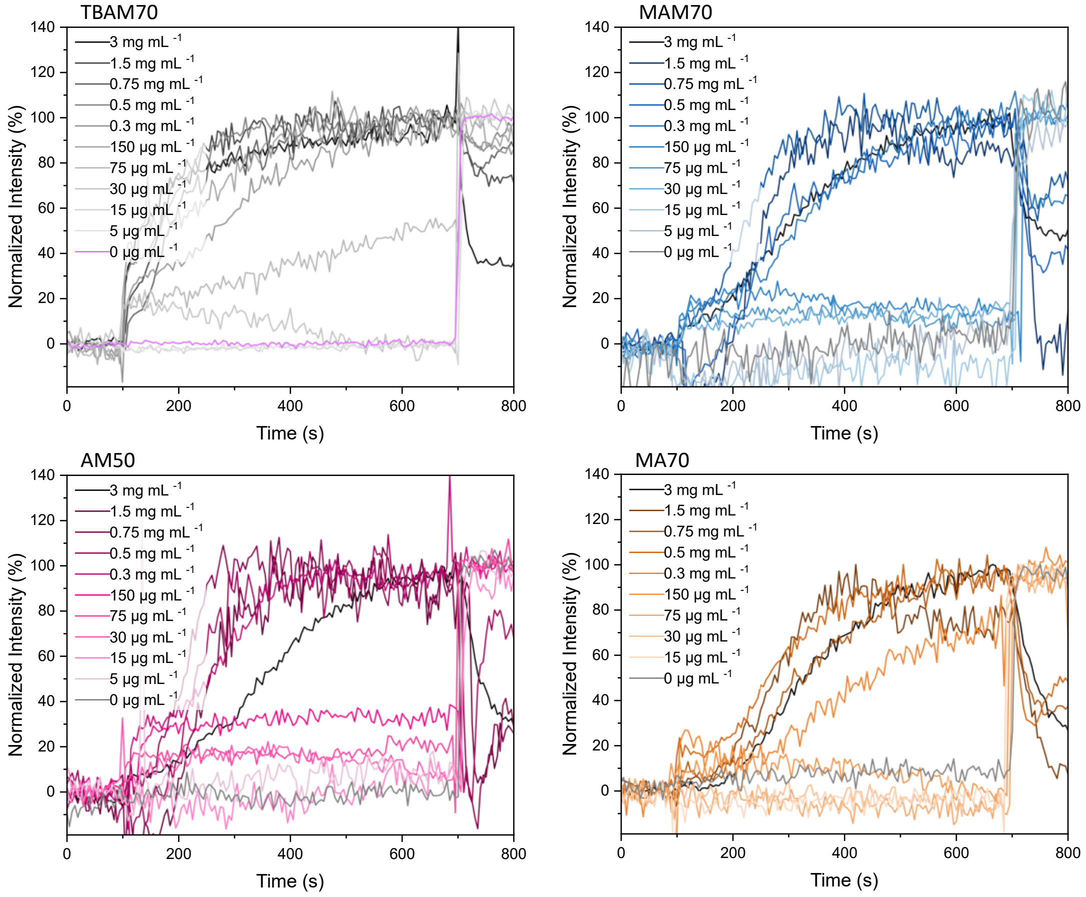

Figure S29: Dye leakage assays of the copoylmers TBAM70, MAM70, AM50, and MA70 against liposomes mimicking *E. coli* (POPE-POPG (8:2)). Copolymers were injected at 100 seconds for each run and Triton X, used as positive control, was added after 700 seconds.

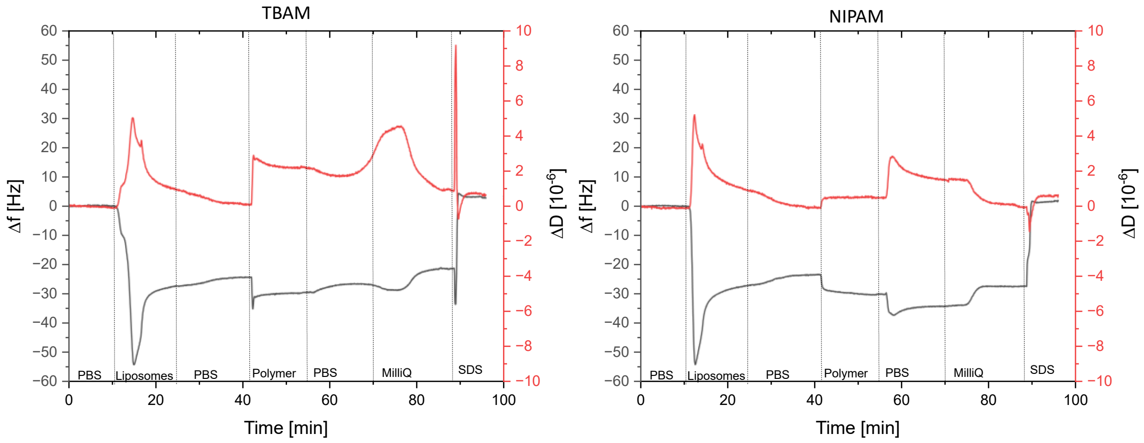

Figure S30: QCM-D measurements of TBAM70 and NIPAM70. Frequency *Δf* and dissipation *ΔD* on SiO2 crystals were initially calibrated with PBS at a constant flow rate of 100 µL min^{-1}. Liposomes suspension (0.5 mg mL^{-1}) composed of DOPC:DOPS (9:1) was then injected to form a solid lipid bilayer (SLB). The

SLB was rinsed with PBS before injection of TBAM70 and NIPAM70 (150 µg mL^{-1}). Subsequent rinses of the polymers, first with PBS and then with MilliQ water were applied. Finally the surfactant sodium dodecyl sulfate (2%) was applied to clear the crystal surface.

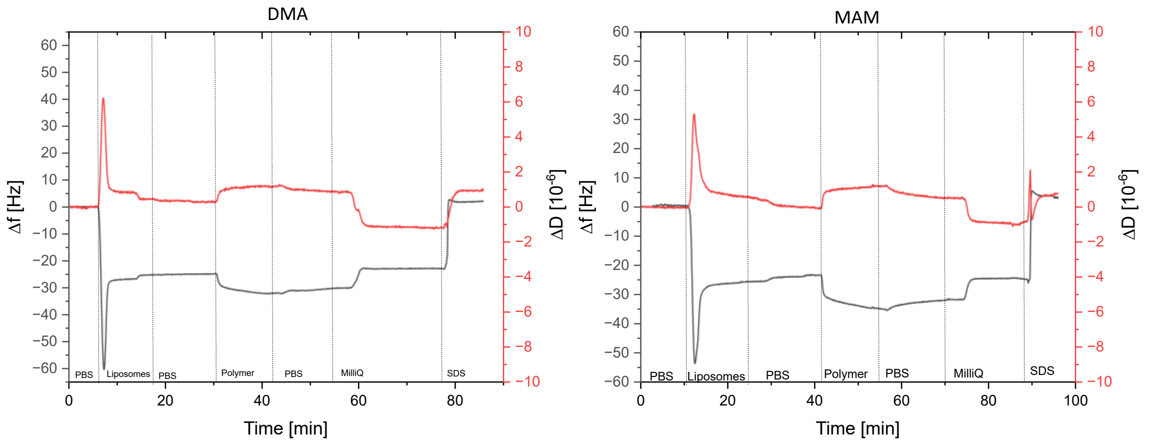

Figure S31: QCM-D measurements of DMA70 and MAM70. Frequency *Δf* and dissipation *ΔD* on SiO_2 crystals were initially calibrated with PBS at constant flow rate of 100 µL min^{-1}. A liposome suspension (0.5 mg mL^{-1}) composed of DOPC:DOPS (9:1) was then injected to form a solid lipid bilayer (SLB). The SLB was rinsed with PBS before injection of polymers DMA70 and MAM70 (150 µg mL^{-1}). Subsequent rinses of the polymers, first with PBS and then with MilliQ water were applied. Finally, the surfactant sodium dodecyl sulfate (2%) was applied to clean the crystal surface.

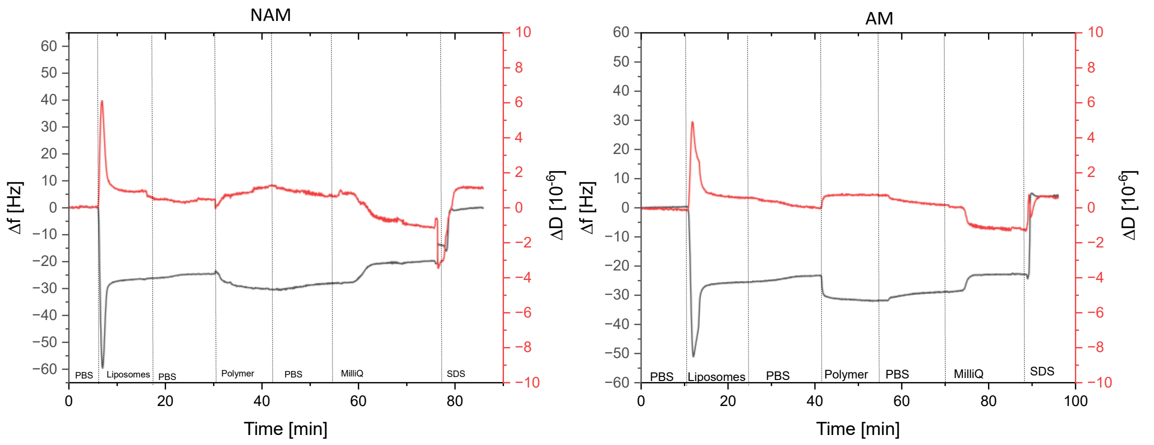

Figure S32: QCM-D measurements of NAM70 and AM70. Frequency *Δf* and dissipation *ΔD* on SiO_2 crystals were initially calibrated with PBS at a constant flow rate of 100 µL min^{-1}. A liposome suspension (0.5 mg mL^{-1}) composed of DOPC:DOPS (9:1) was then injected to form a solid lipid bilayer (SLB). The SLB was rinsed with PBS before injection of polymers NAM70 and AM70 (150 µg mL^{-1}). Subsequent rinses of the polymers, first with PBS and then with MilliQ water were applied. Finally, the surfactant sodium dodecyl sulfate (2%) were applied to clean the crystal surface.

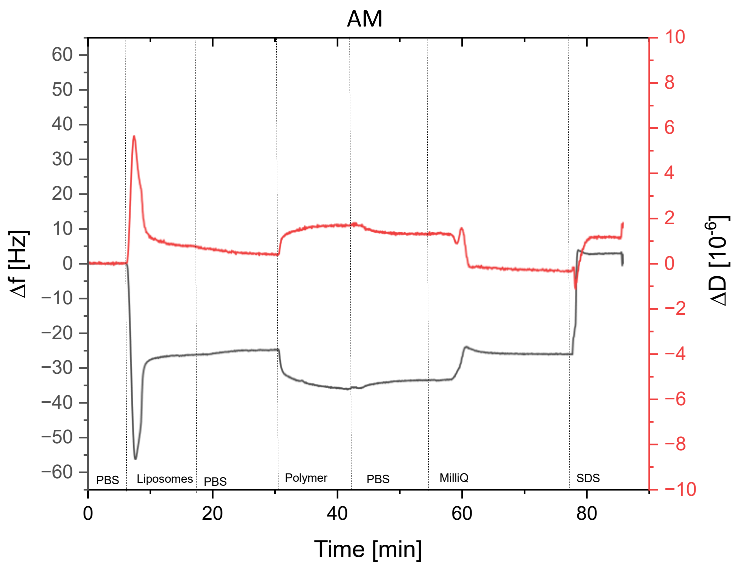

Figure S33: QCM-D measurements of MA70. Frequency *Δf* and dissipation *ΔD* on SiO_2 crystals were initially calibrated with PBS at a constant flow rate of 100 µL min^{-1}. A liposomes suspension (0.5 mg mL^{-1}) composed of DOPC:DOPS (9:1) was then injected to form a solid lipid bilayer (SLB). The SLB was rinsed with PBS before injection of polymer MA70 (150 µg mL^{-1}). Subsequent rinses of the polymer, first with PBS and then with MilliQ water were applied. Finally, h the surfactant sodium dodecyl sulfate (2%) were applied to clean the crystal surface.

Peak Analysis

Data Set:[Book1]Sheet1!D Date:4/23/2024
BaseLine:Constant
Chi^2=3.86925E-04 Adj. R-Square=9.95119E-01 # of Data Points=271
SS=9.67311E-02 Degrees of Freedom=250

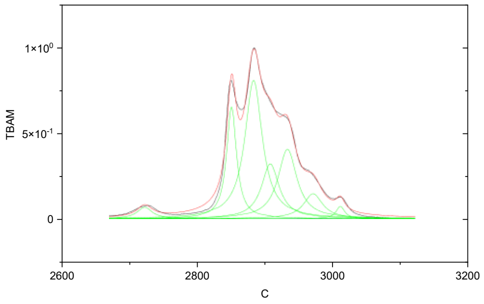

Fitting Results

Peak Index	Peak Type	Area Intg	FWHM	Max Height	Center Grvty	Area IntgP
1	Lorentz	17.35547	17.49588	0.64816	2850.80644	17.06284
2	Lorentz	37.4688	31.06566	0.80307	2883.37976	36.83703
3	Lorentz	7.32143	33.98791	0.14493	2971.26815	7.19799
4	Lorentz	15.076	31.55899	0.3183	2908.05434	14.8218
5	Lorentz	20.24764	33.5654	0.40363	2933.38249	19.90624
6	Lorentz	1.68411	15.41699	0.07165	3011.57643	1.65571
7	Lorentz	2.56157	27.01067	0.06644	2722.49741	2.51838

Peak Analysis

Data Set:[Book1]Sheet1!F Date:4/23/2024
BaseLine:Constant
Chi^2=3.42961E-04 Adj. R-Square=9.95131E-01 # of Data Points=271
SS=8.57403E-02 Degrees of Freedom=250

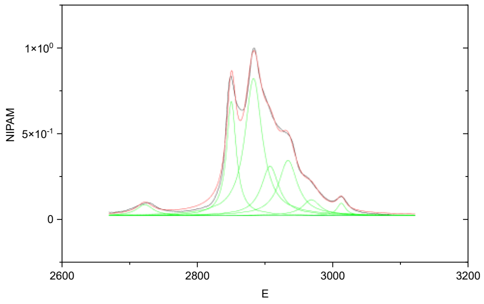

Fitting Results

Peak Index	Peak Type	Area Intg	FWHM	Max Height	Center Grvty	Area IntgP
1	Lorentz	17.42173	17.07701	0.66618	2850.4642	18.81594
2	Lorentz	36.94119	30.71416	0.80041	2883.22839	39.89748
3	Lorentz	4.35782	31.53551	0.09256	2968.6388	4.70656
4	Lorentz	13.5609	31.29889	0.28858	2907.58909	14.64613
5	Lorentz	15.95751	33.12782	0.32212	2934.08033	17.23454
6	Lorentz	1.70115	15.5158	0.07195	3012.72356	1.83729
7	Lorentz	2.64998	28.9178	0.06458	2722.80285	2.86205

Figure S34: Deconvolution of Raman spectra of TBAM70 and NIPAM70.

Peak Analysis

Data Set:[Book1]Sheet1!P Date:4/23/2024
BaseLine:Constant
Chi^2=6.15492E-04 Adj. R-Square=9.92839E-01 # of Data Points=271
SS=1.53873E-01 Degrees of Freedom=250

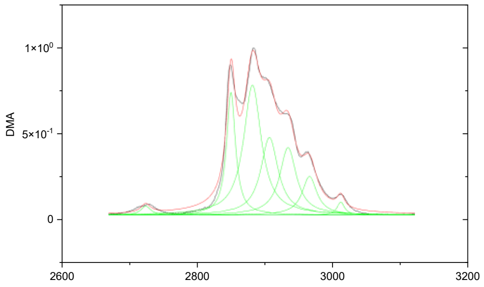

Fitting Results

Peak Index	Peak Type	Area Intg	FWHM	Max Height	Center Grvty	Area IntgP
1	Lorentz	17.23015	15.77066	0.71203	2850.12629	16.75868
2	Lorentz	35.57002	31.38933	0.75489	2881.77308	34.59671
3	Lorentz	8.83434	26.1692	0.22406	2965.92803	8.59261
4	Lorentz	20.8393	30.73977	0.45116	2906.84318	20.26907
5	Lorentz	17.4869	29.70181	0.39169	2934.25829	17.00841
6	Lorentz	1.525	13.33389	0.07473	3012.10735	1.48327
7	Lorentz	1.32757	17.45232	0.05138	2724.3065	1.29125

Peak Analysis

Data Set:[Book1]Sheet1!H Date:4/23/2024
BaseLine:Constant
Chi^2=3.34692E-04 Adj. R-Square=9.95754E-01 # of Data Points=271
SS=8.36729E-02 Degrees of Freedom=250

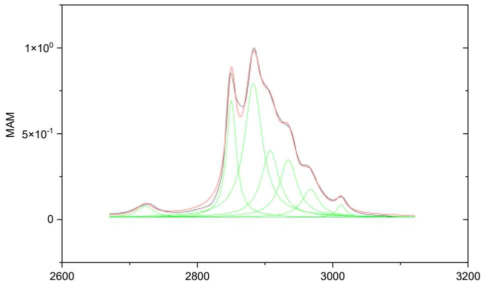

Fitting Results

Peak Index	Peak Type	Area Intg	FWHM	Max Height	Center Grvty	Area IntgP
1	Lorentz	17.81766	17.20419	0.67642	2850.36287	17.6043
2	Lorentz	37.30613	32.00687	0.77716	2882.80234	36.8594
3	Lorentz	7.44452	30.61984	0.16256	2967.31641	7.35538
4	Lorentz	18.38864	31.65662	0.3871	2907.77072	18.16844
5	Lorentz	15.73971	31.41351	0.3342	2934.43385	15.55123
6	Lorentz	1.77991	16.14198	0.07244	3012.4374	1.7586
7	Lorentz	2.73541	29.6057	0.06524	2723.2067	2.70265

Figure S35: Deconvolution of Raman spectra of DMA70 and MAM70.

Peak Analysis

Data Set:[Book1]Sheet1!L Date:4/23/2024
BaseLine:Constant
Chi^2=4.31388E-04 Adj. R-Square=9.94511E-01 # of Data Points=271
SS=1.07847E-01 Degrees of Freedom=250

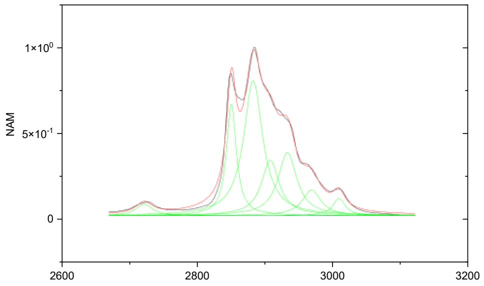

Fitting Results

Peak Index	Peak Type	Area Intg	FWHM	Max Height	Center Grvty	Area IntgP
1	Lorentz	17.21977	17.36185	0.64792	2850.56567	16.54726
2	Lorentz	39.68163	33.85337	0.78369	2882.7024	38.13188
3	Lorentz	7.3786	33.3204	0.14876	2968.7674	7.09043
4	Lorentz	15.35186	31.89953	0.32083	2907.95914	14.7523
5	Lorentz	18.32846	33.42667	0.36681	2933.17929	17.61265
6	Lorentz	3.32146	22.14186	0.09965	3009.16928	3.19174
7	Lorentz	2.78241	29.75872	0.06616	2722.0906	2.67375

Peak Analysis

Data Set:[Book1]Sheet1!J Date:4/23/2024
BaseLine:Constant
Chi^2=5.99002E-04 Adj. R-Square=9.93416E-01 # of Data Points=271
SS=1.49750E-01 Degrees of Freedom=250

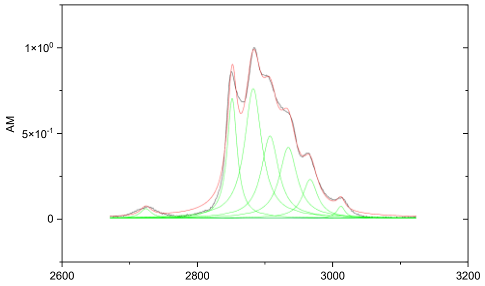

Fitting Results

Peak Index	Peak Type	Area Intg	FWHM	Max Height	Center Grvty	Area IntgP
1	Lorentz	18.60724	17.50822	0.69445	2851.37781	17.45821
2	Lorentz	34.3817	30.39666	0.75243	2882.4169	32.25858
3	Lorentz	8.98987	26.60178	0.22442	2966.30855	8.43473
4	Lorentz	22.03103	30.74061	0.47694	2907.18078	20.67059
5	Lorentz	19.4806	31.64742	0.41068	2934.11951	18.27765
6	Lorentz	1.53034	14.59481	0.06866	3011.69919	1.43584
7	Lorentz	1.56079	19.41752	0.05476	2724.2222	1.46441

Figure S36: Deconvolution of Raman spectra of NAM70 and AM70.

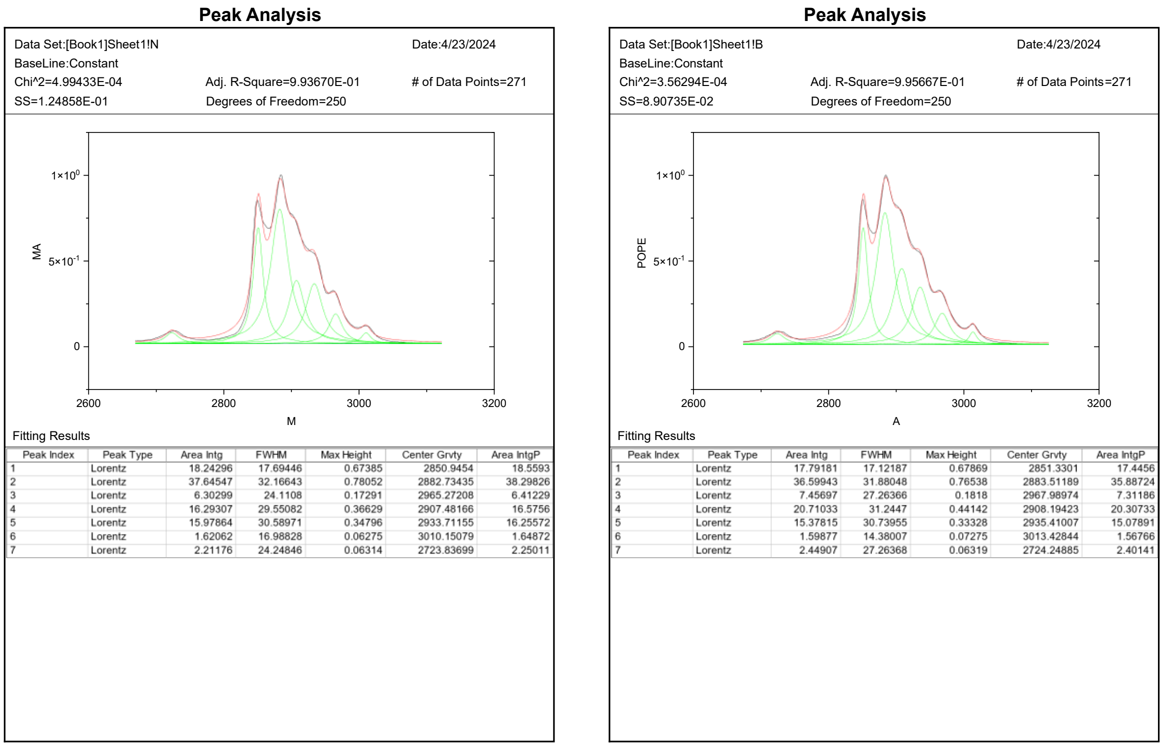

Fitting Results

Peak Index	Peak Type	Area Intg	FWHM	Max Height	Center Grvty	Area IntgP
1	Lorentz	18.24296	17.69446	0.67385	2850.9454	18.5593
2	Lorentz	37.64547	32.16643	0.78052	2882.73435	38.29826
3	Lorentz	6.30299	24.1108	0.17291	2965.27208	6.41229
4	Lorentz	16.29307	29.55082	0.36629	2907.48166	16.5756
5	Lorentz	15.97864	30.58971	0.34796	2933.71155	16.25572
6	Lorentz	1.62062	16.98828	0.06275	3010.15079	1.64872
7	Lorentz	2.21176	24.24846	0.06314	2723.83699	2.25011

Fitting Results

Peak Index	Peak Type	Area Intg	FWHM	Max Height	Center Grvty	Area IntgP
1	Lorentz	17.79181	17.12187	0.67869	2851.3301	17.4456
2	Lorentz	36.59943	31.88048	0.76538	2883.51189	35.88724
3	Lorentz	7.45697	27.26366	0.1818	2967.98974	7.31186
4	Lorentz	20.71033	31.2447	0.44142	2908.19423	20.30733
5	Lorentz	15.37815	30.73955	0.33328	2935.41007	15.07891
6	Lorentz	1.59877	14.38007	0.07275	3013.42844	1.56766
7	Lorentz	2.44907	27.26368	0.06319	2724.24885	2.40141

Figure S37: Deconvolution of Raman spectra of MA70 and POPE.

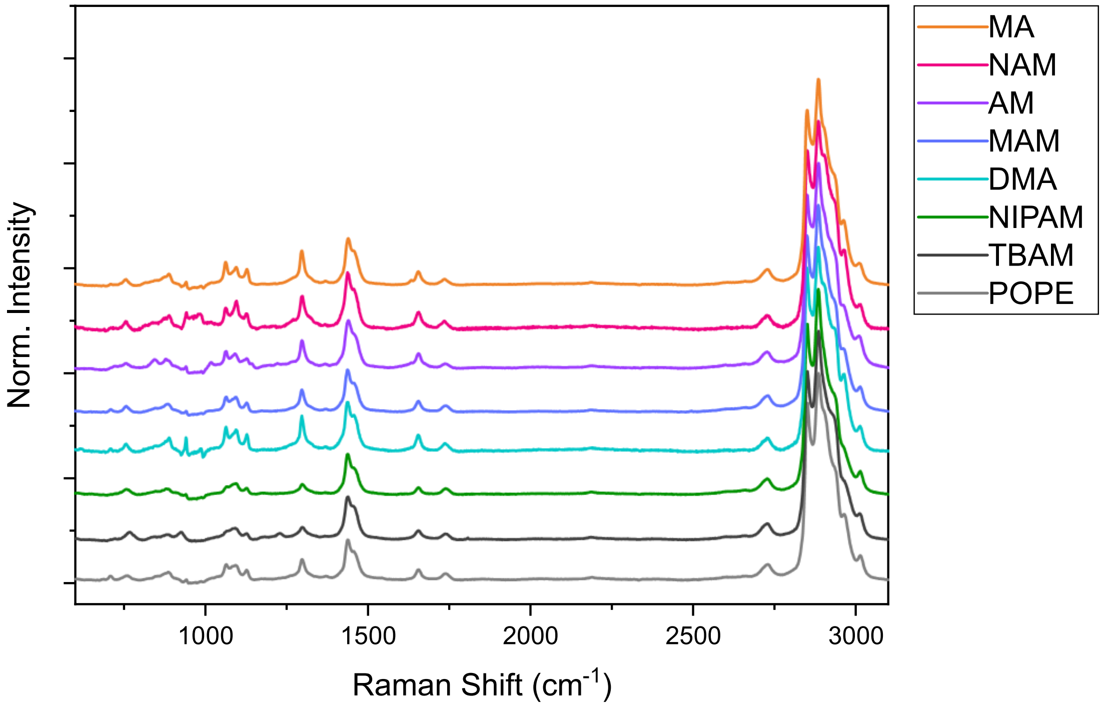

Figure S38: Raman spectra of a 1:2 mixture of copolymers (with 70% of comonomer ratio) and POPE.

Raman spectroscopy is a valuable technique to investigate the interaction of lipids (that can mimic the properties of cell membranes) with external agents, in our case APs. In this way, when the AP is in

contact with the lipidic membrane, Raman scattering is of fundamental interest because it is a measurement sensitive to conformational states of the hydrocarbon chains and its interactions with molecules in this layer. Of the obtained spectra, the main vibrations of interest are obtained in the region between 2800 to 3000 cm^{-1}, characteristic of C-H vibrational modes, whereas the peak at 2850 cm^{-1} (symmetric CH_2 stretching) and 2880 cm^{-1} (asymmetric CH_2 stretching) are sensitive to the changes in the phospholipid's interactions. It is assumed that the symmetric mode intensity (I_{sCH}) is not dependent on the lateral order and the conformation of the lipids. However, the asymmetric mode (I_{asCH}) is very dependent on this. In this way, the higher the order of the lipid membrane, the higher the ratio of I_{asCH}/I_{sCH}. For this reason, we measured the Raman spectra of the mixture of the AP with POPE as a way of mimicking the interaction of the AP with the cell walls. The value for the phospholipid without the AP is set to 1 as a reference. Values that are higher than 1 are indicative of a more organized lipid structure, while values below 1 correspond to less organization.

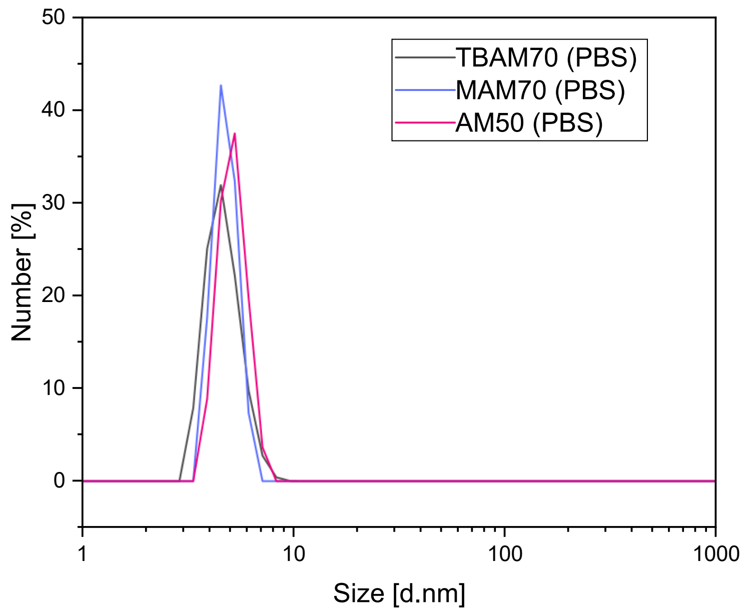

Figure S39: Dynamic light scattering spectra representing size distribution by number of copolymer solution at 1 mg mL^{-1} in PBS buffer at pH 7.4.

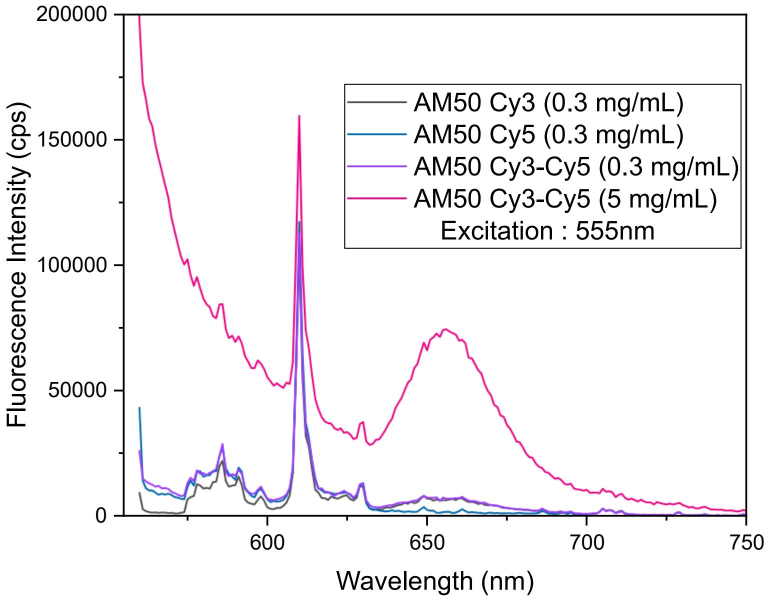

Figure S40: Emission acquisition spectra of labeled polymer in PBS medium alone and in mixtures at low and high concentration. At the maximum excitation wavelength of the donor at 555nm (Exc_{max} of Cy3), FRET emission can be detected at around 655 nm (emission of acceptor Cy5). Energy transfer is only observed in a significant manner when both dyes are present in high concentrations.

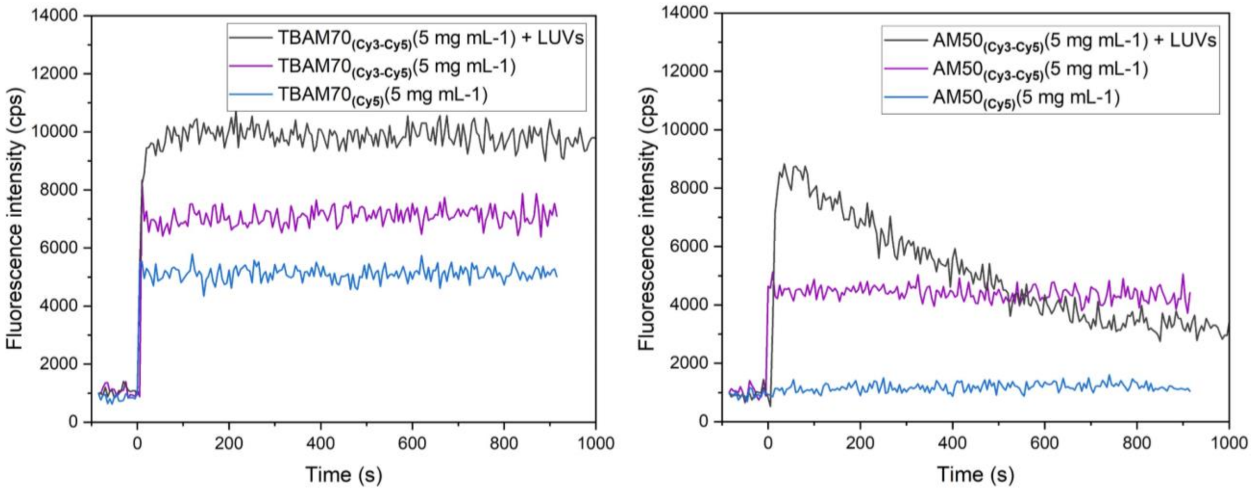

Figure S41: Time-based fluorescence intensity spectra of LUVs incubated with a mixture of copolymers labelled with donor and acceptor dye ($P_{(Cy3-Cy5)}$+ LUVs), of polymer mixture alone($P_{(Cy3-Cy5)}$), and of acceptor polymer alone($P_{(Cy5)}$) in PBS. Time of 0s corresponds to the injection of the polymers. The

spectra were recorded at the acceptor maximal emission (657 nm) upon excitation of the donor dye (Exc $_{max}$=555 nm).

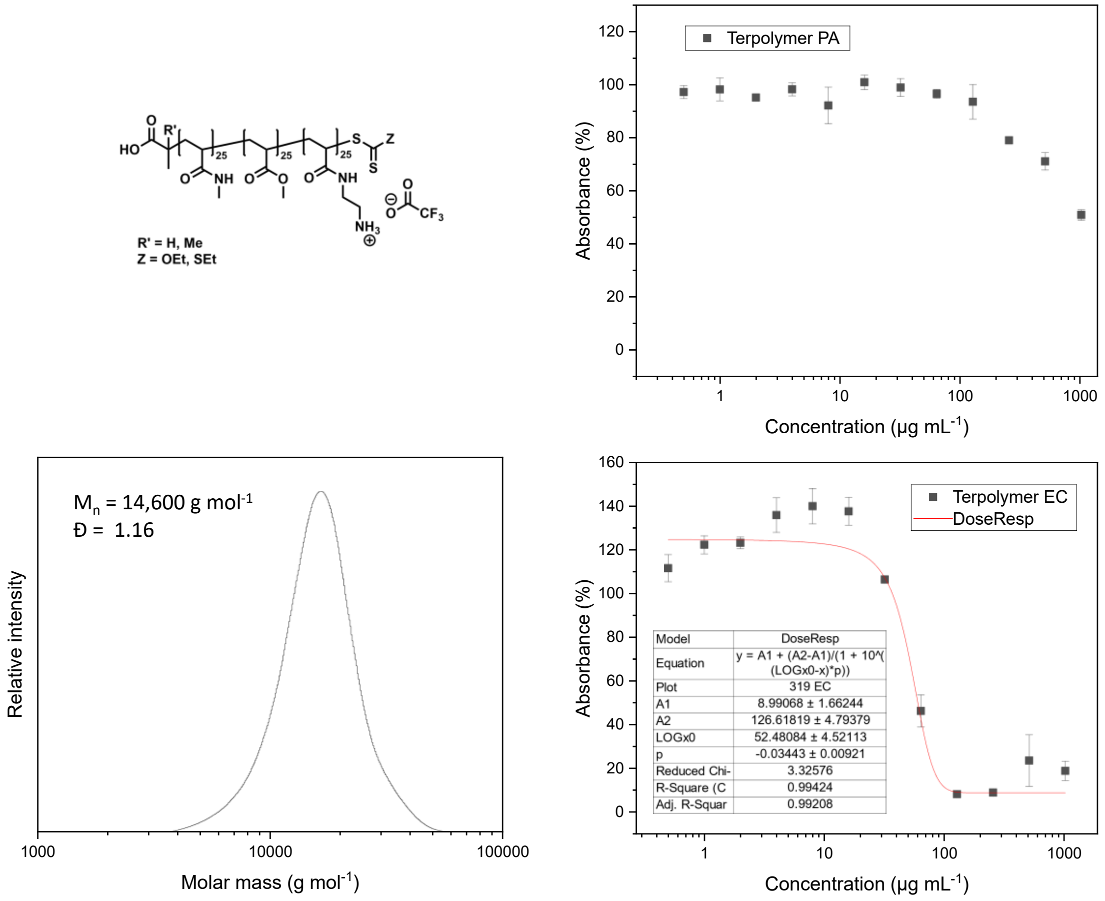

Figure S42: SEC curve of a copolymer containing AEAM, AM and MAM and respective MIC determination against EC and PA using dose response fit in origin software.

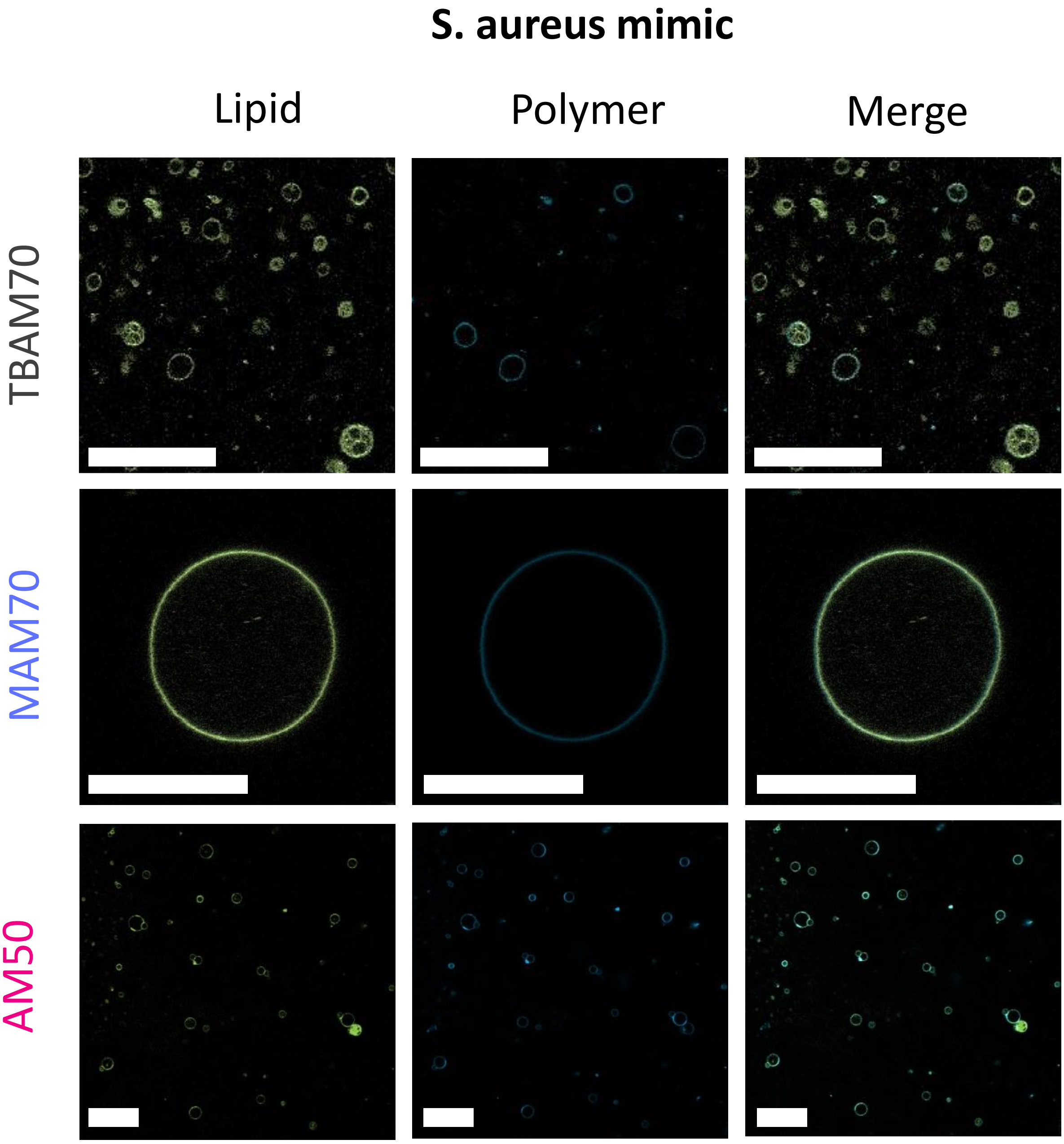

Figure S43: Microscopic images of labelled GUVs mimicking S. aureus membranes and labelled polymers. Images were contrast enhanced to increase visibility; scale bars are 40 µm.

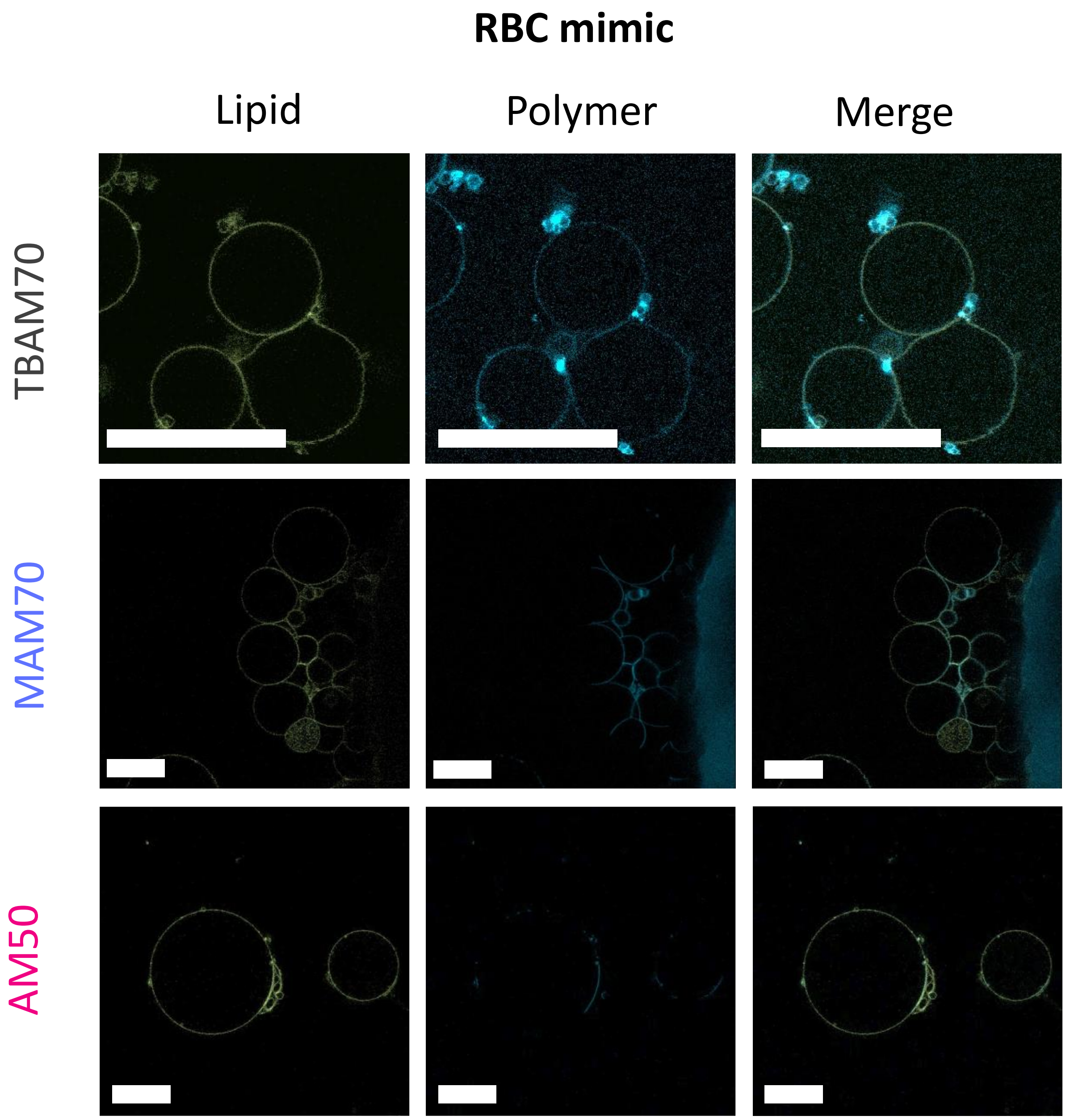

Figure S44: Microscopic images of labelled GUVs mimicking RBC membranes and labelled polymers. Images were contrast enhanced to increase visibility; scale bars are 40 µm.

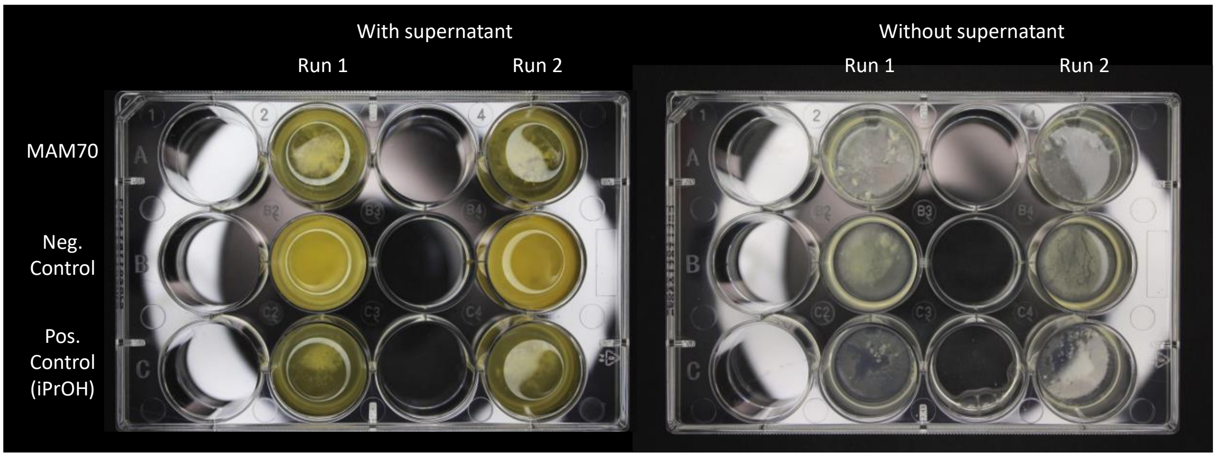

Figure S45: Photographs of well plates containing biofilms after 24 h incubation with the respective solution (MAM70 (1024 µg mL^{-1}), negative control (ddH2O), positive control (70% (v/v) 2-propanol in water). Photographs were taken directly after incubation including the supernatant (left), as well as after removal of the supernatant, washing, and before labelling (right).

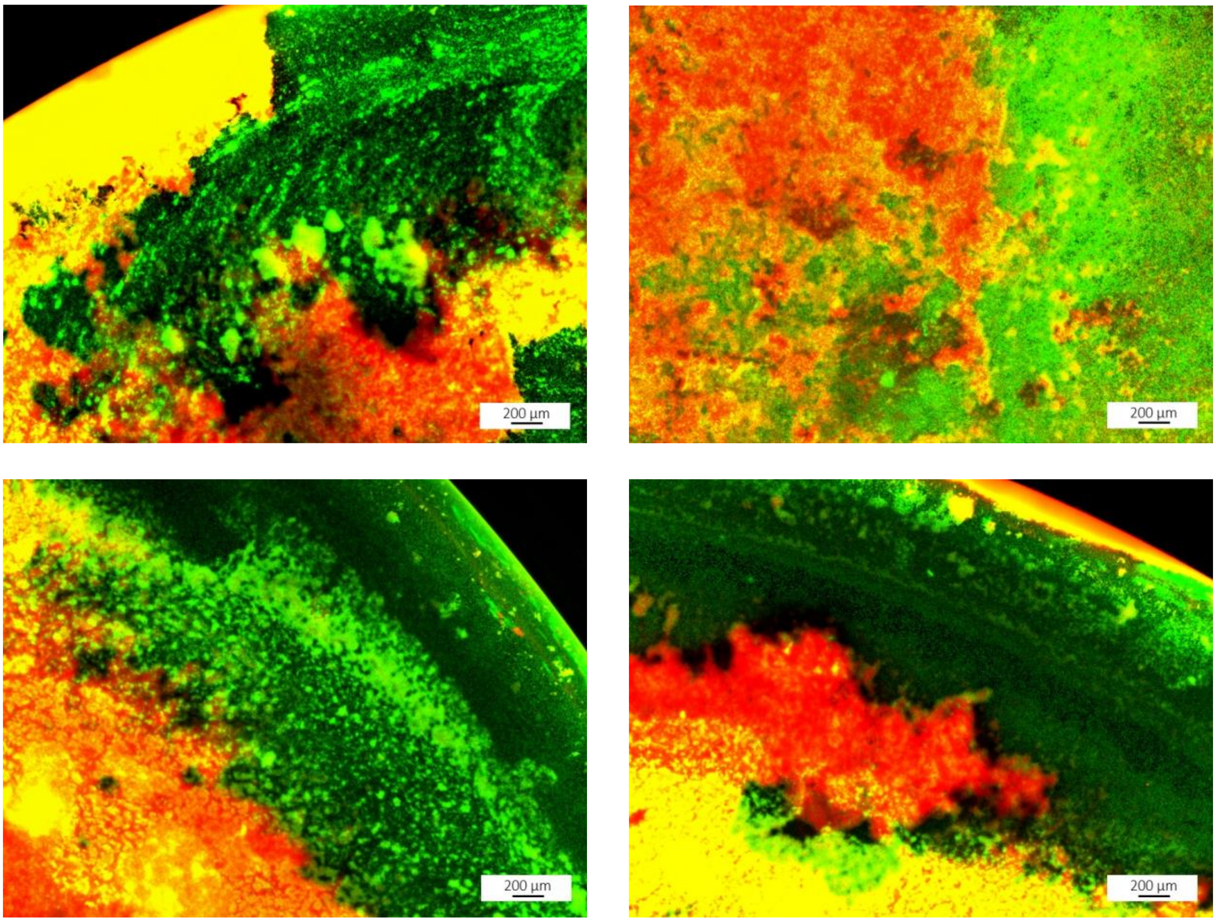

Figure S46: Microscopic images of representative regions of biofilms treated with MAM70 (1024 µg mL^{-1}, after 24 h). Bacteria were stained using Syto-9 (green, viable bacteria) and propidium iodide (red, membrane permeabilization).

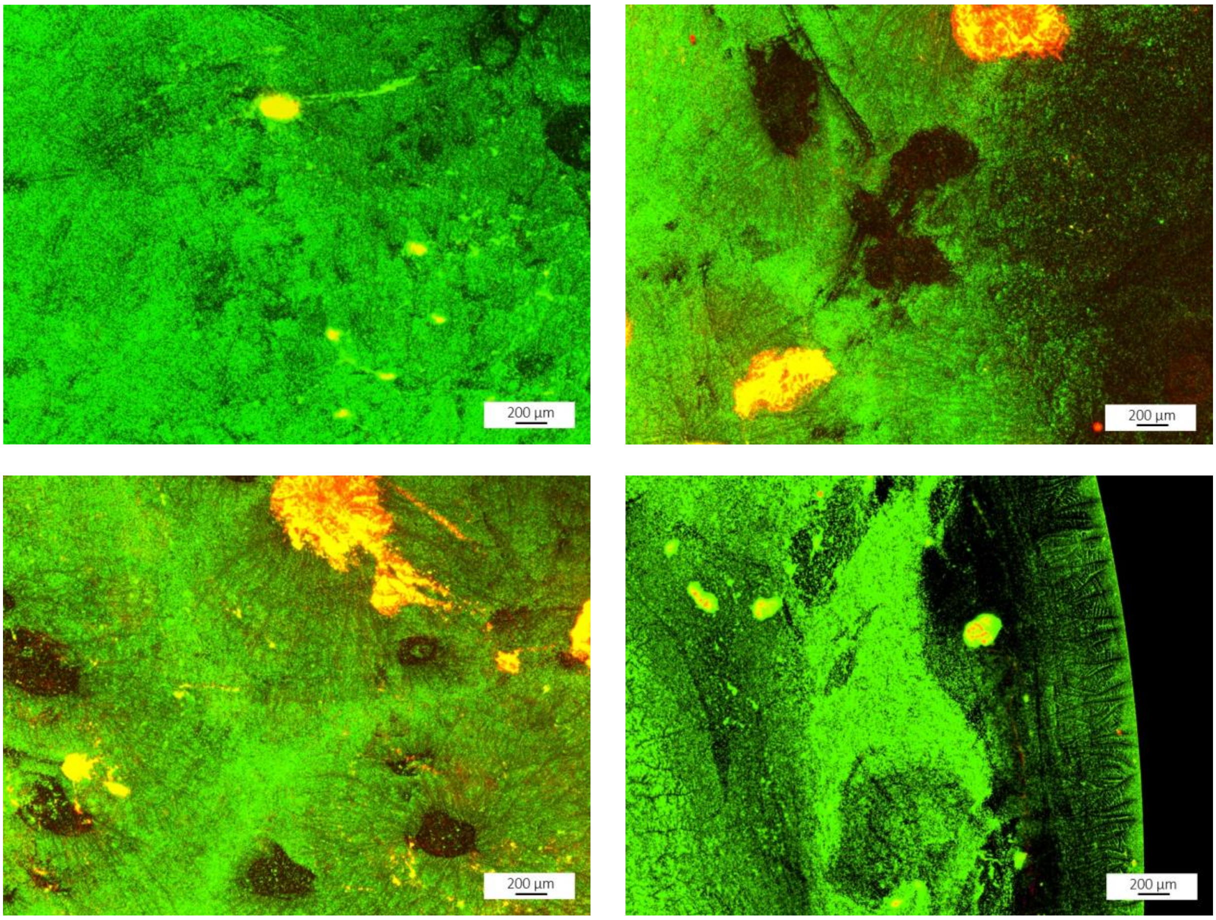

Figure S47: Microscopic images of representative regions of biofilms treated with ddH_2O (negative control, after 24 h). Bacteria were stained using Syto-9 (green, viable bacteria) and propidium iodide (red, membrane permeabilization).

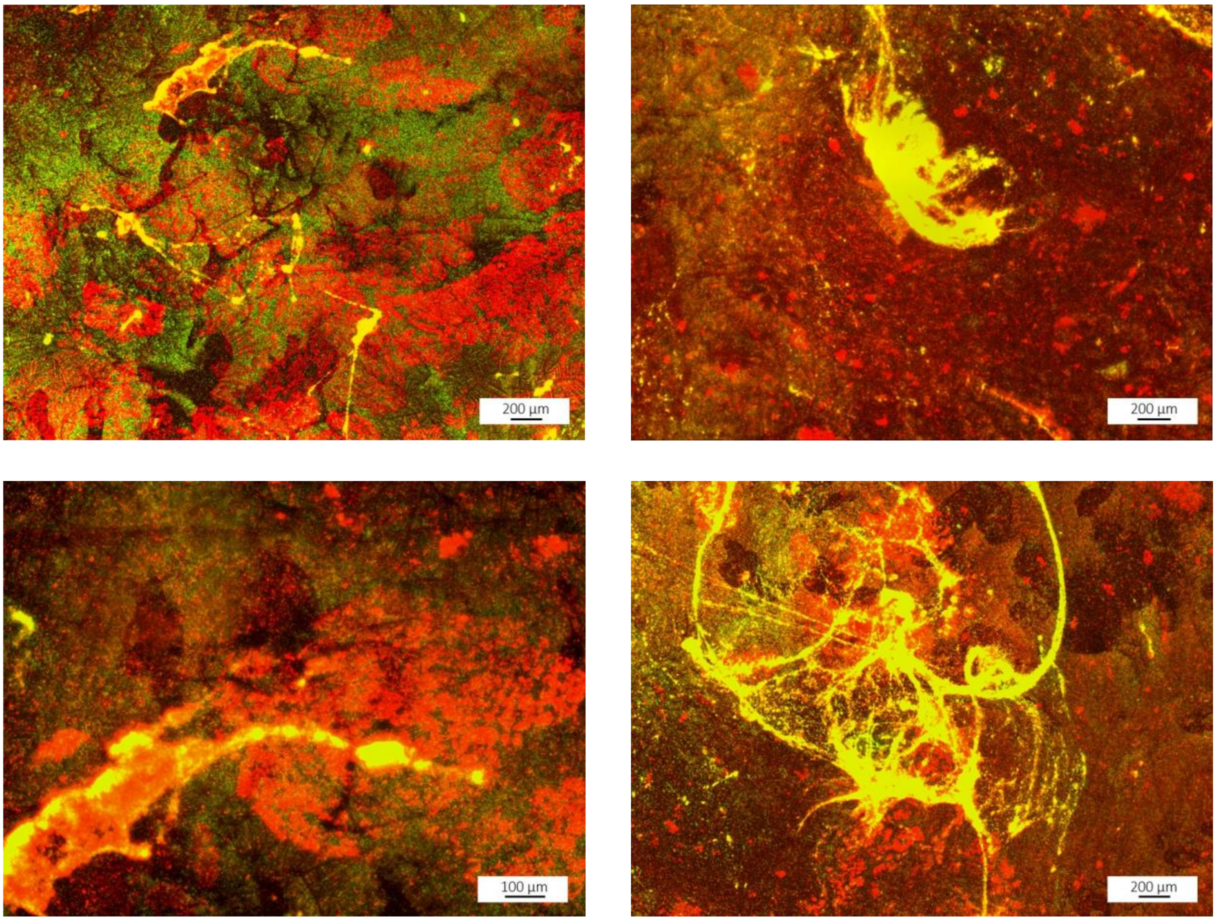

Figure S48: Microscopic images of representative regions of biofilms treated with 70% (v/v) 2-propanol in water (positive control, after 24 h). Bacteria were stained using Syto-9 (green, viable bacteria) and propidium iodide (red, dead bacteria).

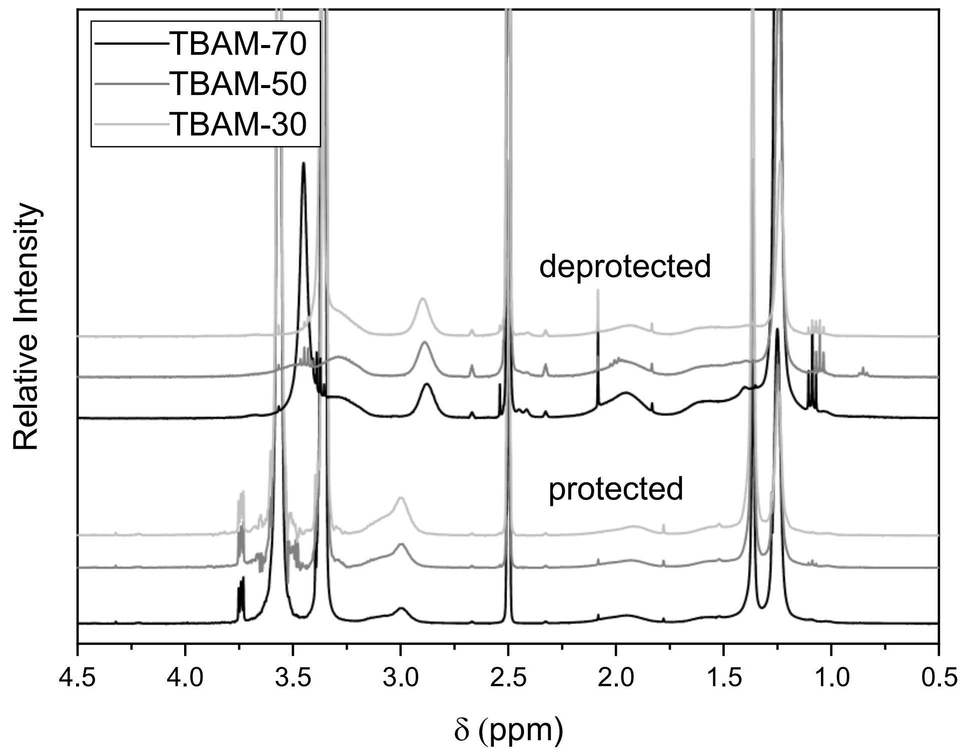

Figure S49: ^{1}H-NMR spectra (DMSO-D_6) of polymers with TBAM before and after deprotection.

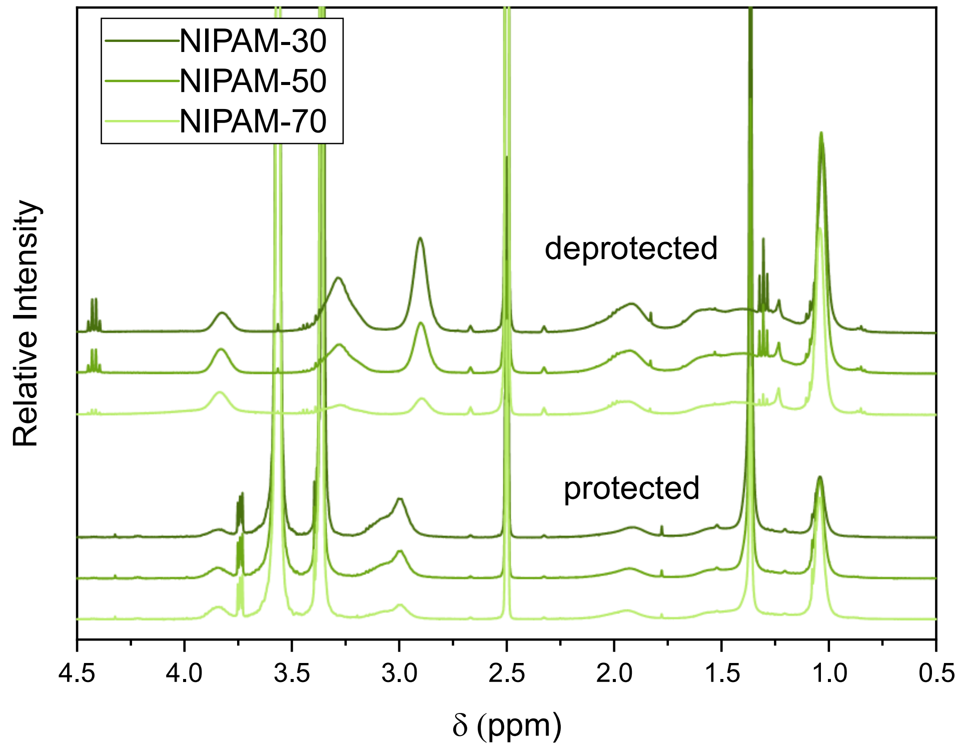

Figure S50: ^{1}H-NMR spectra (DMSO-D_6) of polymers with NIPAM before and after deprotection.

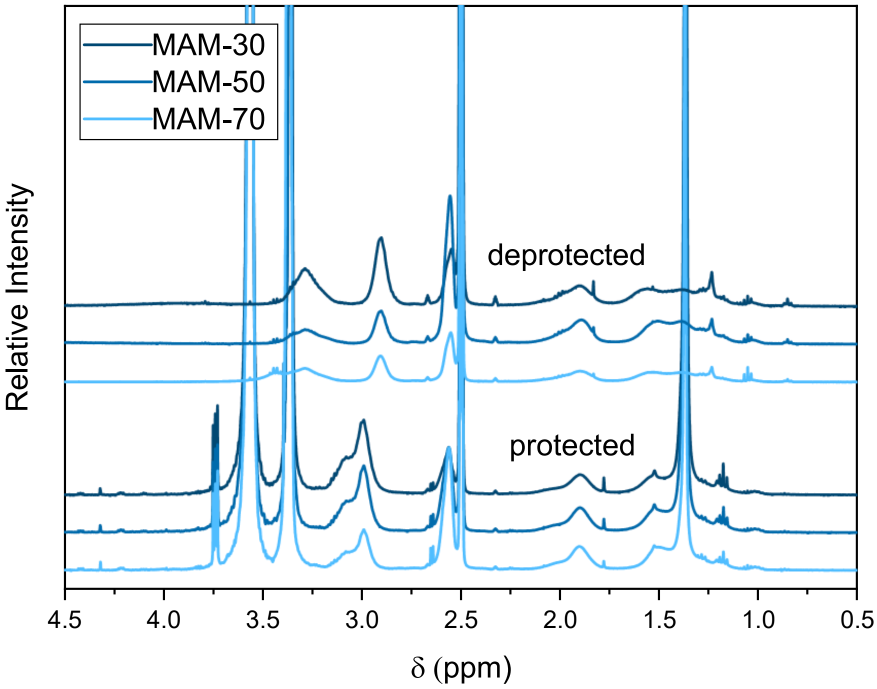

Figure S51: ^{1}H-NMR spectra (DMSO-D$_6$) of polymers with MAM before and after deprotection.

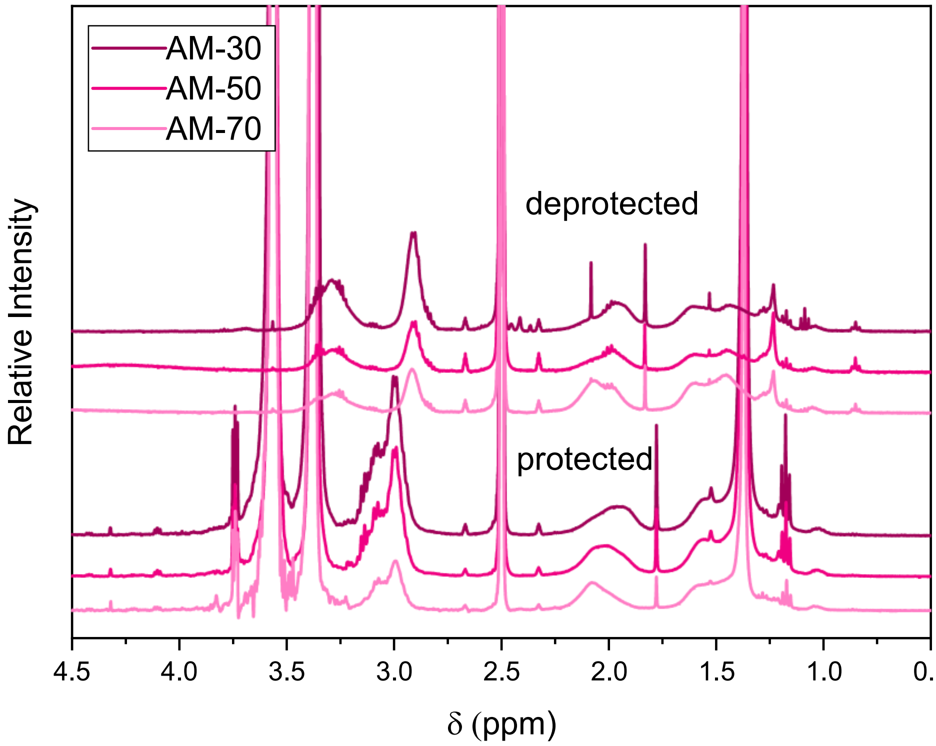

Figure S52: ^{1}H-NMR spectra (DMSO-D$_6$) of polymers with AM before and after deprotection.

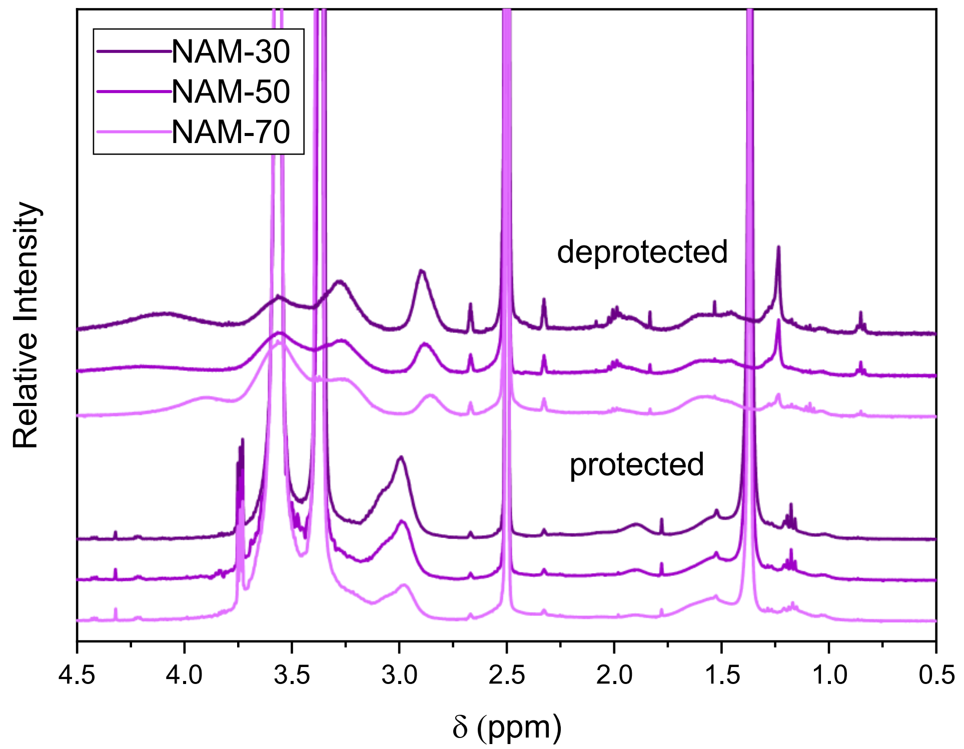

Figure S53: ^{1}H-NMR spectra (DMSO-D_6) of polymers with NAM before and after deprotection.

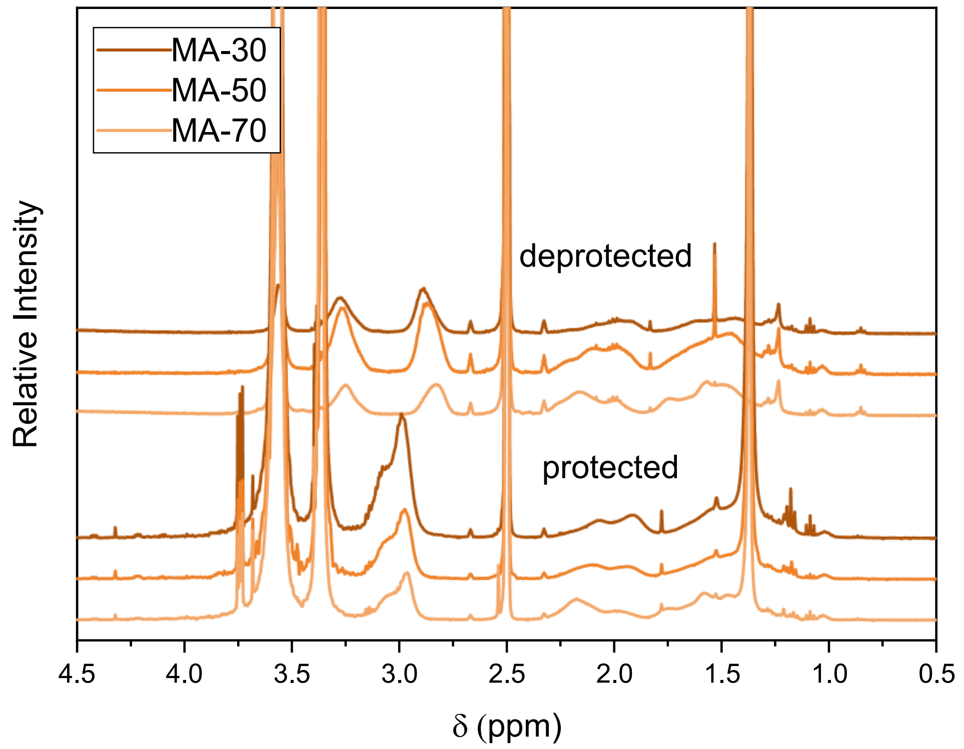

Figure S54: ^{1}H-NMR spectra (DMSO-D_6) of polymers with MA before and after deprotection.

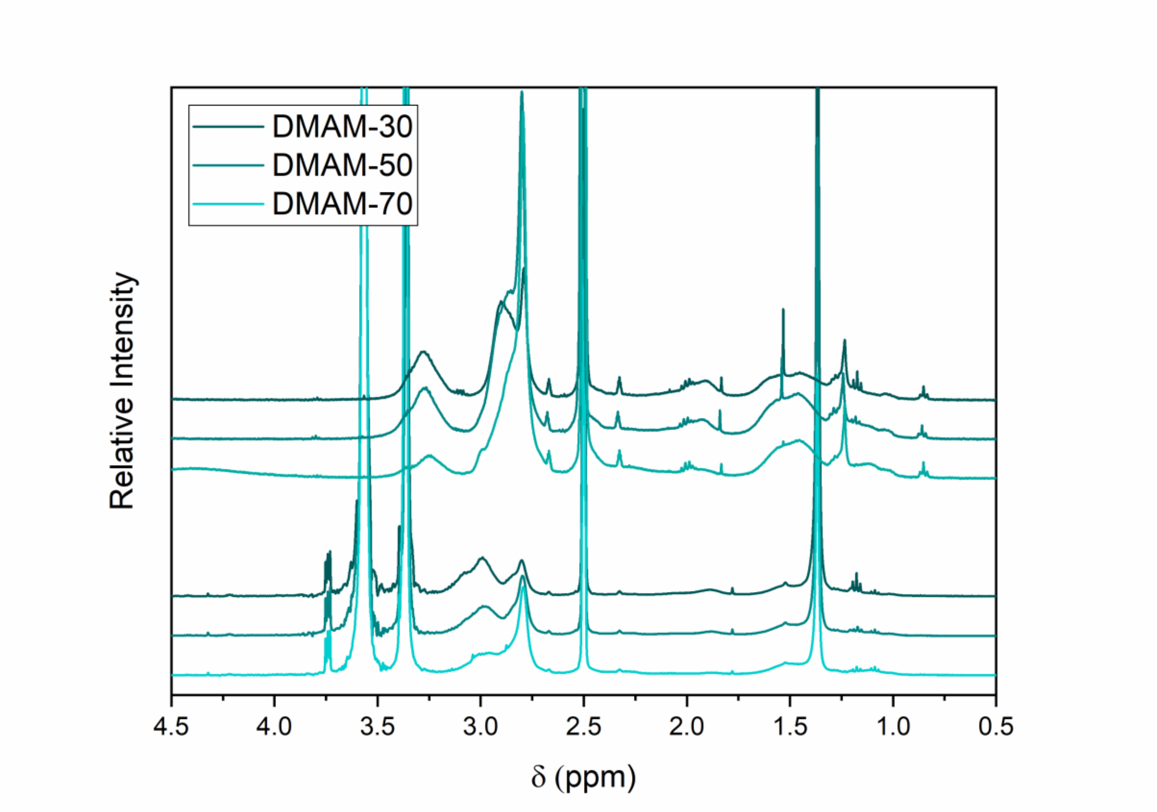

Figure S55: ^{1}H-NMR spectra (DMSO-D_6) of polymers with DMA before and after deprotection.

Supplementary Tables

Table S2: Analytical data for polymers before and after deprotection

Sample	(Boc)AEAM[a] (%)	Comonomer[a] (%)	Before deprotection[b] M_n (g mol^{-1})	Before deprotection[b] Đ	After deprotection[c] M_n (g mol^{-1})	After deprotection[c] Đ	DP[d]
TBAM 70	30	70	12,300	1.13	12,200	1.23	74
TBAM 50	51	49	11,900	1.14	17,500	1.17	74
TBAM 30	72	28	11,700	1.12	19,500	1.15	74
NIPAM 70	32	68	8,600	1.18	15,500	1.16	73
NIPAM 50	54	44	9,800	1.17	18,800	1.16	73
NIPAM 30	68	32	10,700	1.15	21,000	1.15	74
DMAM 70	29	71	7,900	1.12	16,300	1.13	71
DMAM 50	48	52	9,200	1.13	19,000	1.14	73
DMAM 30	69	31	10,000	1.14	21.200	1.11	74
MAM 70	30	70	5,100	1.2	17,200	1.15	72
MAM 50	47	53	-*	-*	15,600	1.17	73
MAM 30	67	33	8,100	1.18	18,200	1.15	73
NAM 70	32	68	9,600	1.15	15,200	1.14	74
NAM 50	52	48	10,500	1.12	16,600	1.16	74
NAM 30	73	27	10,300	1.13	18,700	1.17	74
AM 70	31	69	-*	-*	15,600	1.16	74
AM 50	51	49	4,600	1.23	16,300	1.19	62
AM 30	71	29	6,600	1.17	17,900	1.18	65
MA 70	30	70	10,400	1.16	13,900	1.14	68
MA 50	52	48	11,000	1.12	16,300	1.17	72
MA 30	72	28	11,500	1.14	19,000	1.16	73

a) Determined by ^{1}H-NMR spectroscopy (400 MHz, DMSO-D_6)

b) Determined by SEC (THF, PS calibration)

c) Determined by SEC (Water (0.3% formic acid, 0.1 M NaCl), PEG calibration)

d) Determined based on the final conversion and initial feed ratios of the polymers

* Not determined as the polymer was not soluble in the eluent

Table S3: Bioactivity of all copolymers.

Sample	MIC_{50} (*E. coli*)		MIC_{50} (PA)		HC_{10}		Selectivtiy (*E. coli*)		Selectivtiy (PA)		CC_{50}		TI (*E. coli*)		TI (MRSA)	
	(μg mL^{-1})	±	(μg mL^{-1})	±	(μg mL^{-1})	±		±		±	(μg mL^{-1})	±		±		±
TBAM 70	13.8	5.0	33.1	11.9	273	66	20	9	8	4	9.8	2.8	0.7	0.3	0.3	0.1
TBAM 50	9.6	7.5	21.2	0.9	10240		1068	838	483	21	21.3	2.4	2.2	1.8	1.0	0.1
TBAM 30	40.8	4.3	63.7	34.7	10240		251	26	161	88	61.3	7.1	1.5	0.2	1.0	0.5
NIPAM 70	74.2	6.0	927.7	41.9	10240		138	11	11	0	>2048	0.0	27.6	2.2	2.2	0.1
NIPAM 50	23.2	4.0	113.3	38.9	10240		441	76	90	31	1008	71.5	43.4	8.1	8.9	3.1
NIPAM 30	64.0		67.7	36.2	10240		160	0	151	81	106	11.7	1.7	0.2	1.6	0.9
DMAM 70	505.5	47.4	1024.0		7389	147	15	1	7	0	2032	119	4.0	0.4	2.0	0.1
DMAM 50	19.9	9.6	302.4	42.1	6836	148	344	165	23	3	992	67.3	49.9	24.2	3.3	0.5
DMAM 30	29.5	6.6	125.2	8.2	7157	630	243	58	57	6	111	12.5	3.8	0.9	0.9	0.1
MAM 70	22.9	2.8	168.4	7.1	10240		448	54	61	3	1006	100	44.0	6.9	6.0	0.6
MAM 50	232.5	95.8	1455.9	83.3	10240		44	18	7	0	>2048	0.0	8.8	3.6	1.4	0.1
MAM 30	16.6	3.1	131.8	30.7	10240		617	114	78	18	92.6	7.1	5.6	1.1	0.7	0.2
NAM 70	1024.0	+	1024.0		10240		10		10	0	>2048	0.0	2.0		2.0	0.0
NAM 50	26.4	3.9	271.2	21.5	10240		388	58	38	3	1020	76.4	38.7	6.5	3.8	0.4
NAM 30	32.0	1.6	113.7	39.7	10240		320	16	90	31	248	9.9	7.8	0.5	2.2	0.8
AM 70	29.9	4.7	509.9	45.8	7283	1255	243	57	14	3	887	186	29.6	7.8	1.7	0.4
AM 50	19.0	6.4	220.6	23.6	6962	63	366	123	32	3	135	23.1	7.1	2.7	0.6	0.1
AM 30	64.0		121.8	67.6	3953	351	62	5	32	18	38.5	1.3	0.6	0.0	0.3	0.2
MA 70	10.1	0.9	53.2	2.1	5335	1302	528	137	100	25	357	29.6	35.3	4.3	6.7	0.6
MA 50	25.7	5.6	66.9	12.3	6603	1570	257	83	99	30	160	23.9	6.2	1.7	2.4	0.6
MA 30	64.0		67.7	7.3	4045	376	63	6	60	9	57	6.4	0.9		0.8	0.1
MA-MAM-AEAM	50.6	3.8	>1024		981	86										

References

1. X. Cao; D. Horák; Z. An; Z. Plichta, *J. Polym. Sci., Part A: Polym. Chem.* **2016,** *54*, 1036-1043.
2. A. N. Baker; T. R. Congdon; S.-J. Richards; P. G. Georgiou; M. Walker; S. Dedola; R. A. Field; M. I. Gibson, *ACS Polymers Au* **2022,** *2*, 69-79.
3. A. Kuroki; P. Sangwan; Y. Qu; R. Peltier; C. Sanchez-Cano; J. Moat; C. G. Dowson; E. G. L. Williams; K. E. S. Locock; M. Hartlieb; S. Perrier, *ACS Appl. Mater. Interfaces* **2017,** *9*, 40117-40126.
4. H. Stein; S. Spindler; N. Bonakdar; C. Wang; V. Sandoghdar, *Frontiers in Physiology* **2017,** *8*.
